# Botany, traditional uses, phytochemistry, pharmacology, toxicology and processing of *Rhizoma alismatis*: a review

**DOI:** 10.3389/fphar.2025.1722483

**Published:** 2025-12-04

**Authors:** Tianhan Pan, Ruonan Tang, Jiawen Wang, Jia Gao, Fusheng Jiang, Buyang Chen

**Affiliations:** 1 School of Stomatology, Zhejiang Chinese Medical University, Hangzhou, Zhejiang, China; 2 School of Life Sciences, Zhejiang Chinese Medical University, Hangzhou, Zhejiang, China; 3 Zhejiang-Hong Kong Joint Laboratory of Liver and Spleen Simultaneous Treatment in Traditional Chinese Medicine, Hangzhou, Zhejiang, China

**Keywords:** Rhizoma alismatis, phytochemistry, pharmacology, toxicology, processing

## Abstract

**Background:**

*Rhizoma alismatis* is a traditional Chinese medicine with a long history. It is an important part of many prescriptions and is often used to treat water metabolism-related diseases in clinical practice. At present, there are 12 species of *R. alismatis*, of which only *Alisma plantago-aquatica* L. and *A. plantago-aquatica subsp. orientale* (Sam.) Sam are used as traditional Chinese medicine.

**Objective:**

Based on the scientific literature, this paper aims to provide comprehensive and up-to-date information on the botany, traditional uses, phytochemistry, pharmacology, toxicology and processing methods of *R. alismatis*. Furthermore, it seeks to analyze current research findings to establish a new foundation and direction for future studies.

**Methods:**

Multidisciplinary research domains including botanical identification, ethnopharmacological applications, phytochemical constituents, pharmacological activities, toxicological profiles, and processing techniques, drawing upon extensive data retrieved from PubMed, Web of science, CNKI, and other authoritative databases.

**Results:**

Traditional Chinese medicine believes that *R. alismatis* has the effects of promoting water and dampness and venting heat. Modern studies have found that its extracts and isolated compounds have diuretic, liver protection, lowering blood pressure, lowering blood glucose, anti-cancer, anti-inflammatory and antioxidant activities. The toxicity of *R. alismatis* has long been a controversial topic, and it is generally held that no obvious adverse reactions occur within the prescribed dosage range.

**Conclusion:**

Modern studies partially explains the traditional concept of *R. alismatis’* functions and the corresponding pharmacodynamic material basis. It is necessary to further study the network relationship between traditional usage, modern pharmacology and toxicity, and standardize the cultivation, processing and circulation system of *R. alismatis*.

## 1 Introduction

*Rhizoma alismatis* (known as “Zexie” in Chinese), a perennial aquatic herb of the genus *Alisma* (Alismataceae), predominantly inhabits temperate and subtropical marshlands across the Northern Hemisphere (Commission, 2020). Among 12 globally recognized *Alisma* species, six are endemic to China, with *Alisma plantago-aquatica* L. and *A. plantago-aquatica subsp. orientale* (Sam.) Sam holding particular medicinal significance. These species were first documented in the Book of Songs·Wei Feng (ca. 11th-7th century BCE) (Shu et al., 2016) and subsequently classified as a superior-grade (shang pin) botanical drug in the Shennong’s Herbal Classic (ca. 200–300 CE) for their dampness-resolving, diuretic, and kidney-tonifying properties (Anonymous, 2022). In clinical practice, it is often used as a sovereign drug, and compatible with different TCMs for the treatment of numerous diseases.

*Rhizoma alismatis* encompasses numerous chemical constituents, of which 262 metabolites have been identified thus far. According to the structure, these metabolites can be primarily classified into seven categories: terpenoids, sugars, nitrogen-containing compounds, phenylpropanoids, flavonoids, steroids and phenolic acids. Among them, terpenoids are particularly abundant, including triterpenoids, sesquiterpenes and diterpenes. Triterpenes are considered to be the main active ingredients of *R. alismatis*. The structure of triterpenoids is mostly prototerpene tetracyclic triterpenoids, including alisol A, alisol B, alisol C, and alisol B 23-acetate etc. (Xiao et al., 2024). Modern pharmacology has demonstrated its diuretic (Huang et al., 2016; Li R. et al., 2020; Wang et al., 2008), kidney stone protective, lipid-lowering (Miao et al., 2016; Yan P. et al., 2022), hypoglycemic (Lau et al., 2008), liver-protecting (Hur et al., 2007; Sun et al., 2020) and antibacterial (Jin et al., 2012) and anti-tumor (Li L. et al., 2020; Liu et al., 2019; Lou et al., 2019; Wu et al., 2023; Xu et al., 2015; Zhang et al., 2016) effects.

In recent years, the toxicity of Chinese botanical drugs has been paid more and more attention. Although TCM theory posits that *R. alismatis* is non-toxic and may confer tonic benefits with prolonged use (Anonymous, 2022; Jing, 1957), modern pharmacological studies have elucidated its dose-dependent toxicological characteristics, including dermal toxicity (Cash crop team of Guangdong Agriculture, 1970), gastrointestinal reactions and systemic toxicity (Chen and Zhou, 1998). However, most of the above situations occur when the dose is much higher than the conventional dose and the high dose is rich in the extract of *R. alismatis* terpenoids. Therefore, it is necessary to conduct in-depth and systematic research to objectively and comprehensively evaluate the clinical toxicity and adverse reactions of *R. alismatis*.

Pharmaceutical processing (Paozhi), a critical practice in TCM, modulates the therapeutic profile of medicinal materials through targeted physicochemical modifications (Qiu, 2003). *Rhizoma alismatis* undergoes multiple processing techniques, including: salt-water immersion, wine stir-frying (Zhong et al., 2005), salt stir-frying, bran stir-frying, soil stir-frying (Wang, 1993). Contemporary practice prioritizes bran-frying and salt-processing due to their distinct pharmacodynamic outcomes. Bran-fried *R. alismatis* demonstrates enhanced dampness-resolving properties with spleen-tonifying and turbidity-reducing capacities, whereas salt-processed variants exhibit yin-nourishing effects coupled with heat-clearing and diuretic actions (Yan et al., 2020; Zhong et al., 2005). Mechanistic studies attribute these functional shifts to processing-induced triterpenoid profile alterations, particularly the content of protostane-type triterpenes (Yan et al., 2023).

Drawing on data and feedback from clinical practice, we have identified key challenges in the application of *R. alismatis*. These challenges stem from issues in its cultivation, material quality, processing and preparation, contradictions between traditional uses and modern pharmacological research, as well as ongoing debates concerning its toxicity. To address these gaps, we have undertaken a comprehensive effort by compiling extensive literature, conducting a systematic analysis, and incorporating the latest pharmacological evidence. This work aims to provide a comprehensive and up-to-date resource to support the effective and safe clinical use of *R. alismatis*.

## 2 Research methodology

This study conducted a systematic review of *R. alismatis* research spanning 5 decades (1970–2024). A comprehensive analysis was performed on publications addressing botanical characteristics, ethnomedicinal applications, phytochemical profiles (including secondary metabolites), pharmacological properties, toxicological assessments, and processing methodologies. The search protocol utilized multidisciplinary databases (Scopus, Web of science, PubMed, ACS Publications) alongside specialized platforms (CNKI, WanFang) and broad-coverage engines.

Search strategies incorporated controlled vocabulary and natural language terms: 1. Base term: *R. alismatis*, 2. Thematic expansions: chemical composition, traditional medicine, toxicity, pharmacological activity. 3. Functional modifiers: diuretic, anti-inflammatory, antioxidant, hypoglycemic, hepatoprotective, antibacterial, anti-tumor. 4. Process terminology: processing. The search results yielded a substantial number of articles, which were then evaluated for relevance based on their titles and abstracts, utilizing Boolean operators (AND, OR) to optimize the search strategy. We also meticulously reviewed the reference lists of these papers to identify any additional articles pertinent to this literature review. Literature inclusion criteria: Studies on *R. alismatis*, relevance to the review scope, and coverage of botany, ethnomedicine, pharmacology, toxicology, chemistry, and processing methods. The exclusion criteria were articles without full texts and irrelevant articles that fell outside the scope of this review.

Unlike existing reviews, this study critically addresses research limitations and emphasizes the necessity to: analyze historical changes in the functional uses of Alisma in medicine; integrate reported high-abundance active metabolites; clarify active metabolites and their key molecular targets and mechanisms of action; summarize key aspects of traditional medicinal applications and their alignment or conflict with modern pharmacology; and consolidate toxicological data by clearly distinguishing extract types, administration doses, and treatment durations. Furthermore, this work proposes strategies for enhancing efficacy and reducing toxicity, discusses underlying mechanisms, and aims to promoting the effective clinical application of *R. alismatis*. Key unresolved issues include the standardized cultivation and processing of *R. alismatis*, in-depth investigation of specific pharmacological activities, and the interactive effects—both beneficial and toxic—of Alisma in combination with other drugs. Accordingly, this study synthesizes current knowledge on the chemical composition, pharmacological activities, and toxicological data of Alisma, identifies persistent challenges, and proposes strategic research directions to advance its therapeutic application.

## 3 Study selection

**FIGURE 1:**
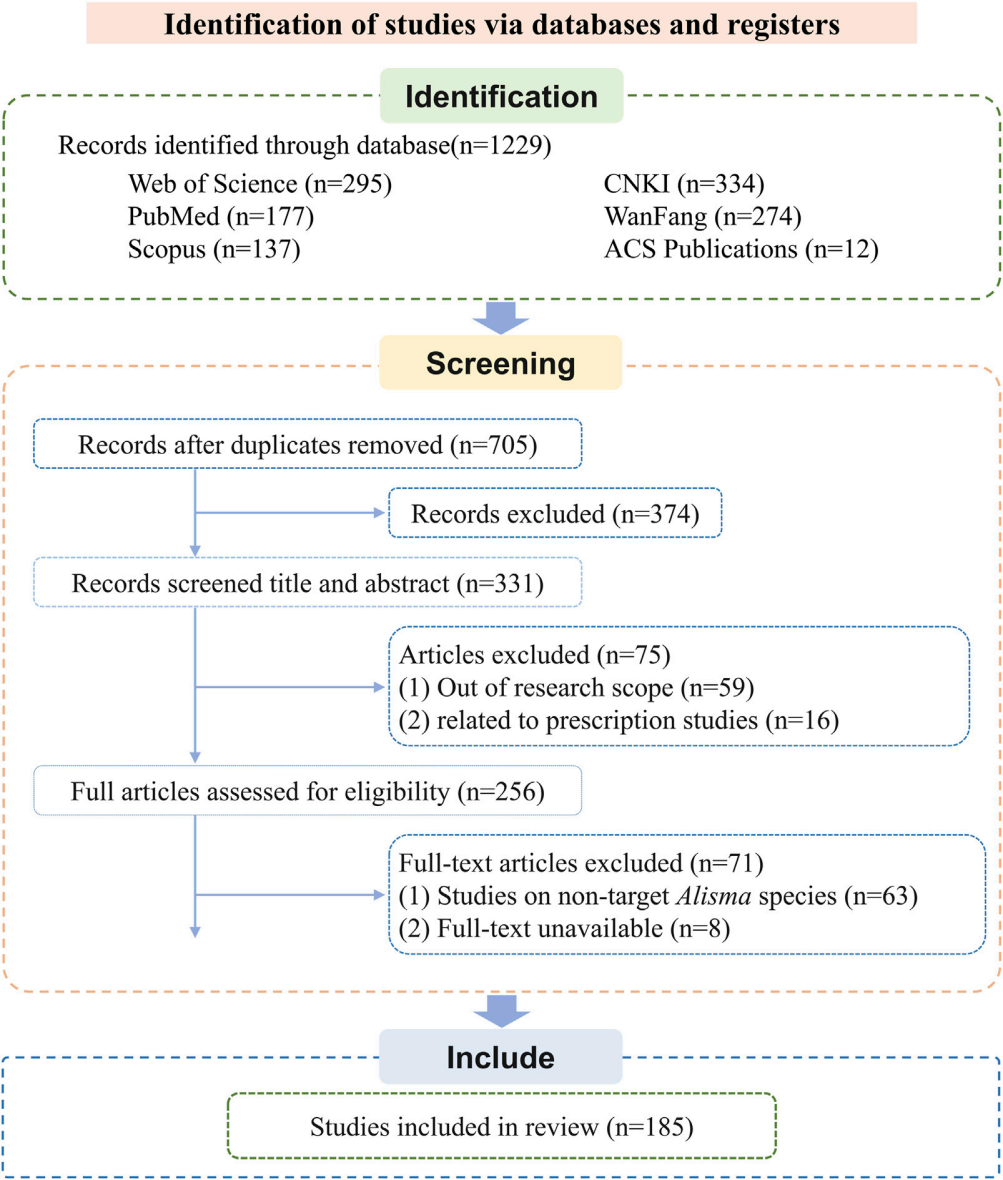
Flow diagram for the process of included studies identification.

A systematic literature search was performed across six major scientific databases, resulting in the identification of 1,229 records. The screening procedure adhered to an adapted PRISMA flowchart framework (Page et al., 2021), with specific modifications as illustrated in Figure 1. The databases searched were as follows: Web of Science (n = 295), PubMed (n = 177), Scopus (n = 137), ACS Publications (n = 12), CNKI (n = 334), and WanFang Data (n = 274). Following duplicate removal using EndNote and subsequent manual verification, 705 articles remained for preliminary assessment. Through title and abstract screening, 374 records were excluded. The remaining 331 publications underwent full-text review. Of these, 59 were excluded for falling outside the research scope, 16 pertained to prescription studies, 63 involved non-target Alisma species, and 8 were excluded due to unavailability of the full text. After this rigorous selection process, 185 studies satisfied all predefined inclusion criteria and were included in the qualitative synthesis.

## 4 Results

### 4.1 The source and origin of *Rhizoma alismatis*

**FIGURE 2:**
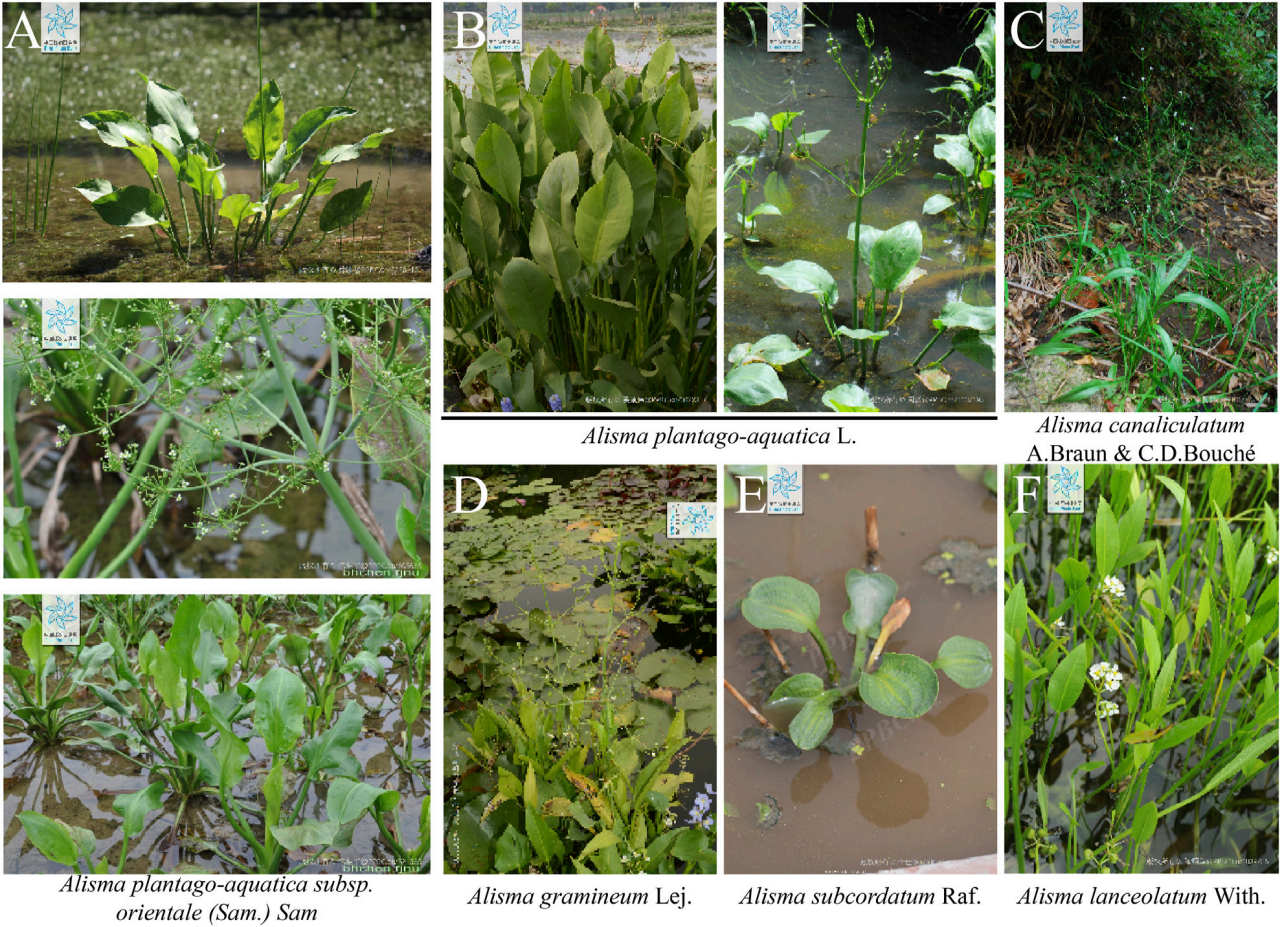
Morphological characteristics of several major *Rhizoma alismatis* species: **(A)** *Alisma plantago-aquatica subsp. orientale* (Sam.) Sam; **(B)** *Alisma plantago-aquatica* L.; **(C)** *Alisma canaliculatum* A.Braun and C.D. Bouché; **(D)** *Alisma gramineum* Lej.; **(E)** *Alisma subcordatum* Raf. **(F)** *Alisma lanceolatum* With (Cite from PPBC https://ppbc.iplant.cn/).

According to the Flora Reipublicae Popularis Sinicae, the Plant List and the Plant Science Data Center, there are 12 species of the genus *Alisma*, which are mainly distributed in temperate and subtropical regions of the northern hemisphere. They are *Alisma plantago-aquatica* L.*, A. plantago-aquatica subsp. orientale* (Sam.) Sam*, Alisma canaliculatum* A. Braun & C.D. Bouché*, Alisma lanceolatum* With., *Alisma gramineum* Lej*., Alisma subcordatum* Raf.*, Alisma annuum* Lojac.*, Alisma difformifolium* Steud.*, Alisma intermedium* Griff. ex Voigt, *Alisma taeniifolium* Steud.*, Alisma triviale* Pursh, *Alisma wahlenbergii* (Holmb.) Juz. (Figure 2). Among them, only *A. plantago-aquatica* L. and *A. plantago-aquatica subsp. orientale* (Sam.) Sam have been included in the Chinese Pharmacopoeia, with a documented medicinal history of 2,000 years, and have been proven to possess significant therapeutic value (Commission, 2020).

*Rhizoma alismatis*, a classical herbal medicine in TCM, maintains significant commercial importance in global herbal markets. Annual domestic consumption in China reaches approximately 8,000 metric tons, with an additional 1,000 metric tons exported to Japan, South Korea, and Southeast Asian countries (Liu et al., 2020). Morphological distinctions among these varieties are noteworthy. It can be divided into three categories in China. Jian *R. alismatis* (Fujian, Jiangxi) typically presents round-to-oval morphology with yellowish-white coloration, exhibiting irregular transverse annular grooves and lacking basal tubercular protrusions. Its compact texture reveals substantial yellowish-white starchy parenchyma upon cross-section. In contrast, Chuan *R. alismatis* (Sichuan) displays oval morphology with yellowish-brown periderm, regular concentric striations, and characteristic basal tubercles, demonstrating lighter mass with reduced starch content. Guang *R. alismatis* (Guangxi) shares oval morphology but features discontinuous concentric markings with multiple tubercular protrusions (Zhao et al., 2024). Quality assessment parameters prioritize large size, firm texture, yellowish-white coloration, and high starch content as superior characteristics, whereas smaller dimensions, rough epidermis, fragmentation, and scorched-yellow discoloration indicate inferior quality. Although Jian *R. alismatis* is traditionally regarded as the premium variety, its limited production (representing <10% of national output) contrasts sharply with Chuan *R. alismatis*’s market dominance (>90% total production). Guang *R. alismatis*’s comparatively inferior quality places it at a competitive disadvantage (Gu et al., 2019). Phylogenetic investigations using ITS2 sequencing have revealed significant taxonomic discrepancies in commercial samples. Song et al. (Ma et al., 2015) demonstrated that 93% of market specimens labeled as *Alisma plantago-aquatica subsp. orientale* (Sam.) Sam. actually corresponded to *A. plantago-aquatica* L., with authentic *Alisma orientale* representing only 7% of sampled materials. Chemotaxonomic analysis further indicates that Chuan *R. alismatis* aligns with *A. plantago-aquatica* L. in protostane-type tetracyclic triterpenoid profiles, while Jian *R. alismatis* corresponds to *A. orientale* characteristics (Liu, 2019). However, current cultivation patterns demonstrate interspecific hybridization across production regions, with multiple varieties cultivated within single geographic zones exhibiting phenotypic convergence (Tian et al., 2020). The above phenomenon indicates that the influence of planting environment on the quality of *R. alismatis* may be greater than that of the varieties of *R. alismatis*, which also explains to a certain extent why the TCM *R. alismatis* is mainly distinguished by the place of origin in China. The above situation reveals that there are serious problems in the process of cultivation, planting management and circulation of *R. alismatis*. It is urgent to establish a scientific and standardized management system for the cultivation and circulation.

### 4.2 Traditional Chinese medicine *Rhizoma alismatis*, classical prescription and related proprietary Chinese medicine products

#### 4.2.1 *Rhizoma alismatis* Chinese herbal properties

According to the *Pharmacopoeia of the People’s Republic of China* (2020 edition), *R. alismatis* documented pharmacological actions include diuresis promotion, dampness elimination, turbidity resolution, and hypolipidemic effects (Commission, 2020). However, historical textual analysis reveals significant evolution in its perceived medicinal properties across dynastic periods (Niu et al., 2024). Early pharmacopeias like “Sheng Nong’s herbal classic” (Han Dynasty) and “Famous Doctor Bielu” (Northern and Southern Dynasties), and “Xinxiu Bencao”, “Yaoxing Lun” and “Rihuazi Bencao” (Tang Dynasty) emphasized its diuresis, viscera-nourishing properties, yin-activating and potential skin protection capacity. Clinical applications focused on managing tinnitus, dystocia, hematuria, and infertility through diarrhea inhibition and essence stabilization.

These points were inherited in “Compendium of Materia Medica” and “Huiyan of Materia Medica”, but divergent perspectives also emerged, as recorded in “Bencao Yanyi” and “Bencao Yueyan”. While maintaining consensus on diuretic-dampness removal efficacy, debates arose regarding its tonifying effects. Some texts paradoxically described nourishing of five viscera organs and yin deficiency supplementation. Then to the Qing Dynasty and modern, the effect of *R. alismatis* on diuresis was discussed in depth, purging fire of liver and kidney meridians, expelling the water of bladder and triple energizer, diuresis-removing dampness, reducing turbidity and lipid in “herbal reading”, “herbal justice”, “ten lectures on medication experience” and the 2020 edition of “Pharmacopoeia of the People’s Republic of China”. It is aimed at adverse urination, edema and fullness, diarrhea and less urine, phlegm and vertigo, hot and astringent pain, and hyperlipidemia in clinically, but tonic effect of long-term use are no longer mentioned. The shift in people’s understanding of the functions of *R. alismatis* is a thought-provoking issue. Factors such as environmental changes, artificial cultivation, and sufficient food supplies have all influenced perceptions of this botanical drug. If we can explore the underlying reasons behind this transformation, it would be a meaningful endeavor to revive its tonic properties and reemphasize its role in nourishment.

#### 4.2.2 Commonly used classical prescriptions of *Rhizoma alismatis* and its related Chinese patent medicines

*Rhizoma alismatis* has been used in many classic prescriptions, among which Zexie Decoction and Wuling Powder are renowned. Zexie Decoction originates from the “Synopsis of the Golden Chamber”. It consists of two types of medicine: *R. alismatis* (Zexie) and *Atractylodes macrocephala* Koidz. (Baizhu) (Zhang, 2018). This prescription is a classic prescription for clinical treatment of phlegm and fluid retention syndrome. *Rhizoma alismatis* is a sovereign drug, diuresis and dampness, *Rhizoma atractylodis macrocephalae* is a minister drug, invigorating spleen and replenishing qi. Wuling Powder, composed of five medicines: *Poria cocos* (Schw.) Wolf (Fuling), *Polyporus umbellatus* (Pers.) Fries (Zhuling), bran-fried *A. macrocephala* Koidz. (Fu chao Baizhu), *R. alismatis* (Zexie), and *Cinnamomum cassia* (L.) J. Presl (Guizhi), can treat the dehydration and water storage of diabetes. The water metabolism of the human body involves multiple organs such as lung, spleen, stomach, kidney, bladder and triple energizer. Any dysfunction of the organs involved in the regulation of water can cause the syndrome of water-dampness stagnation.

The modernization of pharmaceutical technologies has driven the transformation of traditional Chinese medicinal materials into standardized dosage forms, including granule preparations, capsules, pills, and tablets, for commercial distribution. *Rhizoma alismatis*-containing patent medicines occupy a substantial market share, with over 90 formulations officially documented in the Pharmacopoeia of the People’s Republic of China (2020 edition) (Commission, 2020). Longqing Tablet possesses the functions of clearing heat and detoxifying, cooling blood, removing dampness and promoting diuresis. It is clinically used for the treatment of urinary tract infection, benign prostatic hyperplasia, acute and chronic prostatitis, and other heat stranguria resulting from dampness-heat in the lower jiao (Chen et al., 2016). Xuezhiling Tablet is composed of *R. alismatis* (Zexie)*, Cassia obtusifolia* L. (Juemingzi)*, Crataegus pinnatifida* Bunge (Shanzha) and *Polygonum multiflorum* Thunb. (Zhiheshouwu). *Rhizoma alismatis* is used as the sovereign drug for its diuresis, turbidity-dissolving and lipid-lowering effect, and is clinically used for the treatment of hyperlipidemia caused by phlegm block. Wuling Capsule is mainly employed for warming yang, transforming qi, removing dampness and promoting water circulation (Liu and Wang, 2023; Ma et al., 2024; Mou et al., 2022; Yang et al., 2015) (Supplementary Table S1).

### 4.3 Chemical composition of *Rhizoma alismatis*

**FIGURE 3:**
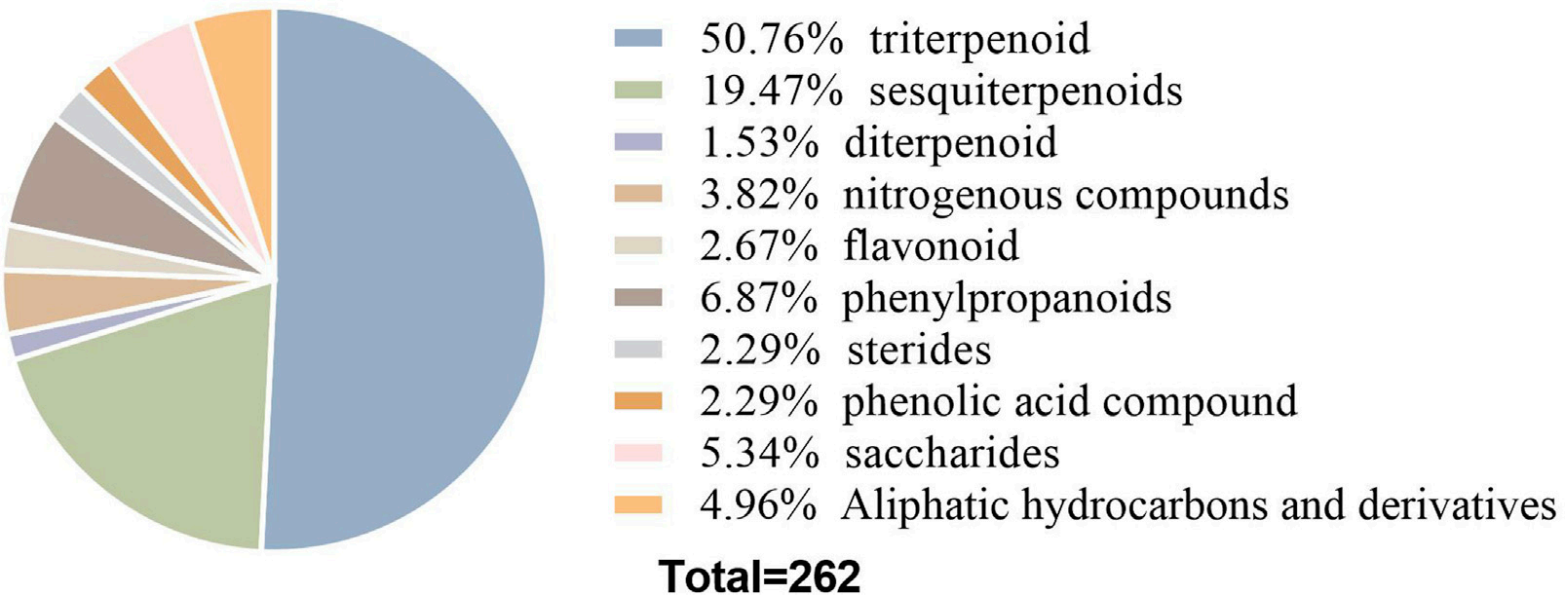
Sector diagram of the proportion of *Rhizoma alismatis* monomer types.

Current phytochemical investigations have identified over 260 compounds in *R. alismatis*, encompassing carbohydrates (3 polysaccharides, 6 oligosaccharides, 5 monosaccharides), terpenoids (133 triterpenoids, 51 sesquiterpenes, 4 diterpenes), nitrogenous compounds (10), phenylpropanoids (18), flavonoids (7), steroids (6), phenolic acids (6), and 13 aliphatic hydrocarbon derivatives (Liu et al., 2020; Dai et al., 2023). Terpenoids dominate the chemical profile, representing 70.6% of total constituents (Figure 3), with triterpenoids and sesquiterpenes being particularly abundant.

#### 4.3.1 Carbohydrate

At present, 14 kinds of carbohydrates have been reported in *Rhizoma alismatis,* 3 kinds of polysaccharides: alisman SI, alisman PII, alisman PIIIF; six kinds of oligosaccharides: manninotriose, verbascotetraose, verbascose, raffinose, stachyose, sucrose; five monosaccharides: β-D-fructofuranose, 5-hydroxymethylfurfuraldehyde, α-D-fructofuranose, ethylα-D- fructofuranoside, ethylβ-D-fructofuranoside, nine of which are fructose-derived carbohydrates (Zhang et al., 2009).

Alisman SI is a glucan composed only of D-glucose. Methylation analysis, nuclear magnetic resonance and enzymatic degradation studies have shown that alisman SI has a highly branched glucan type structure, composed mainly of α-1, 4-linked D-glucopyranosyl residues, and partially with α-1, 6-linked units. It has both 3,4 - and 4, 6-branch points (Shimizu et al., 1994). Alisman PII is an acidic polysaccharide composed of L-arabinose: D-galactose: D-glucuronic acid in a 4:9:2 M ratio. Its core structural features include a bony chain composed of β-1, 3-linked D-galactose units (Tomoda et al., 1994). Huang Suoyi et al. (Li et al., 2012) extracted *R. alismatis* polysaccharide by water extraction and alcohol precipitation method, and determined that the polysaccharide content of *R. alismatis* was 5.783% by phenol-sulfuric acid method.

#### 4.3.2 Terpenoids

**FIGURE 4:**
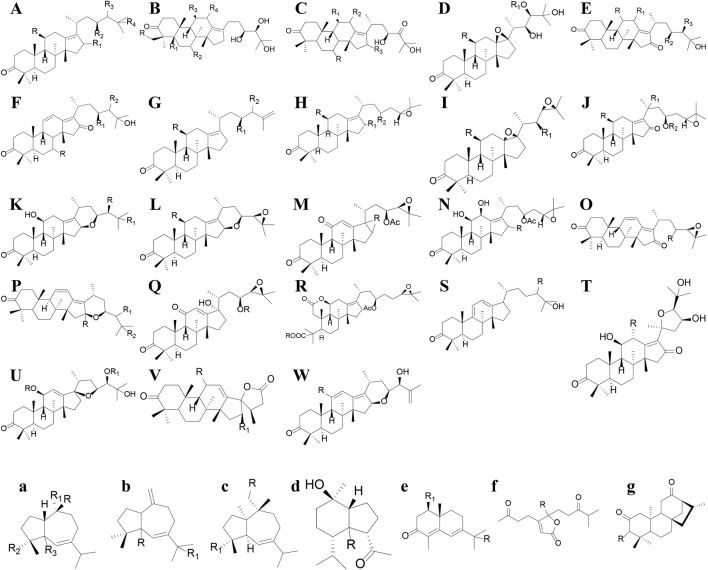
The nucleus of the triterpenes and Sesquiterpenes in *Rhizoma alismatis*. The nucleus of the triterpenes in *Rhizoma alismatis* **(A–W)**. The nucleus of the sesquiterpenes in *R. alismatis* **(a–g)**.

**FIGURE 5:**
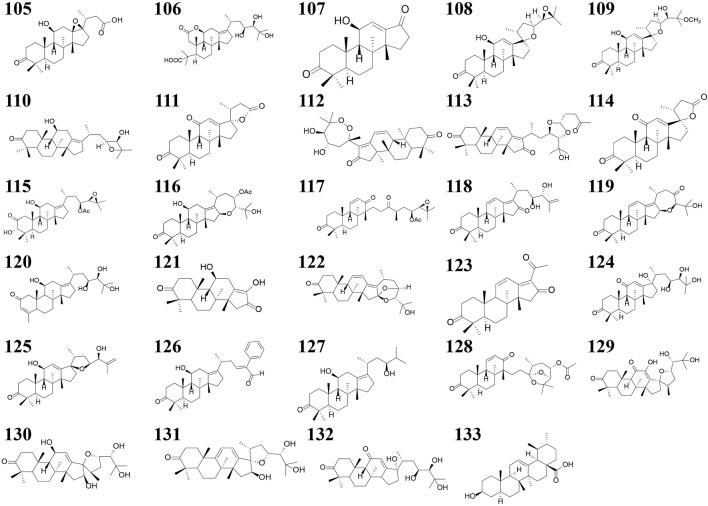
The structures of the triterpenes in *Rhizoma alismatis*.

**FIGURE 6:**
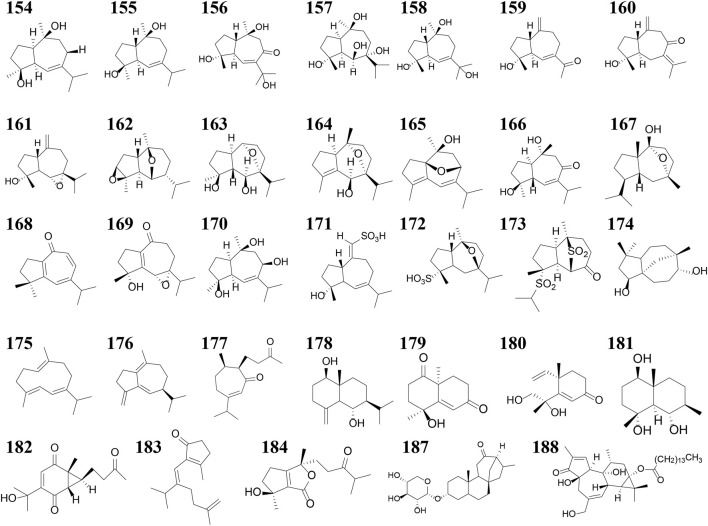
The nucleus of the Sesquiterpenes in *Rhizoma alismatis*.

Terpenoids can also be divided into triterpenes, sesquiterpenes and diterpenoids (see Supplementary Table S2; Figures 4–6 for details). Among these triterpenes, alisol A, alisol B and its acetate as well as alisol C 23-acetate are initially isolated from *R. alismatis* in 1970 (Murata et al., 1970). These triterpenes are all derived from the alisol B 23- acetate which is highly content in the fresh plants (Peng, 2006). Most of their chemical skeletons are prototerpene tetracyclic triterpenoids represented by alisol A-X and its derivatives and alismanol A-Q. Only four diterpenoids were detected, namely oriediterpenone, oriediterpenol, oriediterpenoside and 12-deoxyphorbol-13a-pentadecanoate.

#### 4.3.3 Other types of compounds

The remaining compounds in *R. alismatis* include nitrogen compounds, phenylpropanoid compounds, steroids, flavonoids, phenolic acids, aliphatic hydrocarbons and derivatives (see Supplementary Table S3; Supplementary Figure S1), accounting for about 30% of the identified compounds. Among them, the nitrogen compounds were mainly bases, nucleosides and indole components, and the steroids were mainly β-sitosterol, carotene and its derivatives.

### 4.4 Pharmacological activities of *Rhizoma alismatis*

**FIGURE 7:**
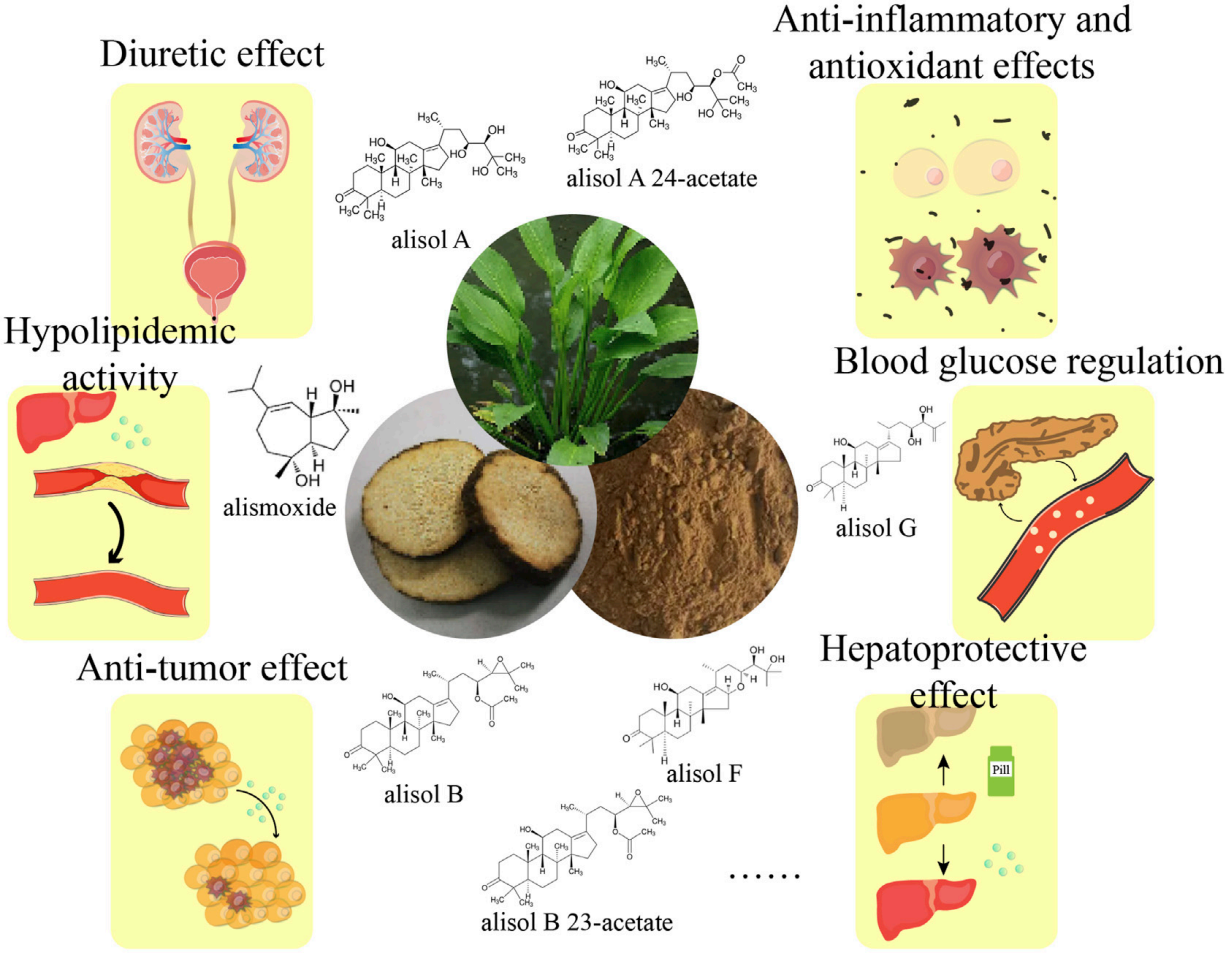
Pharmacodynamic of *Rhizoma alismatis*.

*Rhizoma alismatis* is rich in chemical substances. Modern pharmacological studies have shown that it also has a variety of biological activities, such as diuretic inhibition of kidney stone formation, anti-inflammatory and antioxidant effects, blood lipid regulation, hypoglycemic, liver protection and antibacterial, anti-tumor effects (Figure 7; Table 1).

**TABLE 1:** The bioactivities of extracts and compounds from *Rhizoma alismatis*.

No.	Pharmacological activities	Extract/compound	Test living system	Routes of administration/Dose	Duration	Refs
1	diuretic activity	80% ethanol extract of *Rhizoma alismatis*	Female SD rats	0.9 and 4.0 g/kg/d^a^	3 days	Li R. et al. (2020)
	diuretic activity	total triterpene extract (TTE) and triterpene component compatibility	Male SD rats	5, 20 and 40 mg/kg/d^a^	Single	Zhang et al. (2017)
	diuretic activity	Water extract, petroleum ether extract, ethyl acetate extract, n-butanol extract of *Rhizoma alismatis*	Male SD rats	2 g/kg/d^a^	—	Huang et al. (2016)
	diuretic activity	*Rhizoma alismatis* 80% ethanol extract	Male SD rats	20 mg/kg/d^a^	—	Wang et al. (2008)
	diuretic activity	*Rhizoma alismatis* water extract	Male SD rats	20 mg/kg/d^a^	—	Wang et al. (2008)
	diuretic activity	24-acetylalisberol A	Male SD rats	20 mg/kg/d^a^	—	Wang et al. (2008)
	dual diuretic effect	Petroleum ether fraction (Fr. PE), ethyl acetate fraction (Fr.EA), n-butanol fraction (Fr.NB) and remaining fractions of *Rhizoma alismatis* 95% ethanol extract (Fr.ME)	Male SD rats	Fr. PE (12.5, 25, 50 mg/kg/d)^a^ Fr. EA (100, 200, 400, 600, 800 mg/kg/d)^a^ Fr. NB (12.5, 25, 50, 75, 100 mg/kg/d)^a^ Fr. ME (12.5, 25, 50 mg/kg/d)^a^	—	Tian et al. (2014)
	dual diuretic effect	*Rhizoma alismatis* 95% ethanol extract (EE) and water extract (WE)	Male SD rats	EE (2.5, 5, 10 mg/kg/d)^a^ WE (75, 150, 300 mg/kg/d)^a^	—	Feng et al. (2014)
	Protective effect on kidney stones	*Rhizoma alismatis* polysaccharide	HK-2 cell line	0.02–0.1 mg/mL^b^	24 h	Wang et al. (2024)
	Inhibition of kidney stone formation and protection	*Rhizoma alismatis* polysaccharide	HK-2 cell line	0.04–0.3 mg/mL^b^	6 h	Cheng and Ouyang (2023)
2	Anti-inflammatory	Fuling-Zexie (FZ) formula (Fuling, Zexie, Gegen, Cangzhu)	Male KM mice	7.5, 15 and 30 g/kg/d^a^	7 days	Lu et al. (2024)
	Anti-inflammatory	Fuling-Zexie (FZ) formula (Fuling, Zexie, Baizhu, Guizhi, Shenggancao, Shengjiang)	asymptomatic hyperuricemia patient	/^c^	12 week	Yan et al. (2022)
	Anti-inflammatory	*Rhizoma alismatis* 80% ethanol extract	RAW 264.7 cells	1, 5, 10 µg/mL^b^	16 h	Han et al. (2013)
	Anti-inflammatory and lipid-lowering effects	Alisol A	C57BL/6 mice	25, 100 mg/kg/d^a^	12 week Twice 1 days	Wang et al. (2020)
	Anti-inflammatory and lipid-lowering effects	Alisol A	HepG2	1, 5, 10, 20, 30, 40, 60,80,100 μM ^b^	24 h	Wang et al. (2020)
	Anti-inflammatory	Plantain A	Male Wistar rats	50 mg/kg/d^a^	3 week	Huang et al. (2017)
	Anti-inflammatory	Alismol	BV2 cell line	50, 100 μM^b^	24 h	Liu (2012)
	Anti-inflammatory	Alismol	C57BL/6 mice	0.22, 2.2 mg/kg/d^d^	—	Kim et al. (2023)
	Anti-inflammatory	Alismol	RAW 264.7 cells	0.01 μM^b^	16 h	Kim et al. (2023)
	Anti-inflammatory	Alisol B 23-acetate	Male C57BL/6 mice	10, 20, 40 mg/kg^a^	30 days	Wang et al. (2019)
	Anti-inflammatory	Alisol F	RAW 264.7 cells	3.7, 11, 33 μM^b^	24 h	Ma et al. (2016)
	Anti-inflammatory	25-anhydroalisol F	RAW 264.7 cells	3.7, 11, 33 μM^b^	24 h	Ma et al. (2016)
	Anti-inflammatory	Alisols A	LPS-Stimulated Mouse Macrophages	1, 3, 10, 30, 100 μM^b^	20 h	Matsuda et al. (1999)
	Anti-inflammatory	Alisols A monoacetate	LPS-Stimulated Mouse Macrophages	1, 3, 10, 30, 100 μM^b^	20 h	Matsuda et al. (1999)
	Anti-inflammatory	Alisols B	LPS-Stimulated Mouse Macrophages	1, 3, 10, 30, 100 μM^b^	20 h	Matsuda et al. (1999)
	Anti-inflammatory	Alisols B monoacetate	LPS-Stimulated Mouse Macrophages	1, 3, 10, 30, 100 μM^b^	20 h	Matsuda et al. (1999)
	Anti-inflammatory	Alisols E	LPS-Stimulated Mouse Macrophages	1, 3, 10, 30, 100 μM^b^	20 h	Matsuda et al. (1999)
	Anti-inflammatory	Alisols G	LPS-Stimulated Mouse Macrophages	1, 3, 10, 30, 100 μM^b^	20 h	Matsuda et al. (1999)
	Anti-inflammatory	Alisols K-23-acetate	LPS-Stimulated Mouse Macrophages	1, 3, 10, 30, 100 μM^b^	20 h	Matsuda et al. (1999)
	Anti-inflammatory	Alisols N-23-acetate	LPS-Stimulated Mouse Macrophages	1, 3, 10, 30, 100 μM^b^	20 h	Matsuda et al. (1999)
	Anti-inflammatory	Alisols 11-deoxyalisol B	LPS-Stimulated Mouse Macrophages	1, 3, 10, 30, 100 μM^b^	20 h	Matsuda et al. (1999)
	Antioxidant	Extract of Zexie Decoction	*Caenorhabditis elegans* in high glucose nematode growth media	50, 100, 200, 300, 400, 500 mg/mL^e^	—	Shi et al. (2023)
	Antioxidant	*Rhizoma alismatis* 80% ethanol extract	Male C57BL/6 mice	0.3, 1.2 g/kg/d^a^	14 d	Han et al. (2013)
	Antioxidant	*Rhizoma alismatis* water extract	HepG2 cell	100 μg/mL^b^	21 h	Han et al. (2012)
	Antioxidant	Syringaresinol	RAW 264.7 cell	55.28 μM^f^	—	Zhao et al. (2017)
3	Regulation of blood lipids	Extract of Zexie Decoction	Male C57BL/6 mice	1.3, 2.6, 5.2 g/kg/d^a^	10 w	Xie et al. (2022)
	Reduced lipid accumulation	Extract of Zexie Decoction	HL7702 cells	100, 200, 300 μg/mL^b^	18 h	Xie et al. (2022)
	Regulation of blood lipids	*Rhizoma alismatis* acetonitrile extract (1:10)	Male KM mice	20 g/kg/d^a^	4 week	Yan P. et al. (2022)
	Regulation of blood lipids	Ethanol extract of the rhizome of Alisma orientale (AOE)	OP9 Cells	10, 20, 40 μg/mL^b^	24–96 h	Park et al. (2014)
	Regulation of blood lipids	Extract of zexie decoction	Male KM mice	3.4, 8.6, 12.0 g/kg/d^a^	28 days	Ju et al. (2023)
	Regulation of blood lipids	Extract of zexie decoction	Male SD rats	80 g/kg/d^a^	6 week	Miao et al. (2016)
	Regulation of blood lipids	*Rhizoma alismatis* water extract	Male C57BL/6 mice	3.8 g/kg/d^a^	8 week	Liu et al. (2023)
	Regulation of blood lipids	Alisol A,alisol B,23-acetyl alisol C (23C)	Male ICR mice	0.64, 1.28, 2.56 mg/kg/d^a^	3 week	Xu et al. (2020)
	Regulation of blood lipids	alisol G	HepG2 cells	1, 10, 20 μM^b^	24 h	Zhang et al. (2022)
4	Regulation of blood glucose	*Rhizoma alismatis* water extract	H4IIE, Hs68, 3T3-L1 cells	0.04, 0.2, 1.0 mg/mL^b^	5 h	Lau et al. (2008)
	Regulation of blood glucose	Rhizoma alismatis ethanol extract	Male KM mice	10, 20 mg/kg/d^a^	7 days	Yang et al. (2004)
	Regulation of blood glucose	Rhizoma alismatis ethanol extract	3T3-L1 cells	25, 50,100 μg/mL^b^	48 h	Li and Qu (2012)
	Regulation of blood glucose	Rhizoma alismatis ethanol extract	Male SD rats	0.5 g/kg/d^a^	—	Li and Qu (2012)
	Regulation of blood glucose	*Rhizoma alismatis* polysaccharide	Male SD rats	100, 200, 400 μg/mL/d^a^	5 week	M. Zhang et al. (2018)
	Renoprotective effects of hyperglycemia	*Rhizoma alismatis* polysaccharide	Male SD rats	100, 200, 400 μg/mL/d^a^	6 week	Dong et al. (2023)
	Regulation of blood glucose	Alismoxide	C57BL/6	5,10, 20 μg/mL/d^a^	3 week	Zhang et al. (2019)
	Regulation of blood glucose	Alisol A	HepG2 cells	1, 5, 10 μM^b^	24 h	Lin et al. (2023)
5	Hepatoprotective	Alisma shugan dectomion	Male Wistar rats	50 mg/kg/d^d^	45 d	Sun et al. (2020)
	Hepatoprotective	Extract of liuwei dihuang pill	Male BALB/c mice	0.8, 1.6, 3.2 g/kg/d^a^	7 days	Huang et al. (2019)
	Hepatoprotective	*Rhizoma alismatis* methanol extract	Male SD rats	250, 500 mg/kg/d^a^	1 week	Hur et al. (2007)
	Hepatoprotective	Alisol B 23-acetate	Male C57BL/6 mice	10, 20, 40 mg/kg/d^a^	7 days	Meng et al. (2015)
	Hepatoprotective (NAFLD)	*Rhizoma alismatis* ethanol extract	HepG2 cells	1, 10, 50, 100, 300, 500, 1000 μg/mL^b^	16 h	Jeong et al. (2016)
	Hepatoprotective (NAFLD)	Methanol Extract of the Tuber of *Rhizoma alismatis* (MEAO)	HepG2 cells	10, 50, and 100 µg/mL^b^	16 h	Jang et al. (2015)
	Hepatoprotective (NAFLD)	*Alisma orientalis* methanolic extract (AOME)	Male SD rats	150,300 and 600 mg/kg^a^	6 week	Hong et al. (2006)
	Hepatoprotective (NAFLD)	Ziexie decoction	Male C57BL/6 mice	750, 1500 mg/kg/d^a^	12 week	Cao et al. (2022)
	Hepatoprotective (NAFLD)	Danshen zexie decoction	Male SPF rats	1.61, 2.32, 4.64 g/kg/d^a^	8 week	Biao et al. (2022)
	Hepatoprotective (NAFLD)	Ziexie decoction	male SD rats	100, 200, 400 mg/kg/d^a^	4 week	Zhang et al. (2022)
	Hepatoprotective (NAFLD)	Alisol F, 25-Anhydroalisol F	RAW 264.7 cells	0, 3.3, 11, 33, 100 μM^b^	24 h	Bi et al. (2017)
	Hepatoprotective (NAFLD)	Alisol F	Male C57BL/6 mice	20 mg/kg^a^	3 days	Bi et al. (2017)
6	Antitumor	Alisol A	MDA-MB-231 cells	10, 20, 40 μM^b^	24 h	Lou et al. (2019)
	Antitumor	Alisol B	HepG2, MDA-MB-231 and MCF-7 cells	HepG2 (16.28 μM)^f^ MDA-MB-231 (14.47 μM)^f^ MCF-7 (6.66 μM)^f^	—	Xu et al. (2015)
	Antitumor	Alisol B 23-acetate	HepG2, MDA-MB-231 and MCF-7 cells	HepG2 (18.01 μM)^f^ MDA-MB-231 (15.97 μM)^f^ MCF-7 (13.56 μM)^f^	—	Xu et al. (2015)
	Antitumor	Alisol B 23-acetate	SK-HEP-1 cells	10, 20, 30, 50, 70 μM^b^	24, 48, 72 h	Li L. et al. (2020)
	Antitumor	Alisol B 23-acetate	A549, BEAS-2B cells	6, 9 mM^b^	12, 24, 48 h	Liu et al. (2019)
	Antitumor	Alisol B 23-acetate	HEY, A2780 cells	6, 9, 12, 15, 18 mM^b^		Zhang et al. (2016)
	Antitumor	Alisol A 24-acetate	H460, MCF-7, PC-3	H460 (30.5 ± 1.5 μM)^f^ MCF-7 (43.1 ± 0.7 μM)^f^ PC-3 (28.5 ± 1.7 μM)^f^	72 h	Ma et al. (2016)
	Antitumor	Alisol B 23-acetate	H460, MCF-7, PC-3	H460 (56.7 ± 0 μM)^f^ MCF-7 (59.2 ± 4.2 μM)^f^ PC-3 (32.1 ± 1.7 μM)^f^	72 h	Ma et al. (2016)
	Antitumor	Alisol A	H460, MCF-7, PC-3	H460 (33.1 ± 1.4 μM)^f^ MCF-7 (43.6 ± 2.7 μM)^f^ PC-3 (27.3 ± 3.1 μM)^f^	72 h	Ma et al. (2016)
	Antitumor	Alisol B 23-acetate	HepG2	17.82 μM^f^	72 h	Xia et al. (2019)
	Antitumor	Alisol E 24-acetate	H460, MCF-7, PC-3	H460 (27.7 ± 0.6 μM)^f^ MCF-7 (32.9 ± 4.0 μM)^f^ PC-3 (26.3 ± 3.8 μM)^f^	72 h	Ma et al. (2016)
	Antitumor	Alisol G	H460, MCF-7, PC-3	H460 (11.5 ± 1.7 μM)^f^ MCF-7 (16.3 ± 0.9 μM)^f^ PC-3 (11.7 ± 1.7 μM)^f^	72 h	Ma et al. (2016)
	Antitumor	25-O-ethylalisol A	H460, MCF-7, PC-3	H460 (32.5 ± 2.3 μM)^f^ MCF-7 (57.7 ± 5.0 μM)^f^ PC-3 (33.4 ± 4.0 μM)^f^	72 h	Ma et al. (2016)
	Antitumor	11-deoxyalisol A	H460, MCF-7, PC-3	H460 (43.9 ± 0.3 μM)^f^ MCF-7 (61.9 ± 4.0 μM)^f^ PC-3 (40.7 ± 2.5 μM)^f^	72 h	Ma et al. (2016)
	Antitumor	10- hydroxy-7,10-epoxysalvialane	H460, MCF-7, PC-3	H460 (33.8 ± 2.0 μM)^f^ MCF-7 (52.7 ± 0.5 μM)^f^ PC-3 (66.6 ± 2.3 μM)^f^	72 h	Ma et al. (2016)
	Antitumor	1aH,5aH-guaia-6-ene-4b,10b-diol	H460, MCF-7, PC-3	H460 (56.7 ± 1.4 μM)^f^ MCF-7 (76.7 ± 1.4 μM)^f^ PC-3 (69.0 ± 1.9 μM)^f^	72 h	Ma et al. (2016)
	Antitumor	Ursolic acid	HL-60, BGC Bel-7402, Hela	Cytotoxicity ED_50_ (μg/mL) HL-60 (72.0 μg/mL)^f^ BGC (53.7 μg/mL)^f^ Bel-7402 (45.0 μg/mL)^f^ Hela (49.4 μg/mL)^f^	48 h	Ma et al. (2005)
	Antitumor	Ursolic acid	AGS, HepG2 HT-29, PC-3	AGS (<10 μM)^f^ HepG2 (30.7 μM)^f^ HT-29 (<10 μM)^f^ PC-3 (57.2 μM)^f^	48 h	Bai et al. (2012)
	Antitumor	Alismoxide	HeLa, Vero, U937	HeLa (>100 μg/mL)^f^ Vero (>100 μg/mL)^f^ U937 (>100 ± 0.04 μg/mL)^f^	72 h	Ellithey et al. (2013)
	Antitumor	10-O-methyl alismoxide	HeLa, Vero, U937	HeLa (38 ± 0.7 μg/mL)^f^ Vero (49.8 ± 0.5 μg/mL)^f^ U937 (50 ± 0.23 μg/mL)^f^	72 h	Ellithey et al. (2013)
	Antitumor	Alismol	HeLa, Vero, U937	HeLa (30 ± 17.2 μg/mL)^f^ Vero (49 μg/mL)^f^ U937 (N/T)^f^	72 h	Ellithey et al. (2013)

The routes of administration labeled on the upper right. (a. Intragastric administration; b. Cell fluid supernatant; c. Clinical trials; d. Intraperitoneal injection; e. Concentration of medium; f. IC_50_).

#### 4.4.1 Diuretic and inhibition of renal stone formation

*Rhizoma alismatis* is an important diuretic. Animal experiments showed that the diuretic effect of 80% ethanol extract of *R. alismatis* was stronger than that of water extract (Wang et al., 2008). Water, n-butanol, ethyl acetate and petroleum ether were used to extract *R. alismatis* directly. The results showed that the ethyl acetate extract of *R. alismatis* had the best diuretic effect (Huang et al., 2016). The results of HPLC analysis showed that the content of triterpenoids in the 80% ethanolic extract (Wang et al., 2008) and ethyl acetate extract (Huang et al., 2016) of *R. alismatis* were high, suggesting its diuretic effect may be related to the terpenoids. Zhang X. et al. (2017) extracted *R. alismatis* with 80% ethanol, conducted concentration under reduced pressure, successively adsorbed with AB-8 macroporous resin and polyamide resin, eluted with gradient ethanol, concentrated, and freeze-dried to obtain the total triterpene extract (TTE) of *R. alismatis*. Then they determined the content of triterpenes in TTE by high-performance liquid chromatography-quadrupole time-of-flight mass spectrometry (HPLC-Q-TOF-MS) and found that alisol A, alisol A 24-acetate, alisol B, alisol B 23-acetate, and alisol C 23-acetate were the main triterpenes in TTE, accounting for 72.2% (722.38 mg/g) of the total triterpene metabolites. And the results of diuretic experiment showed that TTE increased the urine volume of rats by 15.4%, 69.23% and 48.08% at the doses of 5, 20 and 40 mg/kg, respectively. Further study on the diuretic effect of different combinations of five triterpenoids in TTE found that when the ratio of alisol B 23-acetate: alisol B: alisol A 24-acetate: alisol A: alisol C 23-acetate was 7.2 : 0.6: 2.8 : 3.0: 6.4, the diuretic effect was the best (Zhang et al., 2017). Therefore, it can be ascertained that triterpenoids are the key functional substances of *R. alismatis* diuretic effect. In addition, studies have shown that the diuretic mechanism of *R. alismatis* is related to the reabsorption of kidney. The diuretic effect of *R. alismatis* can significantly increase the concentration of Na^+^, K^+^ and Cl^−^ in urine, and reduce the level of aquaporin-2 (AQP-2) mRNA in rat renal medullary cells and HK-2 cells (Li R. et al., 2020). As a response protein of antidiuretic hormone and a key protein of urine reabsorption, the reduction of AQP-2 explains the reason of *R. alismatis* diuretic (Kharin and Klussmann, 2023).

However, further studies have found that diuretic effect has duality (Chen et al., 2014; Feng et al., 2014). Feng et al. (2014) extracted *R. alismatis* with 95% ethanol, and obtained petroleum ether, ethyl acetate and n-butanol extracts by systematic solvent extraction after decompression and concentration. The diuretic experiment in rats showed that 100 and 400 mg/kg doses of ethyl acetate extract and 12.5, 25 and 50 mg/kg doses of n-butanol extract could significantly increase urine volume and urinary electrolyte excretion in rats, but 800 mg/kg dose of ethyl acetate fraction and 75 mg/kg and 100 mg/kg doses of n-butanol fraction significantly reduced. In the subsequent experiments, the 95% ethanol extract of *R. alismatis* was subjected to diuretic experiments. It was found that the urine volume of rats was increased at the doses of 2.5, 5 and 10 mg/kg, but decreased at the doses of 20, 40 and 80 mg/kg, while the water extract of *R. alismatis* was not significantly different. At present, it is believed that the dual diuretic effect of *R. alismatis* may be related to the sodium chloride cotransporter in the distal renal tubule. Lin Ruichao believes that the ethanol extract of *R. alismatis* may inhibit the activity of sodium-chloride cotransporter in the distal renal tubule at the doses of 2.5, 5 and 10 mg/kg, and increase the transport of Na^+^ to the distal segment of the distal renal tubule and the upper collecting duct, and the increase of Na^+^ concentration leads to the increase of K^+^ loss. Stimulation of the aldosterone-sensitive sodium pump increases Na^+^ reabsorption to exchange K^+^ and H^+^ for urine excretion. At the doses of 20, 40 and 80 mg/kg, the inhibitory effect of the ethanol extract of *R. alismatis* on the sodium-chloride cotransporter in the distal renal tubules would fail due to the huge permeability difference, and the urine would be reduced due to the passive absorption of water and the effect of antidiuretic hormones. This view does not explain the different effects of different concentrations of *R. alismatis* ethanol extract on sodium-chloride cotransporters in distal renal tubules. The specific mechanism of action is not clear and needs further study. It is worth noting that the diuretic dose of 95% ethanol extract prepared by Lin Rui chao et al. was significantly lower than that of the enriched n-butanol fraction (which is rich in diuretic active triterpenes). Except for the batch differences of medicinal materials and the sensitivity of experimental animals, the diuretic effect of 95% ethanol extract was significantly better than that of four different extraction fractions (Water, n-butanol, ethyl acetate and petroleum ether extract), suggesting that there may be a synergistic interaction between diuretic and non-diuretic components in *R. alismatis* extract to enhance diuretic effect. However, this very aspect remains unexplored in current research.

Because of its excellent diuretic effect, *R. alismatis* is often used for the treatment of kidney stones. CaOx stones are the most common in kidney stones, which mainly exist in the form of CaOx monohydrate (COM) and CaOx dihydrate (COD). Compared with COD crystals, COM crystals show stronger cell affinity and significant cytotoxicity. Therefore, Wang et al. (2024) established a cell injury model by stimulating HK-2 cells with 100 nM calcium oxalate monohydrate, and found that *R. alismatis* polysaccharide could significantly improve the survival rate of HK-2 cells by reducing the levels of inflammatory factors such as NLRP3, TNF-α, IL-6 and NO, inhibiting apoptosis, and reducing the expression of adhesion molecule CD44 on the surface of HK-2 cells. Cheng and Ouyang (2023) believed that the carboxymethyl derivative of *R. alismatis* polysaccharide could alleviate the damage of HK-2 cells and reduce the formation and damage of kidney stones by affecting the crystal structure of calcium oxalate. In addition, some studies have pointed out that there is a certain correlation between obesity and kidney stones (Kim et al., 2022), and the water and dampness effects of *R. alismatis* may also alleviate the occurrence of kidney stones by regulating lipids. It can be seen that there may be a complex mechanism affecting the metabolism of water, sugar and lipid in the whole body to treat kidney stones.

#### 4.4.2 Anti-inflammatory and antioxidant effects

Although *R. alismatis* has not been used as an anti-inflammatory drug, recent studies have found that it shows good anti-inflammatory effects in different disease models. The 80% ethanol extract of *Alisma orientalis* can alleviate LPS-induced acute lung injury by inhibiting the expression of inflammatory genes such as NF-κB, COX-2, IL-1β and iNOS (Han et al., 2013). Monomer components within *R. alismatis*, such as Alisol A (Wang et al., 2020), plantain A (Huang et al., 2017), Alismol (Liu, 2012), alisol A 24-acetate, alisol B 23-acetate (Wang et al., 2019), alisols A monoacetate (Matsuda et al., 1999), alisols B (Matsuda et al., 1999), alisols B monoacetate (Matsuda et al., 1999), alisols E (Matsuda et al., 1999), Alisol F, alisols G (Matsuda et al., 1999), alisols K-23-acetate (Matsuda et al., 1999), alisols N-23-acetate (Matsuda et al., 1999), alisols 11-deoxyalisol B (Matsuda et al., 1999) and 25-anhydro-Ali-F (Ma et al., 2016), all of which have been documented to have potent anti-inflammatory activity.

In addition to anti-inflammatory, *R. alismatis* extract also showed good antioxidant activity. The 80% ethanol extract of *R. alismatis* can alleviate LPS-induced acute lung injury by enhancing the activity of Nrf2 and promoting the expression of its downstream regulatory genes NQO-1, HO-1 and GCLC (Han et al., 2013). The water extract of *R. alismatis* can significantly reduce the levels of reactive oxygen species (ROS) and active aldehydes in HepG2 cells induced by palmitic acid, and alleviate steatosis and cell damage in HepG2 cells (Han et al., 2012). Syringaresinol, a phenylpropanoid compound in *R. alismatis*, showed good antioxidant properties in DPPH scavenging assay (Zhao et al., 2017). Based on the aforementioned findings, the anti-inflammatory and antioxidant activities of individual active components have been primarily demonstrated in in vitro cell-based assays, whereas the corresponding efficacy *in vivo* has been observed mainly with *R. alismatis* extracts and their formulated preparations. This suggests that synergistic interactions among the active components of *R. alismatis* may contribute to its anti-inflammatory and antioxidant effects *in vivo*.

#### 4.4.3 Blood-lipid regulation

Clinical experiments have found that *R. alismatis* can significantly reduce blood lipids and have been verified in several animal models of hyperlipidemia. Zexie Decoction, as a classical prescription, is widely used in clinical treatment of hyperlipidemia. Cell and mouse models have confirmed that Zexie Decoction can significantly improve symptoms such as lipid accumulation and insulin resistance by inhibiting the activity of SREBPs and the expression of its target gene FKBP38 (Xie et al., 2022). In the hyperlipidemia model, CYP450 enzyme subtype enzyme activity decreased, which will affect the liver’s ability to metabolize therapeutic drugs. Ju et al. (2023) conducted a study on the disformulation of Zexie Decoction (water extract) and compared the effects of Zexie Decoction group, single medicine Zexie group and Baizhu group on CYP450 enzyme activity in hyperlipidemia mice. It was found that Zexie Decoction can restore the expression of CYP3A4 and enhance its activity, in which Zexie played a leading role in Zexie Decoction.

A large number of studies have shown that *R. alismatis* has good lipid-lowering activity when use alone. The 95% ethanol extract of *R. alismatis* can effectively reduce the levels of serum total cholesterol (TC), triglyceride (TG), low density lipoprotein cholesterol (LDL-C) and high-density lipoprotein cholesterol (HDL-C) in hyperlipidemia rats. Nineteen biomarkers were obtained by urinary metabolomics, among which ascorbalamic acid, indolelactic acid, 1-hydroxypyrene, 3-methyluridine and 4-heptanone. 4-aminohippuric acid, hypoxanthine, creatinine and indole-3-carboxylic acid were positively correlated with hyperlipidemia. Whereas metabolites such as xanthosine, urocanic acid, propionylcarnitine, xanthine, cytidine, N-acetylneuraminic acid, phenylacetylglycine, methylhippuric acid, D-arginine and hippuric acid were negatively correlated with it. After taking *R. alismatis*, these markers tended to normal levels and alleviated the dysfunction caused by changes in metabolites (Miao et al., 2016). Hongliang Jiang (Yan et al., 2022) et al. combined metabolomics, network pharmacology, and lipidomics to identify 18 potential active compounds and 83 potential therapeutic targets in the plasma of hyperlipidemic mice based on metabolomics. They focused on the PPAR signaling pathway and validated by qPCR that ALB, TNF, IL1B, MMP9, PPARA, and PPARG may be the 6 upstream key targets regulated by *R. alismatis*. Kang-Beom Kwon et al. (Park et al., 2014) found that 70% ethanol extract of *R. alismatis* could inhibit the differentiation of OP9 adipocytes in bone marrow stromal cells by reducing the expression of C/EBP β in mice. Haoxin Wu et al. (Liu et al., 2023) found that the area of plaques, blood HDL-C and TG, and serum CHO and LDL-C levels were improved in APOE−/− hyperlipidemic mouse models after taking *R. alismatis* extract. Combining network pharmacology analysis and validation, it was suggested that *R. alismatis* may improve its atherosclerotic progression by inhibiting the phosphorylation of PI3K/AKT and the expression of SREBP-1.

The lipid-regulating effects of *R. alismatis* decoctions and single extracts have closed related with its unique triterpenoids. Such as alisol G, alisol A 23-acetate, 16-oxo-11-anhydroalisol A, alisol B, alisol B 23-acetate, and alismanol J may improve HepG2 cell uptake of LDL by regulating PCSK9 (Zhang et al., 2022). Acyl coenzyme a-cholesterol acyltransferase (ACAT), one of the lipid-regulating targets of *R. alismatis*, plays an important role in the balance of cholesterol metabolism in cells and organisms. Its activity is influenced by the ratio of alisol A, alisol B, and 23-acetyl alisol C, with the n-terminal lipid-regulating activity of ACAT being stronger than the transmembrane domain when the ratio is 3:1:1 (Xu et al., 2020). In summary, the lipid-lowering effect of *R. alismatis* is related to triterpenoids, which may affect the key indicators of lipid metabolism diseases such as TC, TG, LDL-C and HDL-C by affecting the intake of low-density lipoprotein in hepatocytes and inhibiting the differentiation and autophagy of adipocytes.

#### 4.4.4 Regulating blood glucose

In TCM, *Rhizoma alismatis* is frequently utilized as one of the prescriptions for the management of diabetes. Studies have manifested that *R. alismatis* can effectively reduce blood glucose levels both *in vivo* and *in vitro* (Lau et al., 2008; Yang et al., 2004). Polysaccharide is considered to be one of the effective metabolites of hypoglycemic. For example, the T2DM model rats established by high-fat diet combined with intraperitoneal injection of streptozotocin (30 mg/kg) were fed with *R. alismatis* polysaccharide for 6 weeks, the levels of SOD and GSH-Px in liver tissue of rats were decreased, and insulin resistance and lipid metabolism were improved (Zhang et al., 2018). Studies have also shown that *R. alismatis* polysaccharide may improve the symptoms of diabetic rats such as dry and yellow hair, significant weight loss and renal injury by regulating PPAR-γ/LXR-α/ABCG1 signaling pathway (Dong et al., 2023). Besides polysaccharide, the ethanol extract of *R. alismatis* can slow down the release of glucose by inhibiting the activity of α-glucosidase and increase the glucose uptake of 3T3-L1 adipocytes to inhibit adipogenesis (Li and Qu, 2012). *In vivo* and *in vitro* experiments confirmed that alisol A (Lin et al., 2023) and epoxy alisolene (Zhang et al., 2019) could affect cell glucose absorption capacity and reduce fasting blood glucose in type 2 diabetic mice, respectively. The development of its hypoglycemic effect requires further validation of its efficacy and safety in humans, as well as clarification of the specific mechanisms of action of its various active constituents. Concurrently, attention must be paid to the rational use of *R. alismatis*, including investigations into its potential for concomitant use with existing medications. Careful monitoring is essential to ensure medication safety.

#### 4.4.5 Hepatoprotective effect

The hepatoprotective effect of *R. alismatis* is primarily manifested in the alleviation of drug-induced liver injury and the treatment of fatty liver, hepatitis, and liver fibrosis (Choi et al., 2019). For drug-induced liver injury, Zexie prescription, extract and monomer components have a certain protective effect. Zexie Shugan Decoction can reverse thioacetamide (TAA)-induced liver injury and fibrosis by inhibiting α-SMA protein, reducing collagen area and fibrosis score (Sun et al., 2020). The methanol extract of *R. alismatis* was continuously fed to rats injected with bromobenzene for 1 week, which increased the content of epoxide hydrolase and glutathione s-transferase, decreased the content of aminopyridine n-demethylase and aniline hydroxylase, and alleviated the liver injury caused by bromobenzene (Hur et al., 2007). In addition, the methanol extract of *R. alismatis* inhibited tunicamycin-induced triglyceride accumulation by inhibiting endoplasmic reticulum stress (Jang et al., 2015), alleviating its lipid toxicity. Alisol B 23-acetate can reduce the intake of ANIT and increase the excretion of bile acid by down-regulating NTCP protein and up-regulating efflux transporters (Bsep, Mrp2 and Mdr2). Alisol B 23-acetate can also reduce bile acid synthesis by inhibiting CYP7A1 and CYP8B1, and increase Bal and Baat expression by up-regulating Sult2a1 gene to increase bile acid metabolism. From the perspectives of enhancing the ability of liver to metabolize drugs and maintaining the bile acid homeostasis of the liver, the liver injury of ANIT-mediated cholestasis hepatotoxicity mice was effectively alleviated (Meng et al., 2015). Alisol F (20 mg/kg/d, i. p.) can effectively reduce the levels of AST, ALT and inflammatory factors induced by LPS/d-gal by affecting the MAPKs/NF-kB/STAT3 pathway, and improve liver pathological damage (Bi et al., 2017).

At the same time, *R. alismatis* is considered to be an effective candidate drug for NAFLD (Choi et al., 2019). Zexie Decoction is commonly used in clinical practice. Comparing the differentially expressed genes, differentially expressed lipid molecules and differentially expressed intestinal microflora between the Zexie Decoction group and the control group (Zhang et al., 2022), it is speculated that Zexie Decoction may play a therapeutic role by regulating fatty acid synthesis, correcting lipid metabolism disorders and reducing inflammatory response. Sirt1 and AMPK are the key to Zexie Decoction promoting fatty acid oxidation (FAO) (Cao et al., 2022). Biao et al. (2022) found that Danshen Zexie Decoction could inhibit ROS/NLRP3/IL-1β signaling pathway and improve many liver function indexes by activating Nrf2 in NAFLD rat model fed with high fat diet. Continuous feeding of obese mice induced by high-fat diet at doses of 100, 300 mg/kg/d can effectively inhibit liver ER stress and hepatic steatosis. In addition to the decoction, *R. alismatis* extraction can also inhibit the steatosis caused by oleic acid, palmitic acid (Jeong et al., 2016), and high-fat diet (Jang et al., 2015); reduces oxidative stress and inflammation levels in the liver (Hong et al., 2006).

#### 4.4.6 Anti-tumor effect

A considerable number of studies have demonstrated that extracts and monomer metabolites of *R. alismatis* significantly inhibit the proliferation of various tumor cells, including breast cancer (Lou et al., 2019), colorectal cancer (Xu et al., 2015), liver cancer (Li L. et al., 2020), lung cancer (Liu et al., 2019) and ovarian cancer (Zhang et al., 2016). Wu et al. (2023) proposed that *R. alismatis* is a potential low-toxicity anticancer agent. Its active ingredient, alisol (a tetracyclic triterpenoid alcohol), can be metabolized into various derivatives and is suggested to inhibit cancer cell proliferation and migration by modulating key signaling pathways such as mTOR, Bax/Bcl-2, CHOP, caspase, NF-κB, and IRE1. However, the clinical application of *R. alismatis* as an anti-tumor drug remains limited, as the existing evidence primarily derives from *in vitro* studies (Table 1; Figure 7). Therefore, it is essential to further validate its anti-tumor efficacy and elucidate the underlying mechanisms, particularly through *in vivo* experiments, to account for critical factors like bioavailability and metabolic processing.

#### 4.4.7 Alignment and discrepancies: modern pharmacology and traditional medicine

The investigation into *R. alismatis* presents a compelling narrative of convergence and expansion between its millennia-old applications in TCM and contemporary pharmacological inquiry. At its core, a remarkable consistency exists, where modern science provides a mechanistic foundation for ancient wisdom. TCM attributes to *R. alismatis* the properties of diuresis promotion, dampness elimination, turbidity resolution. Modern pharmacology robustly validates this. However, this consistency is elegantly complemented by a critical divergence and deepening of understanding. The discovery of its effects beyond the traditional scope, such as significant anti-inflammatory, anti-diabetic nephropathy, and potential anti-cancer properties, exemplifies this divergence. These are not contradictions but rather expansions, revealing new therapeutic dimensions unexplored by classical texts. However, the tonifying and nourishing functions and skin-protective effects recorded in ancient texts have not yet been confirmed by modern pharmacology. Are they undiscovered secrets or previously misunderstood concepts? This undoubtedly warrants in-depth investigation.

According to the holistic framework of TCM, *R. alismatis* possesses sweet and cold properties. Upon ingestion, its therapeutic actions are directed toward, or it “guides” its effects to, the Kidney and Bladder meridians. Coincidentally, toxicology has found that long-term excessive use of *R. alismatis* can cause kidney damage (see Section 4.5 for details). Thus, the dialogue between tradition and modernity regarding *R. alismatis* is not one of conflict but of synergistic validation and discovery. Modern pharmacology confirms the profound intuition of traditional practice by elucidating the scientific “how,” while simultaneously transcending it by uncovering novel biological activities, thereby enriching the potential applications of this ancient remedy in modern evidence-based therapy.

### 4.5 The toxicity of *Rhizoma alismatis*

**FIGURE 8:**
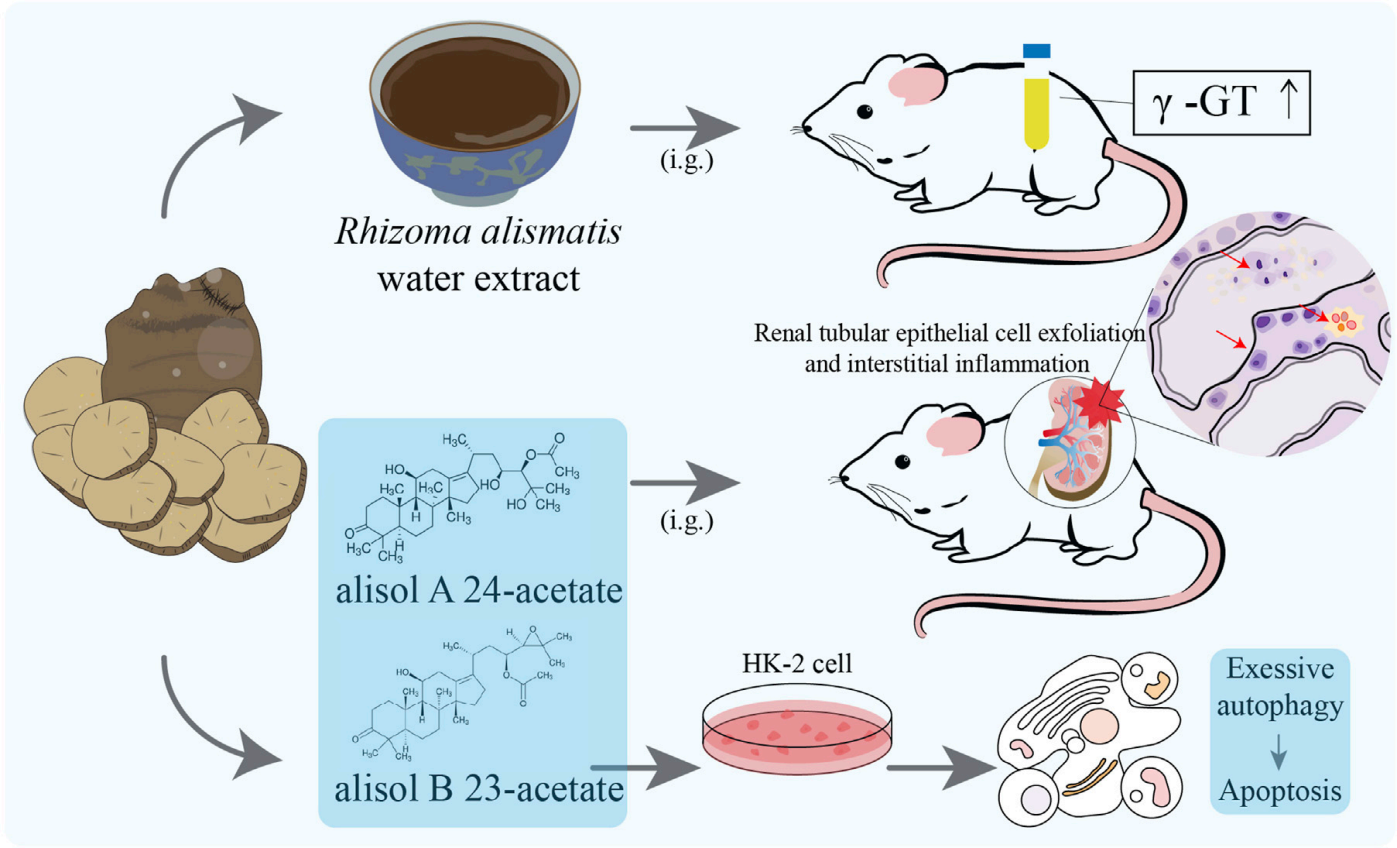
Toxicity study of *Rhizoma alismatis*. Urinary γ-glutamyltransferase (γ-GT) activity was significantly increased in 60 days after continuous administration of the normal dose of 20 and 40 times of *Rhizoma alismatis* water extract; Inflammation of the renal interstitium, exfoliation of renal tubular epithelial cells and morphological changes were observed in Alisol B 23-acetate (0.4 g/kg/day) and Alisol A 24-acetate (0.5 g/kg/day) group rats (6 months) and marked with red arrows; After Alisol B 23-acetate (15 μM) and alisol A 24-acetate (6 μM) were added to the culture medium, HK-2 cells showed significant apoptosis and autophagy.

The toxicity of *R. alismatis* is controversial in clinical application and basic research, which also affects people’s understanding of its efficacy. Some ancient TCM books stated that *R. alismatis* is cold, sweet and non-toxic. For instance, “Shennong’s Herbal Classic” described that long-term use of *R. alismatis* can enhance the acuity of ears and eyes, suppress hunger, prolong lifespan, lighten the body and enhance complexion. “Leigong processing medicinal solution” recorded that *R. alismatis* is sweet and salty, cold and non-toxic. “Bencao Jingjie” also held the opinion that the taste of *R. alismatis* is sweet and non-toxic. On the contrary, there are also records suggesting that *R. alismatis* has a certain degree of toxicity. The toxicity of the whole plant of *R. alismatis* is mentioned in “The main toxic plants in the south and their treatment of poisoning”, with the underground root being more toxic. The symptoms of poisoning include itching, redness, blistering on the skin, abdominal pain, diarrhea and other gastrointestinal symptoms after consumption (Cash crop team of Guangdong Agriculture, 1970). In the “commonly used traditional Chinese medicine and adverse reactions” record: “A large dose or long-term application of *R. alismatis* can lead to water electrolyte imbalance and hematuria, and even acidosis, and can cause nausea, vomiting, abdominal pain and liver function impairment”, clearly proposing its liver and kidney toxicity (Chen and Zhou, 1998). The 2020 edition of the “Pharmacopoeia of the People’s Republic of China” divides toxic Chinese medicines into three categories: 1. high toxicity, 2. toxic, 3. small toxicity; however, *R. alismatis* is not included. Currently, there is a big controversy regarding whether *R. alismatis* is toxic. According to the existing experimental evidence, the toxicity of *R. alismatis* is conditional and acceptable. The following will discuss the research reports on the toxicity of botanical drug extracts and monomer metabolites of *R. alismatis* (Figure 8).

#### 4.5.1 Toxicity study of *Rhizoma alismatis* extracts

The metabolites of Chinese herbal medicine are complex and diverse, and the effective metabolites of Chinese herbal medicine are often extracted and concentrated by water and alcohol extraction. Here, the toxicity will be discussed based on the water extract and alcohol extract (Table 2).

**TABLE 2:** Summary of toxicity of *Rhizoma alismatis* compound and water/alcohol extracts.

No.	Prescription/Extraction solvent	Extracting solution	Dosage	Duration	Experimental subject	Results	Mechanism or method	Refs
1	*Rhizoma alismatis* 80% ethanol extract	Ten times the volume of 80% ethanol was boiled, filtered, repeated once, concentrated and combined	0.18, 18, 36 g/kg/d (The dosage of Rhizoma alismati in low dose group was equivalent to that in Liuwei Dihuang pill, and the dosage of *Rhizoma alismati* in middle and high dose groups was 20 and 40 times the normal dosage)	6 months	Female SD rats	Low dose group (0.18 g/kg/d): no obvious nephrotoxicity Middle and high dose groups: interstitial inflammatory cell infiltration, edema and morphological changes of renal tubular epithelial cells	LCN2/Kim-1	Wang et al. (2016)
2	*Rhizoma alismatis* water extract	add 4–6 times water, soak, decoction, heat, concentrate and filter	20, 50 g/kg/d	8 weeks	Male Sprague-Dawley rats (partial nephrectomy)	Low-dose group of nephrectomy group: shaggy, reduced activity, diet and body weight loss High-dose group of nephrectomy group: shaggy, reduced activity, diet and body weight loss after 1 week Low and high-dose group all showed inflammatory cell infiltration and tubular damage in the remnant kidney interstitium	Urine routine, serum creatinine and renal tissue pathological examination were performed	Zhu et al. (2007)
3	*Rhizoma alismatis* water extract	Add 10 times of water to soak, decoction, filter and repeat with 5 times of water, combine the filtrate and concentrate	8.3, 16.7, 33.3 g/kg/d	60 days	Male SD rats	There was no significant effect on the normal growth and development of rats and the absorption and metabolism of food		Duan et al. (2004)
4	Shenze Shugan capsule (*Rheum palmatum* L. (Dahuang), *Prunella vulgaris* L. (Juemingzi) and *Rhizoma alismatis* (Zexie))	Provided by Jilin Aodong Pharmaceutical Group Yanji Company	Shenze Shugan capsule 27.68 g/kg/d (The dose is equivalent to 60 times the human clinical dose in the whole formula)	8 weeks	Male SD rats	There was no significant difference in body weight Serum creatinine and urea nitrogen were in the normal range, no significant changes were observed in the alismatid and mixed groups Dahuang group and Juemingzi group showed renal tubular degeneration and expansion		Huang et al. (2024)
5	Shenze Shugan capsule (Same as above)	alisol B 23-monoacetate alisol A		24 h	HK-2 cell	IC_50_ (alisol B 23-monoacetate 32.3 μM, alisol A 46.8 μM)	Bcl-2/Bax pathway of apoptosis	Huang et al. (2024)
6	Zexie decoction additives (*Rhizoma alismatis* (Zexie), *Atractylodes macrocephala* Koidz. (Baizhu), *Lycopus lucidus* var. *hirtus* Regel (Zelan), *Acorus tatarinowii* Schott (Shichangpu)) = 7:3:5:5)	Add 8 times of water to soak, decoction, filter and repeat with 6 times of water, combine the filtrate and concentrate	15, 30, 60 g/kg/d (The dose was equivalent to 14,28, and 55 times the clinical dose in humans)	90 days	Male Wistar rats	There were no significant changes in hematology, serum biochemical indexes and organ coefficients, and most organ tissues (liver, kidney, ovary and testis) were normal. In the 60 g/kg/d group, renal tubular epithelial cells were edema, necrosis and exfoliation in 2 cases, and hepatic congestion was observed in 1 case		Chen et al. (2015)

The water extract of *Rhizoma alismatis* contains more polysaccharides, while its alcohol extract is mainly composed of nitrogen-containing compounds, phenylpropanamide compounds, and terpenoids. The difference in material composition often affects its efficacy and toxicity. Wang et al. (2016) boiled *R. alismatis* with 10 times the volume of 80% ethanol, filtered, and concentrated to obtain the ethanol extract of *R. alismatis*, and carried out a long-term nephrotoxicity experiment on the ethanol extract of *R. alismatis*. After 6 months of continuous administration, renal interstitial inflammatory cell infiltration and renal tubular epithelial cell edema were found in rats in the middle and high dose groups (20 and 40 times of the equivalent dose of *R. alismatis* in Liuwei Dihuang Pills). However, the low dose group (the equivalent dose of *R. alismatis* in Liuwei Dihuang Pills) did not show significant nephrotoxicity.

Although the water extract of *R. alismatis* has weaker diuretic and relieving drug-induced liver injury than the alcohol extract (Feng et al., 2014; Jang et al., 2021), its water extract may also be toxic. Zhu et al. (2007) fed normal rats and right nephrectomy rats with 20 and 50 g/kg water extract of *R. alismatis* for 8 weeks respectively. It was found that the right nephrectomy rats fed with water extract of *R. alismatis* showed adverse reactions such as loose hair, reduced activity, reduced diet and weight loss. The renal sections showed renal interstitial inflammatory cell infiltration and tubular damage.

Duan et al. (2004) studied its subchronic toxicity (60 days) on normal rats with 50, 100 and 200 times of safe dosage of the water extract of *R. alismatis*. The blood urea nitrogen (BUN) in the high dose group showed an increasing trend, but did not exceed the normal range; the activity of urinary γ-glutamyltransferase (γ-GT) in the middle and high dose groups was significantly higher than control group. However, histopathological examination showed that there was no obvious pathological damage in the liver, kidney, spleen and testis of the rats in each experimental group. Based on these studies of *R. alismatis* extract, the metabolism of *R. alismatis* requires the participation of the kidney, and its toxicity depends more on the function of the kidney. Therefore, *R. alismatis* needs special attention for patients with renal dysfunction or injury, and cannot be taken for a long time and overdose.

#### 4.5.2 Toxicity study of monomer compounds of *Rhizoma alismatis*

In this section, the literature reports of effective monomers of *Rhizoma alismatis* were collected, and the literature was sorted out according to monomer composition, dose, model, toxicity results, mechanism of action, and monomer category (Table 3). The results showed that the toxicity study of the main active ingredient terpenoids in *R. alismatis* mainly stayed in cell experiments, while animal experiments and human experiments were less reported. In addition, *R. alismatis* contains the same metabolites of other plants, such as nicotinamide, indazole, β-sitosterol and so on. These compounds have been proved to have potential toxicity and adverse reactions (Chong et al., 2002; Matsumura et al., 2013), but the content of *R. alismatis* in normal use is much lower than its adverse reaction dose. Subsequently, some in-depth toxicity studies on the terpenoids of *R. alismatis* are needed.

**TABLE 3:** Summary of toxicity of monomer compound of *Rhizoma alismatis* chemical constituents.

No.	Monomer compound	Dosage	Duration	Cell/animal	Results	Mechanism or method	Category	Refs
1	Alisol A 24-acetate	768, 384, 192, 96, 48, 24, 12, 6, 3 μM	24 h	Human renal proximal tubular (HK-2) cells	Apoptosis Damage marker molecules Kim-1、Clusterin and TFF-3↑	Apoptosis↑ Bcl-2↓, Bcl-xl↓	a	Wang et al. (2017)
2	Alisol A 24-Acetate	0.5 g/kg/day	6 months	SD rats	Interstitial inflammation, renal tubular epithelial cell exfoliation and morphological changes	Inhibition of PI3K/Akt/mTOR signaling pathway	a	Wang et al. (2017)
3	Alisol B 23-acetate	15 μM	24 h	Human renal proximal tubular (HK-2) cells	Apoptosis	MTT	a	Wang et al. (2017)
4	Alisol B 23-acetate	0.4 g/kg/day	6 months	SD rats	Interstitial inflammation, renal tubular epithelial cell exfoliation and morphological changes	Inhibition of PI3K/Akt/mTOR signaling pathway	a	Wang et al. (2017)
5	Alismoxide	5 uM	6 days	Skeletal muscle cell	Increased cell proliferation, form myotubes and multinuclear fused myotubes	CCK8	b	Hien et al. (2023)
6	Nicotinamide	0–300 mM	24 h	Microvascular endothelial cell	Endothelial cell toxicity within 25–300 mM but no significantly different in 5–15 mM		c	Chong et al. (2002)
7	Indazole	50 mg/kg		Male SPF ddY mice	had no effect on spontaneous activity but induced hypothermia The median toxic dose (TD 50) of indazole was 52.3 mg/kg by the minimal motor impairment test		c	Matsumura et al. (2013)
8	Syringaresinol	0–100 μM Syr	24 h	HepG2 and HT29 cells	No cytotoxic effects	resazurin reduction and comet assays	d	Kirsch et al. (2020)
9	Pinoresinol	100 μM	24 h	H4IIE cells	Negative effect to cell viability, leading to approximately 25% cell death		d	Steffan et al. (2005)
10	Umbelliferone	50, 100 and 200 mg/kg		Swiss male mice	signs of toxicity after acute administration of the compound were not observed		d	Cruz et al. (2020)
11	β-Sitosterol	250, 500, 1000 μg/kg	60 days	Albino rats	No clear evidence of any gross or microscopic lesions either in the liver or kidney		e	Malini and Vanithakumari (1990)
12	Ferulic acid	5, 10 mg/kg/d	28 days	Wistar rats	No nephrotoxicity or hepatotoxicity		f	Slighoua et al. (2023)
13	Succinic acid	5, 10, 25, 50 and 100 μM		MRC-5, CAKI-2 and ACHN	Cell viability↓in cancer cells	WST-1	f	Kasarci et al. (2021)

Triterpenoids were labeled as a, sesquiterpenoids as b, nitrogenous compounds as c, phenylpropanoids as d, steroidal compounds as e, and phenolic acids as f.

##### 4.5.2.1 Toxicity of terpenoids

The terpenoids in *R. alismatis* include triterpenoids, sesquiterpenes and diterpenes, among which the triterpenoids (133) in *R. alismatis* have been studied most deeply. Triterpenoids’ chemical skeletons are prototerpane-type tetracyclic triterpenes represented by alisol A-X and its derivatives and alismanol A-Q, which are also characteristic metabolites of *R. alismatis*. Alisol A 24-acetate, alisol B 23-acetate, Alisol A and Alisol B are the main chemical components in the water extract of *R. alismatis* (Luo et al., 2021; Wang et al., 2017).

Alisol B 23-acetate and alisol A 24-acetate were continuously fed to rats at 0.4 g/kg/day and 0.5 g/kg/day for 6 months, respectively. Inflammation of renal interstitium, exfoliation of renal tubular epithelial cells and morphological changes were observed in two group rats, suggesting that these two compounds have potential renal toxicity and could potentially be the primary cause of toxicity elicited by *R. alismatis*. *In vitro* experiments demonstrated that alisol B 23-acetate and alisol A 24-acetate (treated with 6 μM and 15 μM, respectively) could significantly induce HK-2 cell apoptosis and autophagy by down-regulating the levels of Bcl-2 and Bcl-xl and inhibiting the PI3K/AKT/mTOR pathway. Simultaneously, the expression of Kim-1, Clusterin, and TFF-3, which are renal drug toxicity biomarkers, was enhanced. Evidently, the compounds alisol B 23-acetate and alisol A 24-acetate were verified as nephrotoxic compounds. Additionally, research indicated that the inhibition of autophagy could effectively reverse the apoptosis induced by alisol B 23-acetate and alisol A 24-acetate, suggesting that the nephrotoxicity of alisol B 23-acetate and alisol A 24-acetate was associated with their excessive induction of autophagy through inhibiting PI3K/Akt/mTOR signaling pathway (Table 3).

Although compounds, alisol B 23-acetate and alisol A 24-acetate showed good growth inhibitory effects on cancer cells such as H460, MCF-7, PC-3 and HepG2 (Table 2), their IC_50_ values were all greater than 17.82 μM. Obviously, renal epithelial cell HK-2 is more sensitive to it, that is, it may have caused adverse reactions of renal injury while exerting anti-cancer effects. Therefore, the potential anti-cancer activity of these compounds remains to be discussed. Other triterpenoids (Table 3, column a) have been reported in addition to the toxicity of tumor cells and macrophages (Table 3), there are almost no reports on toxicity and adverse reactions such as liver and kidney.

At present, 58 sesquiterpenes have been isolated from *R. alismatis*. According to their structures, they can be divided into guaiacol type, apostichopus type, germacane type, xanthene type and phyllane type (Mengxiang et al., 2023). The guaiacol type represented by alismoxide orientalol A ∼ G and its derivatives is the main type. At present, the research on its toxicity mainly stays in cell experiments (Table 3). Four diterpenes were isolated from *R. alismatis*, including oriediterpenol, oriediterpenol, oriediterpenoside, 12-deoxyphorbol-13α-pentadecanoate. At present, there is no report on the above diterpene monomer components.

##### 4.5.2.2 The toxicity of other metabolites

Other metabolites of *R. alismatis* include nitrogen-containing compounds, phenylpropanoids, steroids, flavonoids, phenolic acids, and aliphatic hydrocarbons and derivatives. The toxicity studies of some compounds are listed in Table 3 (category: c-f).

Nicotinamide and indazole are nitrogen-containing compounds that have been studied for toxicity in *R. alismatis*. Nicotinamide is a form of vitamin B3, which has a dual role in cell growth. It enhances cell viability and replication at a dose close to 5 mM (Chong et al., 2002), and when the dose is higher than 20 mM, it can lead to apoptosis. The results of human intervention test showed that children continued to take 25–50 mg/kg per day for 5 years, and the elderly took 1.5 g NAM twice a day for 6 months without adverse reactions, indicating its safety. At present, the reported adverse reactions of nicotinamide are mainly headache, dizziness and vomiting caused by large-scale use on an empty stomach. Such adverse reactions can gradually recover after stopping taking (Hwang and Song, 2020), and the concentration contained in *R. alismatis* is far less than the concentration of side effects. Indazole has anti-inflammatory, antibacterial, anti-HIV, anti-arrhythmia, anti-fungal and anti-tumor effects. At present, there are about 40 kinds of therapeutic agents based on indazole for clinical application or clinical trials. At a dose of 50 mg/kg, there is no significant effect on mice, but it will lead to a decrease in body temperature in mice. The minimum motor dysfunction test shows that the median toxic dose of indazole is 52.3 mg/kg (Matsumura et al., 2013). Umbelliferone, a phenylpropanoid compound, has been shown to have anti-diabetes, anti-cancer, anti-infection, anti-rheumatoid arthritis, neuroprotection, and improvement of liver, kidney, and myocardial tissue damage. However, it has a dose-dependent cytotoxicity (Lin et al., 2023). At a concentration of 200 μg/mL, it inhibits the activity of human bone marrow stem cells. When the concentration exceeds 500 μM, it exhibits cytotoxic effects on human, rat, mouse, and rabbit hepatocytes; however, no obvious acute toxicity symptoms were observed in the acute toxicity test of umbelliferone in mice at 200 mg/kg (Cruz et al., 2020). The steroidal compound β-sitosterol from *R. alismatis* has been proven 30 years ago that 60 days of continuous administration at a dose of 1 mg/day does not cause liver and kidney toxicity, and has developed a variety of health products that are considered to be non-toxic compounds. Phenolic acids are a class of organic acids containing phenolic rings, which are present in a variety of plants and have anti-oxidation, anti-inflammatory, and hypotensive effects. Ferulic acid in *R. alismatis* was continuously fed at a dose of 5–10 mg/kg for 28 days without hepatorenal toxicity. No significant cytotoxicity was observed in MRC-5 cell lines treated with 25 μM and 50 μM *in vitro* (Kasarci et al., 2021). In addition to the mentioned metabolites, there are more than 100 kinds of non-terpenoids in *R. alismatis*, and its toxicity research has not been reported in the relevant literature.

### 4.6 Processing of *Rhizoma alismatis*

The processing methods of *R. alismatis* are various. Lei Gong’s theory of processing was first recorded in the Southern and Northern Dynasties: fine file, wine soaking for one night, wet out, dry out. In the Song Dynasty, the processing methods of wine soaking, stir-frying, steaming after wine soaking were added. To Ming Dynasty, the processing methods of water soaking, steaming, simmering and steaming after rice swill soaking appeared. In the Qing Dynasty, in addition to the main processing methods of the previous generation, the processing methods of salt water mixing, salt water stir-frying, wine stir-frying, wine mixing and baking were also put forward (Zhong et al., 2005).

**FIGURE 9:**
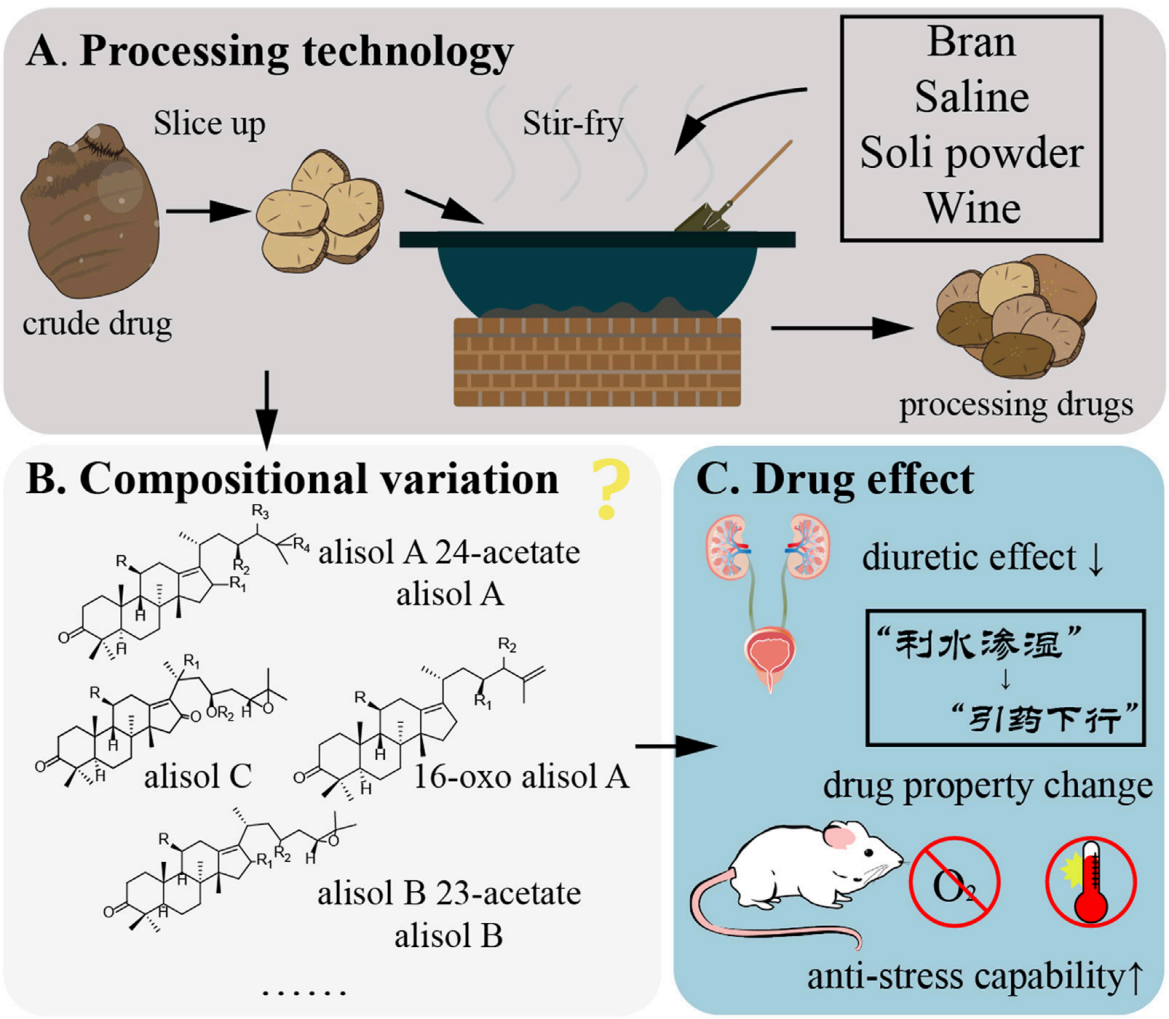
Processing methods of *Rhizoma alismatis*. **(A)** *Rhizoma alismatis* were sliced up and stir-fry with bran, saline, soli powder and wine. **(B)** *Rhizoma alismatis* compounds change after different processing methods **(C)**. Processing affect the *Rhizoma alismatis* functions including diuretic effect and anti-stress capability.

The processing of *Rhizoma alismatis* has been continuously developed and adjusted, and the following mainstream processing approaches have emerged, including stir-frying, salt stir-frying, bran stir-frying, and soil stir-frying. Currently, the most commonly used processing varieties are bran stir-frying and salt stir-frying (Figure 9A) (Wang, 1993). The processing method is as follows: bran stir-frying: take bran, sprinkle it into a hot pot, wait until smoke emerges, add *R. alismatis* slices (RA), stir-fry until they turn brown, remove it, sieve the bran, and allow it to cool (RA: bran = 10:1, m/m). Wine stir-fried: add RA in a 100 °C hot pot, stir-fry several times, spray well with wine, stir-fry dry, take out and cool (RA: wine = 20: 1, m/v). Salt stir-fry: take RA, add salt water to mix well, stuffy, place in the pot, heat with fire, fry dry, remove, cool (RA: salt = 50: 1, m/m). Soil stir-frying: heat the pot, take RA, place it in the pot, immediately scatter the soil powder into it, turn it evenly with an iron rake, stir until the soil powder is evenly adhered to the tablets, remove the sieve to remove the soil powder, and stay cool.

The view of TCM believes that different processing methods can change the medicinal properties of *R. alismatis*. It can promote water and dampness without damaging yin. Bran stir-fried *R. alismatis* has the characteristics of dampness, spleen, turbidity and clearing (Yan et al., 2020). Salt stir-fried *R. alismatis* has the effect of guiding medicine downward, enhancing yin-nourishing, heat-clearing and diuresis (Zhong et al., 2005). At present, the study on the functional differences of *R. alismatis* with different processing methods is still in the preliminary stage, and the effect of different processing methods on diuretic effect is still controversial (Figures 9B,C) (Wei et al., 2013; Yan et al., 2020; Zeng et al., 2011). In addition, the water extract of *R. alismatis* raw products and salt stir-fry products can alleviate pulmonary edema in edema mice (Zeng et al., 2011) and affect the anti-stress effect of mice. Taking water extract concentrate of raw products for 8–15 days will weaken the anti-fatigue, hypoxia tolerance and high temperature resistance of mice, while salt stir-fry *R. alismatis* can increase the anti-stress ability of mice (Wei et al., 2013). This may be related to the toxicity of *R. alismatis*, and salt disposing can neutralize some of the toxic metabolites. Xu et al. (Zhang et al., 2012) confirmed the stronger spleen-strengthening effect of bran stir-fry *R. alismatis* by detecting the content of serum gastrin h, the activity of Na^+^-K^+^-ATPase in rat duodenum and the contraction amplitude of isolated duodenal smooth muscle in rats. This finding aligns with the tonifying and beneficial effects of *R. alismatis* recorded in classical texts, thereby laying the groundwork for the development of its new applications.

The effective metabolites of *R. alismatis* are mainly prototerpenoid triterpenes, including Alisol A, Alisol B, Alisol C, etc. From the biological secondary metabolic pathway, they are derived from the high content of alisol B 23-acetate in fresh medicinal materials, and alisol A 24-acetate, alisol B, alisol A accounted for the vast majority. After processing, its chemical composition remains nearly unchanged, but the proportion of each substance varies. For instance, alisol B, alisolenol and epoxide alisolene in wine stir-fry *R. alismatis* and salt stir-fry *R. alismatis* did not change significantly compared with raw products; however, the contents of alisol B 23-acetate and 23-acetyl alisol C were significantly reduced, and the decrease rate was salt stir-fry > wine stir-fry > bran stir-fry > raw products. Additionally, the contents of alisol A and alisol A 24-acetate in wine stir-fry *R. alismatis* and salt stir-fry *R. alismatis* increased (Cao et al., 2016; Wei et al., 2021). However, the change of alisol B 23-acetate in bran stir-fry *R. alismatis* was controversial (Dai and Jia, 2008). Based on the existing literature, the change of diuretic effect caused by different processing methods is mainly attributed to the change of the proportion of triterpenoids. Tong et al. (Yan et al., 2023) compared and analyzed the blood-entering components in the serum of rats after taking row *R. alismatis* and salt stir-*R. alismatis* by ultra-high performance liquid chromatography-quadrupole-time-of-flight mass spectrometry (UPLC-Q-TOF- MS). It was found that the number of metabolites (5 prototype components and 9 metabolites) in the serum of rats given the water extract of salt stir-*R. alismatis* was lower than that of the raw water extract (5 prototype components and 15 metabolites), and the response intensity of the prototype metabolites 16-oxo alisol A, alisol B and alisol C in the serum of rats in the salt stir-fry *R. alismatis* group increased, while the types and response intensity of metabolic components generally decreased. It shows that the salt stir-*R. alismatis* may promote the absorption of prototype components by slowing down the metabolic rate of terpenoids.

## 5 Conclusion and discussion

With the innovation of detection technology, more and more chemical constituents have been found from *R. alismatis*, but most of them are extremely trace components. The detection of these new metabolites will not change our overall understanding of the function and toxicity. The content and ratio of bioactive components in *R. alismatis*, critical to its pharmacological profile, thus warrant focused attention. According to the development history of TCM, *R. alismatis*, the functions of diuresis-removing dampness, clearing heat and detoxifying, dissolving turbidity and lipid-lowing, which are recognized by traditional Chinese medicine. However, the tonic effect has been rarely mentioned, which may be related to the changes of material deficiency and nutrient deficiency in ancient times and the current material abundance and nutrient abundance. Modern pharmacological studies have shown that *R. alismatis* has diuretic, anti-kidney stone, anti-inflammation, hypoglycemic and lipid-lowering effects, which to a certain extent explains the effects of its TCM such as removing water and dampness, dissipating heat and removing fluids, and reducing turbidity and lipid-lowering. In addition, it also has liver protection and anti-tumor effects, but the latter is not verified *in vivo*. The Shennong Bencao Jing (Classic of Herbal Medicine) documents dermatological properties of *R. alismatis*—a pharmacological dimension rarely addressed in historical discourse and insufficiently investigated in contemporary research. This underexplored therapeutic potential carries significant implications for advancing phytotherapeutic innovations, particularly in dermatological applications.

One of the most controversial points of *R. alismatis* is the presence and size of its toxicity. Traditional Chinese medicine tends to regard *Rhizoma alismati* as a non-toxic medicinal material. However, modern pharmacological studies have found that it may cause some adverse reactions and poisoning events under certain circumstances. Triterpenoids are the main active ingredients in *R. alismatis*, for example, alisol B 23-acetate and alisol A 24-acetate have diuretic, lipid-lowering and liver-protecting effects (Meng et al., 2015; Wang et al., 2019; Zhang X. et al., 2017), but they can cause interstitial nephritis, apoptosis and exfoliation of renal tubular epithelial cells in rats at much higher doses and prolonged administration. These results suggest that triterpenoids are not only the effective components of *R. alismatis*, but also potential nephrotoxic substances. Alisol B 23-acetate and alisol A 24-acetate serve dual roles as both primary active constituents and principal toxic agents in *R. alismatis*. Intriguingly, some studies have linked their content to plucking time, processing and species (Chuan, Jian, and Guang *R. alismatis*). The alisol B 23-acetate content in *R. alismatis* harvested in April is significantly higher than in those harvested from January to March (Wen et al., 1998), which may be attributed to seasonal variations causing fluctuations in plant hormones. Studies have found that external application of salicylic acid, gibberellins, or abscisic acid can influence the alisol B 23-acetate content in *R. alismatis* (Li et al., 2015). Similarly, endogenous levels of plant hormones are affected by cultivation environments and climatic conditions, leading to regional variations. This partially explains the influence of species-specific differences on its chemical composition. The Chinese Pharmacopoeia mandates a minimum total content of 0.10% for alisol B 23-acetate and alisol C 23-acetate (two critical triterpenoid markers) in authenticated *R. alismatis* dry materials. However, the separate comparison of 24-acetylalismatiol A and 23-acetylalismatiol B could not distinguish *R. alismatis* from different origins. However, the inclusion of multiple variables (including alismoxide, alisol C 23-acetate, alisol A, alismol, alisol B, alisol B 23-acetate, 11-deoxyalisol B, 11-deoxyalisol C, alisol O, 24-acetylalisol A, and 11-deoxy-23-acetylalisol B) (Jin et al., 2020; Luo et al., 2010; Weng et al., 2022) enabled effective geographical differentiation through comprehensive analytical approaches. Specifically, hierarchical cluster analysis (HCA), principal component analysis (PCA), and Pearson correlation analysis collectively demonstrated significant discriminative power in characterizing regional variations.

To mitigate adverse effects of *R. alismatis*, diverse processing methods have been implemented. Salt-processing and bran-frying significantly alter the morphological characteristics of the botanical drug, while processing parameters (adjuvant concentration, temperature, duration) critically influence its bioactive constituent profiles (Yan et al., 2024). Processing of *R. alismatis* significantly reduces its adverse reactions compared to the crude drug. We hypothesize that the concentrations of its primary toxic components, alisol B 23-acetate and alisol A 24-acetate (as mentioned in Section 4.5), should be markedly decreased. However, existing literature presents conflicting results regarding this matter (Cai and Lin, 2021; Cao et al., 2016; Lu, 2013; Wei et al., 2021). This phenomenon suggests that: 1. Beyond the potential for undiscovered toxins in *R. alismatis*, our attention should shift to the combined levels of alisol B 23-acetate and alisol A 24-acetate; 2. The synergistic or antagonistic effects during processing may mitigate the adverse reactions. During this process, we also identified issues and challenges in the processing procedures. we observed inconsistent trends in the changes of bioactive components before and after processing, which could be attributed to variations in raw materials or a lack of standardization in the processing techniques. Numerous factors influence the content of these components, including the plant variety, cultivation region, climate, harvesting time, and processing methods. These complexities pose significant challenges to the standardized production of *Rhizome alismatis*. Herbal compatibility in traditional formulations may offer solutions. Preliminary evidence demonstrates reduced toxicity in compound preparations like Shenze Shugan Capsule (Dahuang, Juemingzi, Zexie) (Table 2), where *R. alismatis* appears to attenuate toxicity of co-administered botanical drugs (Dahuang and Juemingzi have always been considered to be highly toxic Chinese herbal medicines (Yang et al., 2024; Zhang et al., 2023)). In addition, toxicity experiments showed that even if 50–100 times the normal dose of long-term administration of *R. alismatis* water extract, there was no significant difference in behavioral activity, coat luster, mental state, drinking water and feces of rats. Liver, spleen, stomach, testis and other parts did not observed abnormalities expect for kidney. In the medium and high dose groups, the kidneys showed mild adverse effects, that is, the renal tubular epithelial cells showed swelling and degeneration and inflammatory cell infiltration in the renal interstitium (Duan et al., 2004). But severe adverse reactions were observed in the residual kidneys of partial nephrectomy mice (Zhu et al., 2007). In conclusion, *R. alismatis* is generally safe to take under the conventional dose. However, its toxicity depends on the metabolic capacity of the kidney, and patients with impaired renal function should be careful to take long-term high-doses. In addition, the production of safe and high-quality traditional Chinese medicine is inseparable from the source, that is, the high-quality provenances. There are 12 species of *R. alismatis* worldwide. At present, only two species, *Alisma plantago-aquatica* L. and *Alisma orientale* (Sam.) Juz., are included in the Chinese Pharmacopoeia, and the whole traceability system has not been established for planting and circulation. With the accelerating internationalization of TCM, it is suggested to carry out systematic evaluation of “Chinese medicinal properties” and quality of various varieties of *R. alismatis*, screen high-quality varieties, and establish a standardized cultivation, processing and circulation system, so as to provide a guarantee for the production of high-quality Chinese medicine *R. alismatis* and ensure the clinical efficacy of TCM.
