# Supplementary material for: Botany, traditional uses, phytochemistry, pharmacology, toxicology and processing of Rhizoma alismatis: a review
Source: Front Pharmacol. 2025 Dec 4;16:1722483. doi: 10.3389/fphar.2025.1722483 (PMC12712712; doi:10.3389/fphar.2025.1722483)
Supplement: Supplementary file 1 [file Table1.docx]

Table S1. The formulation, functions and precautions of Chinese patent medicine of *Rhizoma alismatis*.

| Name | Prescription | Functions | Applicable Population | Adverse reactions /precautions |
| --- | --- | --- | --- | --- |
| Longqing Tablet/capsule | ***Rhizoma alismatis* (Zexie)**, *Plantago asiatica* L. (Cheqianzi), *Patrinia villosa* Juss. (Baijiangcao), *Lonicera japonica* Thunb. (Jinyinhua), *Paeonia × suffruticosa* Andrews (Mudanpi), *Oldenlandia diffusa* (Willd.) Roxb. (Baihuasheshecao), *Paeonia lactiflora* Pall. (Chishao), *Agrimonia pilosa* Ledeb. (Xianhecao), *Coptis chinensis* Franch. (Huanglian), *Phellodendron chinense* C.K.Schneid. (Huangbai) | Clearing heat,  detoxifying,  cooling blood,  removing dampness,  promoting diuresis. | It is used for damp-heat in lower jiao. The symptoms include frequent urination, urgency, dysuria, short urination, lumbago and abdominal distention. | Body deficiency stomach cold should not be taken. |
| Xuezhiling Tablet | ***Rhizoma alismatis* (Zexie)**, *Cassia obtusifolia* L. (Juemingzi), *Crataegus pinnatifida* Bunge (Shanzha), *Polygonum multiflorum* Thunb. (Zhiheshouwu) | Turbidity lipid-lowering, laxative. | It is used for phlegm turbidity block type hyperlipidemia.  The symptoms are dizziness, chest tightness and dry stool. | / |
| Wuling Capsule/Powder | ***Rhizoma alismatis* (Zexie)**, *Poria cocos (Schw.)* Wolf (Fuling), *Polyporus umbellatus (Pers.)* Fries (Zhuling), *Cinnamomum cassia (L.)* J.Presl (Rougui), *Atractylodes macrocephala* Koidz. (Baizhu) | Warm yang,  damp elimination,  promoting diuresis. | It is used for bladder gas adverse, water wet cohesion caused by urine adverse, edema, abdominal distension, vomiting and diarrhea, not drinking. | / |
