# Supplementary figures and images for: Botany, traditional uses, phytochemistry, pharmacology, toxicology and processing of Rhizoma alismatis: a review
Source: Front Pharmacol. 2025 Dec 4;16:1722483. doi: 10.3389/fphar.2025.1722483 (PMC12712712; doi:10.3389/fphar.2025.1722483)

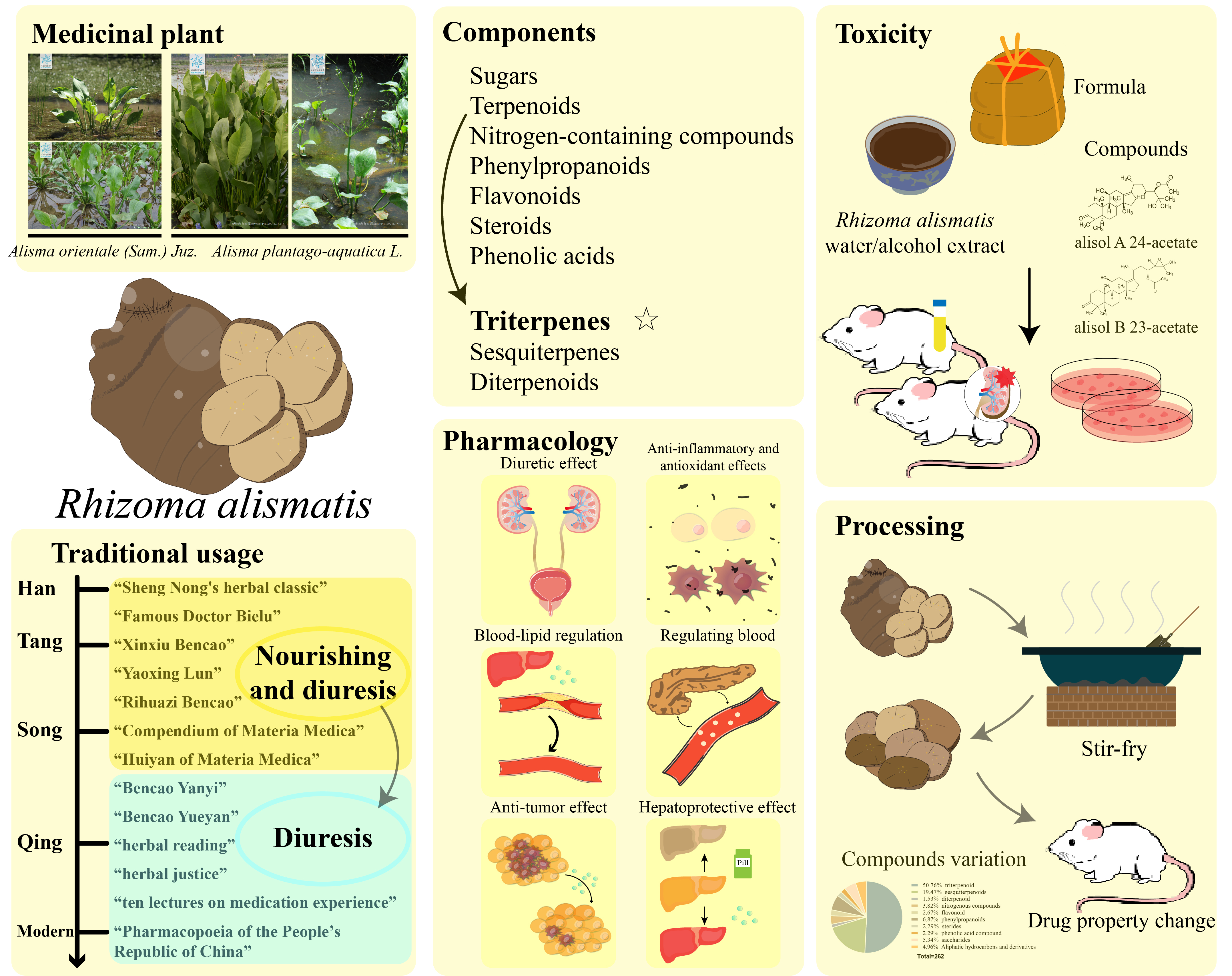

Supplement: Supplementary file 2 [file Image1.jpeg]

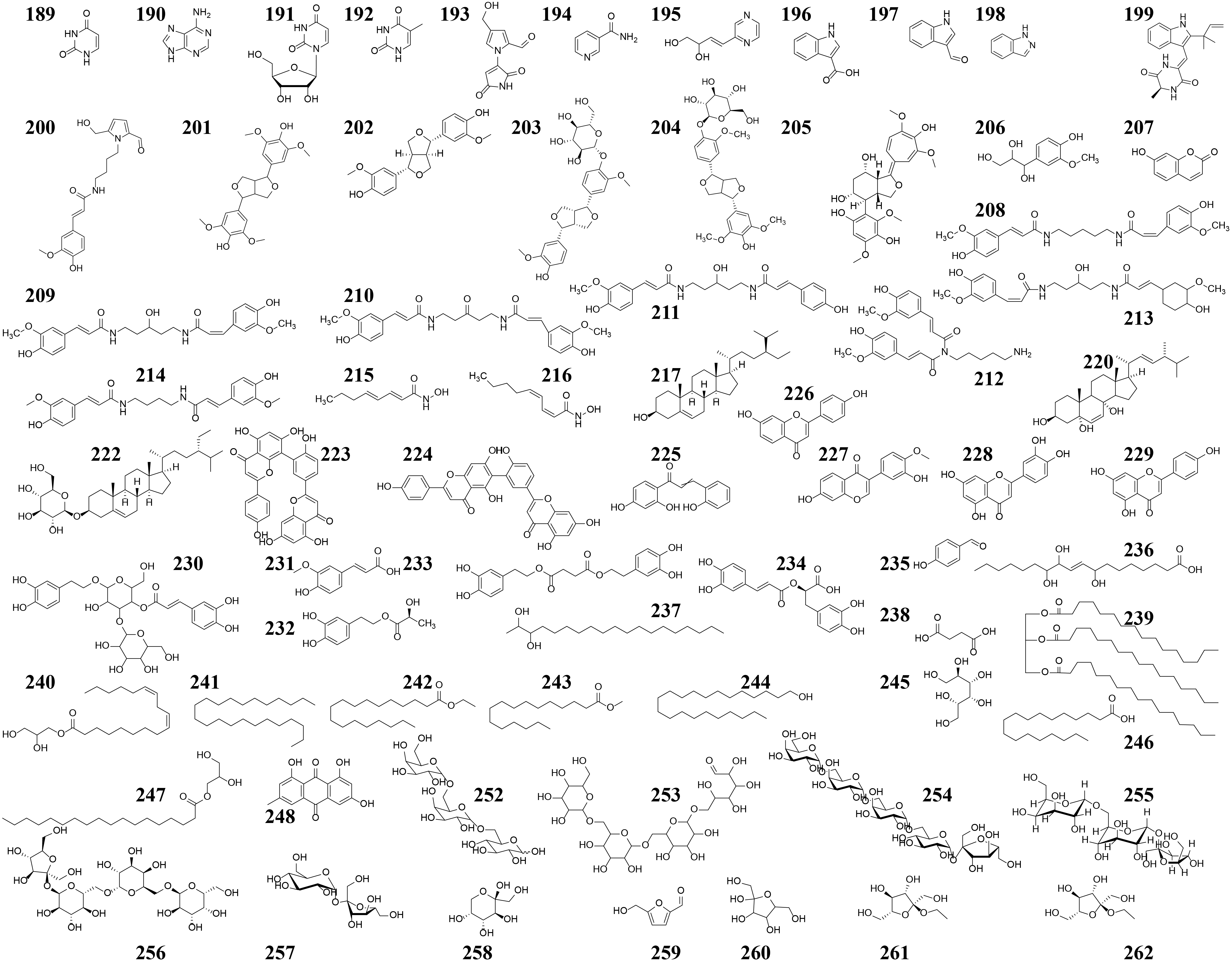

Supplement: Supplementary file 3 [file Image2.jpeg]
