# Supplementary material for: Botany, traditional uses, phytochemistry, pharmacology, toxicology and processing of Rhizoma alismatis: a review
Source: Front Pharmacol. 2025 Dec 4;16:1722483. doi: 10.3389/fphar.2025.1722483 (PMC12712712; doi:10.3389/fphar.2025.1722483)
Supplement: Supplementary file 4 [file Table2.docx]

Table S2. Terpenoids in *Rhizoma alismatis*

| **NO.** | **Name** | **Nucleus** | **R** | **R_1_** | **R_2_** | **R_3_** | **R_4_** | **Part of plant** | **Identification methods** | **Categories** | **Refs.** |
| --- | --- | --- | --- | --- | --- | --- | --- | --- | --- | --- | --- |
| 1. | alisol A | A | *β-*OH | H | *β-*OH | *β*-OH | OH | rhizome; stems; leaves | mp; [α]D; MS; EA; IR; TLC;CC; NMR | Terpenoids | (Murata et al., 1970; Nakajima et al., 1994) |
| 2. | alisol A 23-acetate | A | *β-*OH | H | *β-*OAc | *β*-OH | OH | rhizome | mp; [α]D; MS; EA; IR; TLC;CC;acetylation/hydrolysis | Terpenoids | (Murata et al., 1970) |
| 3. | alisol A 24-acetate | A | *β-*OH | H | *β-*OH | *β*-OAc | OH | rhizome | NMR; MS; IR; [α]D; TLC; CC | Terpenoids | (Nakajima et al., 1994) |
| 4. | 25-obutyl alisol A | A | *β-*OH | OCH_3_ | *β-*OH | *β*-OH | OCH_3_ | rhizome | HRESIMS; 1D & 2D NMR (¹H;¹³C; HMBC; COSY); [α]D; UV | Terpenoids | (Zhang et al., 2017) |
| 5. | 11-deoxy alisol A | A | *β-*H | H | *β-*OH | *β-*OH | OH | rhizome | NMR; MS; IR; [α]D; TLC; CC | Terpenoids | (Nakajima et al., 1994) |
| 6. | 25-O-methyl alisol A | A | *β-*OH | H | *β-*OH | *β-*OH | OCH_3_ | rhizome | NMR; MS; IR; [α]D; TLC; CC | Terpenoids | (Nakajima et al., 1994) |
| 7. | 25-O-ethyl alisol A | A | *β-*OH | H | *β-*OH | *β-*OH | OCH_2_CH_3_ | rhizome | [α]D; UV; HRESIMS; 1D/2D NMR; IECD | Terpenoids | (Mai et al., 2015) |
| 8. | 15, 16-dihydro alisol A | A | *β-*OH | H | *β-*OH | OH | OH | rhizome | [α]D; UV; HRESIMS; 1D/2D NMR | Terpenoids | (Mai et al., 2015) |
| 9. | alisol E | A | *β-*OH | H | *β-*OH | *β-*OH | OH | rhizome | mp; [α]D; IR; FAB-MS;HR-MS; ^1^H-NMR; ^13^C-NMR | Terpenoids | (Yoshikawa, Hatakeyama, et al., 1993) |
| 10. | alisol E 23-acetate | A | *β-*OH | H | *β-*OCOCH_3_ | *β-*OH | OH | rhizome | mp; [α]D; IR; FAB-MS;HR-MS; ^1^H-NMR; ^13^C-NMR; | Terpenoids | (Yoshikawa, Hatakeyama, et al., 1993; Yoshikawa et al., 1997) |
| 11. | alisol E 24-acetate | A | *β-*OH | H | *β-*OH | *β-*OCOCH_3_ | OH | rhizome | mp; IR; ESI-MS;  ^1^H-NMR; ^13^C-NMR | Terpenoids | (Peng et al., 2002a) |
| 12. | 16β-methoxy alisol E | A | *β-*OH | *β-*OCH_3_ | *β-*OH | *α-*OH | OH | rhizome | HR-ESI-MS; 1D & 2D NMR (¹H; ¹³C; HMBC; ROESY); IR; UV; [α]D | Terpenoids | (H. Li, X. Chen, et al., 2017) |
| 13. | 16β, 25-dimethoxy alisol E | A | *β-*OH | *β-*OCH_3_ | *β-*OH | *α-*OH | OCH_3_ | rhizome | HR-ESI-MS; 1D & 2D NMR (¹H; ¹³C; HMBC); IR; UV; [α]D | Terpenoids | (Li et al., 2017) |
| 14. | 16β-hydroperoxy alisol E | A | *β-*OH | *β-*OOH | *β-*OH | *α-*OH | OH | rhizome | HR-ESI-MS; 1D & 2D NMR (¹H; ¹³C; HMBC); IR; UV; [α]D | Terpenoids | (Li et al., 2017) |
| 15. | alisol H | A | *β-*H | =O | =O | H | H | rhizome | ^1^H-NMR; ^13^C-NMR; | Terpenoids | (Yoshikawa et al., 1997; Yoshikawa et al., 1999) |
| 16. | 11, 24-dihydlroxy-alsol H | A | *β-*OH | =O | =O | *β-*OH | H | rhizome | ESI-MS; (¹H-NMR; ¹³C;-NMR; HMQC; ¹H-¹H-COSY; HMBC | Terpenoids | (Li, 2013) |
| 17. | 3-oxo-11β, 23-dihydroxy-24, 24-dimethyl-26, 27-dinorprotost-13(17)-en-25-oic acid | A | *β-*OH | =O | *β-*H | OH | COOH | rhizome | ^1^H-NMR; ^13^C-NMR; | Terpenoids | (Zhao et al., 2008) |
| 18. | alisol T | A | *β-*OH | *β-*OCH_3_ | =O | H | OH | rhizome | HR-ESI-MS; 1D & 2D NMR (¹H; ¹³C; HMBC); IR; UV; [α]D | Terpenoids | (Li et al., 2017) |
| 19. | alismanin I | A | *β-*OH | H | =O | *β-*OH | H | Rhizome | HRESIMS ; NMR Spectroscopy; UV | Terpenoids | (Yi et al., 2019) |
| 20. | 11-deoxy-16-oxo-alisol A | A | *β-*H | =O | *β-*OH | *β-*OH | OH | Rhizome | HPLC-DAD-Q-TOF MS | Terpenoids | (Zhao et al., 2015) |
| 21. | 5β, 29-dihydroxy alisol A | B | OH | OH | H | OH | H | Rhizome | HRESIMS;1D NMR; 2D NMR: | Terpenoids | (Wang et al., 2017) |
| 22. | 7α-hydroxy alisol A | B | H | H | *α*-OH | OH | H | biotransformation product | HRESIMS;1D&2D NMR (¹H;¹³C; HMBC; COSY;NOESY);[α]D; UV | Terpenoids | (Zhang et al., 2017) |
| 23. | 7β-hydroxy alisol A | B | H | H | *β*-OH | *β-*OH | H | biotransformation product | HRESIMS;1D&2D NMR (¹H;¹³C; HMBC; COSY;NOESY);[α]D; UV | Terpenoids | (Zhang et al., 2017) |
| 24. | 7α, 12α-dihydroxy alisol A | B | H | H | *α*-OH | *β*-OH | *α*-OH | biotransformation product | HRESIMS;1D&2D NMR (¹H;¹³C; HMBC; COSY;NOESY);[α]D; UV | Terpenoids | (Zhang et al., 2017) |
| 25. | 7α-hydroxy-24-oxo alisol A | C | H | *β*-OH | H | *H* | / | biotransformation product | HRESIMS;1D&2D NMR(¹H; ¹³C; HMBC; NOESY); [α]D; UV | Terpenoids | (Zhang et al., 2017) |
| 26. | 7α, 12α-dihydroxy-24-oxo alisol A | C | *α*-OH | *β*-OH | *α*-OH | H | / | biotransformation product | HRESIMS;1D&2D NMR(¹H; ¹³C; HMBC; NOESY); [α]D; UV | Terpenoids | (Zhang et al., 2017) |
| 27. | dehyro-16-oxo-alisolA | C | H | CH_3_ | H | =O | / | rhizome | HPLC/Q-TOF-MS | Terpenoids | (Song et al., 2013) |
| 28. | 11-deoxy-13β,17β-epoxy alisol A | D | H | H | / | / | / | rhizome | NMR; MS; IR; [α]D; TLC; CC | Terpenoids | (Nakajima et al., 1994) |
| 29. | 13β, 17β-epoxy alisol A | D | *β-*OH | H | / | / | / | rhizome | NMR; MS; IR; [α]D; TLC; CC | Terpenoids | (Nakajima et al., 1994) |
| 30. | 13β, 17β-epoxy alisol A 24-acetate | D | *β-*OH | Ac | / | / | / | rhizome | ¹H-NMR;  ¹³C-NMR; DEPT; ¹H-¹H COSY; HSQC; HMB | Terpenoids | (Peng & Lou, 2002) |
| 31. | 16-oxo alisol A 23--acetate | E | OH | H | *β-*OCOCH_3_ | *β-*OH | / | rhizome | HPLC-DAD-Q-TOF MS;UPLC-QqQ MS | Terpenoids | (Zhao et al., 2015) |
| 32. | 16-oxo alisol A 24--acetate | E | OH | H | *β-*OH | *β-*OCOCH_3_ | / | rhizome | HPLC-DAD-Q-TOF MS;UPLC-QqQ MS | Terpenoids | (Zhao et al., 2015) |
| 33. | 12-hydroxy-16-oxo-alisol A 24 acetate | E | OH | OH | *β-*OH | *β-*OAc | / | rhizome | HR-ESI-MS; ¹H-NMR;  ¹³C-NMR; UHPLC-QTOF-MS/MS; UHPLC-QTRAP-MS/MS | Terpenoids | (Li et al., 2017) |
| 34. | 16-oxo-11-anhydro alisol A | F | =O | *β*-OH | *β*-OH | / | / | rhizome | ^1^H-NMR; ^13^C-NMR;MS | Terpenoids | (Mai et al., 2015) |
| 35. | 16-oxo-11-anhydro alisol A | F | H | OH | *β-*OH | / | / | rhizome | ^1^H-NMR; ^13^C-NMR;MS | Terpenoids | (Mai et al., 2015) |
| 36. | 16-oxo-11-anhydro alisol A 24 acetate | F | H | OH | *β-*OAc | / | / | rhizome | HRESIMS; IR; ¹H-NMR;  ¹³C-NMR;  HSQC; HMBC | Terpenoids | (Ma et al., 2016) |
| 37. | alismanol A | F | H | =O | *α-*OH | / | / | rhizome | [α]D; UV; HRESIMS; 1D/2D NMR (^1^H; ^13^C; COSY; HSQC; HMBC; NOESY); IECD | Terpenoids | (Mai et al., 2015) |
| 38. | alismanol C | F | H | OH | *α-*OH | / | / | rhizome | [α]D; UV; HRESIMS; 1D/2D NMR | Terpenoids | (Mai et al., 2015) |
| 39. | 16-oxo alisol A | G | OH | OH | *β-*OAc | / | / | rhizome | NMR; MS; IR; [α]D; TLC; CC | Terpenoids | (Nakajima et al., 1994) |
| 40. | 25-anhydroalisol A 11-acetate | G | OAc | OH | *β-*OH | / | / | rhizome | ¹H-NMR;  ¹³C-NMR; | Terpenoids | (Peng et al., 2002) |
| 41. | 25-anhydroalisol A 24-acetate | G | OH | OH | *β-*OAc | / | / | rhizome | IR; ESI-MS;¹H-NMR;  ¹³C-NMR | Terpenoids | (Peng et al., 2002) |
| 42. | 11-deoxy-25-anhydro alisol E | G | H | *β-*OH | *α-*OH | / | / | rhizome | [α]D; UV; HRESIMS; 1D/2D NMR; IECD | Terpenoids | (Mai et al., 2015) |
| 43. | 23-acetate-25-anhydroslisol E | G | OH | H | *α-*OCOCH_3_ | / | / | rhizome | HPLC | Terpenoids | (Han et al., 2013) |
| 44. | 24-acetate-25-anhydroslisol E | G | OH | *β-*OCOCH_3_ | H | / | / | rhizome | HPLC | Terpenoids | (Han et al., 2013) |
| 45. | alisol G | G | OH | OH | *β-*OH | / | / | rhizome | [α]D; IR; FAB-MS; HR-MS; ^1^H-NMR; ^13^ C-NMR; 2D NMR (^1^H-^13^C COSY; NOESY) | Terpenoids | (Yoshikawa, Hatakeyama, et al., 1993) |
| 46. | alisol G 23-acetate | G | OH | OAc | *β-*OH | / | / | rhizome | ^1^H-NMR; ^13^C-NMR;MS | Terpenoids | (Yoshikawa et al., 1997) |
| 47. | alisol X | G | OH | H | =O | / | / | rhizome | HPLC; ESI-MS; HR-MS;IR; ^1^H-NMR; ^13^ C-NMR;  HSQC; HMBC | Terpenoids | (Xu et al., 2012) |
| 48. | alisol B | H | OH | H | *β-*OH | / | / | rhizome; stems; leaves | mp; [α]D; MS; EA; IR; TLC;CC;Liebermann–Burchard reaction; NMR | Terpenoids | (Murata et al., 1970; Nakajima et al., 1994) |
| 49. | alisol B 11-monoacetate | H | *β-*OCOCH_3_ | H | *β-*OH | / | / | rhizome | NMR; MS; IR; [α]D; TLC; CC | Terpenoids | (Nakajima et al., 1994) |
| 50. | alisol B 23-acetate | H | OH | H | *β-*OAc | / | / | rhizome | mp; [α]D; MS; EA; IR; TLC; CC;NMR | Terpenoids | (Murata et al., 1970; Nakajima et al., 1994) |
| 51. | 11-deoxyalisol B | H | H | H | *β-*OH | / | / | rhizome | NMR; MS; IR; [α]D; TLC; CC | Terpenoids | (Nakajima et al., 1994) |
| 52. | 11-deoxyalisol B 23-acetate | H | H | H | *β-*OAc | / | / | rhizome | NMR; MS; IR; [α]D; TLC; CC | Terpenoids | (Nakajima et al., 1994) |
| 53. | 16β-acetoxy alisol B | H | OH | *β-*OAc | *α*-OH | / | / | rhizome | HRESIMS; UV;  ^1^H-NMR; ^13^ C-NMR;  HMBC; NOESY | Terpenoids | (Cang et al., 2017a) |
| 54. | 16α-acetoxy alisol B | H | OH | *α*- OAc | *α*-OH | / | / | rhizome | HRESIMS; UV;  ^1^H-NMR; ^13^ C-NMR;  HMBC; NOESY | Terpenoids | (Cang et al., 2017a) |
| 55. | 16β-hydroxy-alisol B 23-acetate | H | OH | OH | *β-*OAc | / | / | rhizome | mp; [α]D; MS; EA; IR; TLC;CC;acetylation/hydrolysis; NMR | Terpenoids | (Murata et al., 1970; Nakajima et al., 1994) |
| 56. | 16β-hydroperoxy alisol B | H | OH | *β-*OOH | *β-*OH | / | / | rhizome | HR-ESI-MS; 1D & 2D NMR (¹H; ¹³C; HMBC; ROESY); UV; [α]D | Terpenoids | (Li et al., 2017) |
| 57. | 16β-hydroperoxy alisol B 23-acetate | H | OH | *β-*OOH | *β-*OAc | / | / | rhizome | HR-ESI-MS; 1D & 2D NMR (¹H; ¹³C; HMBC; ROESY); IR; UV; [α]D; X-ray diffraction | Terpenoids | (Li et al., 2017) |
| 58. | 16β-methoxy alisol B | H | OH | *β-*OCH_3_ | *β-*OH | / | / | rhizome | HR-ESI-MS; 1D & 2D NMR (¹H; ¹³C; HMBC; ROESY); IR; UV; [α]D | Terpenoids | (Li et al., 2017) |
| 59. | 16β-methoxy alisol B 23-acetate | H | OH | *β-*OCH_3_ | *β-*OAc | / | / | rhizome | MS; IR; ^1^H-NMR; ^13^ C-NMR;  acetylation; NOE | Terpenoids | (Wu, et al., 1988) |
| 60. | 16β-methoxy alisol B monoacetate | H | *β-*OH | *β-*OCH_3_ | *β-*OCOCH_3_ | / | / | rhizome | MS; IR; ^1^H-NMR; ^13^ C-NMR;  acetylation; NOE | Terpenoids | (Wu et al., 1988) |
| 61. | 16β-ethoxy alisol B 23-acetate | H | *β-*OH | / | / | / | / | rhizome | HRESIMS;1D&2D NMR(¹H; ¹³C; HMBC; NOESY); [α]D; UV | Terpenoids | (Zhang et al., 2017) |
| 62. | 16-methyoxy-alisol 23-acetate | H | H | OCH_3_ | *β-*OCOCH_3_ | / | / | rhizome | NMR;MS;HPLC | Terpenoids | (Tian et al., 2014) |
| 63. | 11-deoxy-13β,17β-epoxyalisol B | I | H | *β-*OH | / | / | / | rhizome | NMR; MS; IR; [α]D; TLC; CC | Terpenoids | (Nakajima et al., 1994; Yoshikawa et al., 1999) |
| 64. | 11-deoxy-13β,17β-epoxyalisol B 23-acetate | I | H | *β-*OAc | / | / | / | rhizome | NMR; MS; IR; [α]D; TLC; CC | Terpenoids | (Nakajima et al., 1994; Yoshikawa et al., 1999) |
| 65. | 13β,17β-epoxy alisol B | I | OH | *β-*OH | / | / | / | rhizome | NMR; MS; IR; [α]D; TLC; CC | Terpenoids | (Nakajima et al., 1994) |
| 66. | alisol D | I | OH | *β-*OAc | / | / | / | rhizome | NMR; MS; IR; [α]D; TLC; CC | Terpenoids | (Fukuyama et al., 2007; Nakajima et al., 1994) |
| 67. | alisol D acetate | I | *β-*OCOCH_3_ | *β-*OCOCH_3_ | / | / | / | rhizome | IR;EI-MS;^1^H-NMR; ^13^ C-NMR; | Terpenoids | (Fukuyama et al., 2007) |
| 68. | 11-deoxyalisol D | I | H | *β-*OH | / | / | / | rhizome | ^1^H-NMR; ^13^C-NMR;MS | Terpenoids | (Yoshikawa et al., 1999) |
| 69. | alisol C | J | OH | H | H | / | / | rhizome | NMR; MS; IR; [α]D; TLC; CC | Terpenoids | (Nakajima et al., 1994) |
| 70. | alisol C 23-acetate | J | OH | H | Ac | / | / | rhizome | mp; [α]D; MS; EA; IR; TLC; CC;NMR | Terpenoids | (Murata et al., 1970; Nakajima et al., 1994; Yoshikawa et al., 1997) |
| 71. | 11-deoxy alisol C | J | H | H | H | / | / | rhizome | UV; IR;EI-MS;^1^H-NMR; ^13^ C-NMR; | Terpenoids | (Fukuyama et al., 2007) |
| 72. | 11-deoxy alisol C 23-acetate | J | H | H | Ac | / | / | rhizome | NMR; MS; IR; [α]D; TLC; CC | Terpenoids | (Nakajima et al., 1994) |
| 73. | 20-hydroxy alisol C | J | OH | OH | OH | / | / | rhizome | [α]D; UV; HRESIMS; 1D/2D NMR | Terpenoids | (Mai et al., 2015) |
| 74. | alisol F | K | *β-*OH | OH | / | / | / | rhizome | [α]D; IR; FAB-MS; HR-MS; ^1^H-NMR; ^13^ C-NMR; 2D NMR (^1^H-^13^C COSY; NOESY) | Terpenoids | (Yoshikawa, Hatakeyama, et al., 1993; Yoshikawa et al., 1997) |
| 75. | alisol F diacetate | K | *β-*OAc | OAc | / | / | / | rhizome | NMR;MS;HPLC | Terpenoids | (Tian et al., 2014) |
| 76. | alisol F 24-acetate | K | *β-*OAc | OH | / | / | / | Tuber | TLC;IR;EI-MS;^1^H-NMR; ^13^ C-NMR;  H-H COSY; ^13^ C-H COSY | Terpenoids | (Peng & Lou, 2001) |
| 77. | 25-methoxy alisol F | K | *β-*OH | OCH_3_ | / | / | / | rhizome | HR-ESI-MS; 1D & 2D NMR (¹H; ¹³C; ROESY); IR; UV; [α]D; X-ray diffraction | Terpenoids | (Li et al., 2017) |
| 78. | 16, 23-oxido alisol B | L | OH | / | / | / | / | rhizome | NMR; MS; IR; [α]D; TLC; CC | Terpenoids | (Murata et al., 1970; Nakajima et al., 1994) |
| 79. | alisol I | L | H | / | / | / | / | rhizome | ^1^H-NMR; ^13^C-NMR;MS | Terpenoids | (Yoshikawa et al., 1997; Yoshikawa et al., 1999) |
| 80. | alisol J 23-acetate | M | *α-*O | / | / | / | / | rhizome | ^1^H-NMR; ^13^C-NMR;MS | Terpenoids | (Yoshikawa et al., 1999) |
| 81. | alisol K 23-acetate | M | *β-*O | / | / | / | / | rhizome | ^1^H-NMR; ^13^C-NMR;MS | Terpenoids | (Yoshikawa et al., 1999) |
| 82. | alisol M 23-acetate | N | =O | / | / | / | / | rhizome | ^1^H-NMR; ^13^C-NMR;MS | Terpenoids | (Yoshikawa et al., 1999) |
| 83. | alisol N 23-acetate | N | H | / | / | / | / | rhizome | ^1^H-NMR; ^13^C-NMR;MS | Terpenoids | (Yoshikawa et al., 1999) |
| 84. | alisol L | O | OH | / | / | / | / | rhizome | HPLC-DAD-Q-TOF MS;UPLC-QqQ MS | Terpenoids | (Zhao et al., 2015) |
| 85. | alisol L 23-acetate | O | OAc | / | / | / | / | rhizome | ^1^H-NMR; ^13^C-NMR;MS | Terpenoids | (Yoshikawa et al., 1999) |
| 86. | alisol O | P | H | *β-*OAc | OH | / | / | rhizome | HRESIMS; 1D/2D NMR | Terpenoids | (Jiang et al., 2006; Zhou et al., 2008) |
| 87. | 24-deacetyl alisol O | P | H | *β-*OH | OH | / | / | rhizome | HRESIMS; 1D/2D NMR | Terpenoids | (Jiang et al., 2006; Zhou et al., 2008) |
| 88. | 16S,24S-dihydroxy-24-deacetyl-alisol O | P | *α-*OH | *α-*OH | OH | / | / | rhizome | HR-ESI-MS; NMR (¹H; ¹³C; HSQC; HMBC; ¹H-¹H COSY); IR; UV; CD | Terpenoids | (Liu et al., 2019) |
| 89. | alisol Q 23-acetate | Q | CH_2_CH_3_ | / | / | / | / | rhizome | HR-TOF-MS; IR; 1D/2D NMR (¹H; ¹³C; COSY; HSQC; HMBC; NOESY) | Terpenoids | (Jin et al., 2012) |
| 90. | alisol S 23-acetate | Q | Ac | / | / | / | / | rhizome | HR-ESI-MS; 1D & 2D NMR (¹H; ¹³C; COSY; HMBC; ROESY); IR; UV; [α]D | Terpenoids | (Li et al., 2017) |
| 91. | alisolide G | Q | COCH_3_ | / | / | / | / | rhizome | HR-ESI-MS; IR; UV; [α]D; 1D/2D NMR (¹H; ¹³C; COSY; HSQC; HMBC; NOESY) | Terpenoids | (Jin et al., 2019) |
| 92. | 3-methyl alismalactone 23-acetate | R | H | / | / | / | / | rhizome | [α]D; IR; FAB-MS; ^1^ H-NMR; ^13^C-NMR; 2D NMR (H-H COSY; HMBC; NOESY) | Terpenoids | (Yoshikawa et al., 1997) |
| 93. | alismaketone A 23-acetate | R | CH_3_ | / | / | / | / | rhizome | MS; IR; [α]D; 1D/2D NMR (¹H; ¹³C; COSY; HMBC) | Terpenoids | (Matsuda et al., 1999) |
| 94. | alismanol D | S | *α-*OH | / | / | / | / | rhizome | [α]D; UV; HRESIMS; 1D/2D NMR; IECD | Terpenoids | (Mai et al., 2015) |
| 95. | 24-epi alismanol D | S | *β-*OH | / | / | / | / | rhizome | HRESIMS; UV; [α]D; 1D/2D NMR (¹H; ¹³C; HSQC; HMBC) | Terpenoids | (Zhao, et al., 2017) |
| 96. | alismanol O | T | H | / | / | / | / | rhizome | HRESIMS; UV; [α]D; 1D/2D NMR (¹H; ¹³C; HSQC; HMBC;¹H -¹H COSY; NOESY) | Terpenoids | (Xin et al., 2016) |
| 97. | alismanol P | T | OH | / | / | / | / | rhizome | HRESIMS; UV; [α]D; 1D/2D NMR (¹H; ¹³C; HSQC; HMBC;¹H -¹H COSY; NOESY) | Terpenoids | (Xin et al., 2016) |
| 98. | neoalisol | U | H | H | / | / | / | rhizome | ¹H-NMR;  ¹³C-NMR | Terpenoids | (Peng et al., 2002b) |
| 99. | neoalisol 11,24-acetate | U | Ac | Ac | / | / | / | rhizome | ¹H-NMR;  ¹³C-NMR | Terpenoids | (Peng et al., 2002b) |
| 100. | alisolide A | V | =O | OH | / | / | / | rhizome | HR-ESI-MS; IR; UV; 1D/2D NMR (¹H; ¹³C; COSY; HSQC; HMBC; NOESY) | Terpenoids | (Jin et al., 2019) |
| 101. | alisolide B | V | =O | OOH | / | / | / | rhizome | HR-ESI-MS; IR; UV; 1D/2D NMR (¹H; ¹³C; COSY; HSQC; HMBC; NOESY) | Terpenoids | (Jin et al., 2019) |
| 102. | alisolide C | V | *β-*OH | *β-*OH | / | / | / | rhizome | HR-ESI-MS; IR; UV; 1D/2D NMR (¹H; ¹³C; COSY; HSQC; HMBC; NOESY) | Terpenoids | (Jin et al., 2019) |
| 103. | 25-anhydro-alisol F | W | OH | / | / | / | / | rhizome | ¹H-NMR;¹³C-NMR;MS; ESI-MS; IR;  HMQC; ¹H–¹H COSY; NOESY | Terpenoids | (Hu et al., 2008; Ma et al., 2016) |
| 104. | 11, 25-anhydro-alisol F | W | H | / | / | / | / | rhizome | ESI-MS; HR-ESI-MS; IR; ¹H-NMR;¹³C-NMR; HMBC;¹H–¹H COSY; NOESY | Terpenoids | (Hu et al., 2008) |
| 105. | 13β, 17β-epoxy-24, 25, 26, 27-tetranor-alisol A 23-oic acid |  | / | / | / | / | / | rhizome | HRESIMS; IR; ¹H-NMR;  ¹³C-NMR;  HSQC; HMBC | Terpenoids | (Ma et al., 2016) |
| 106. | alisol P |  | / | / | / | / | / | rhizome | ¹H-NMR;¹³C-NMR | Terpenoids | (Zhao et al., 2008) |
| 107. | alisol R |  | / | / | / | / | / | rhizome | HR-ESI-MS; 1D & 2D NMR (¹H; ¹³C; COSY; HMBC; ROESY); IR; UV; [α]D | Terpenoids | (Li et al., 2017) |
| 108. | alisol U |  | / | / | / | / | / | rhizome | HR-ESI-MS; IR; UV; | Terpenoids | (Li et al., 2017) |
| 109. | alisol V |  | / | / | / | / | / | rhizome | HR-ESI-MS; IR; UV; | Terpenoids | (Li et al., 2017) |
| 110. | alisol W |  | / | / | / | / | / | rhizome | HR-ESI-MS; IR; UV;  ¹H-NMR;¹³C-NMR;HMBC;¹H–¹H COSY; ROESY | Terpenoids | (Li et al., 2018) |
| 111. | alisolide |  | / | / | / | / | / | rhizome | ¹H-NMR;¹³C-NMR | Terpenoids | (Zhao et al., 2008) |
| 112. | alisolide H |  | / | / | / | / | / | rhizome | HR-ESI-MS; IR; UV; [α]D; Mo₂(OAc)₄-ECD; 1D/2D NMR; ESI-MSⁿ | Terpenoids | (Jin et al., 2019) |
| 113. | alisolide I |  | / | / | / | / | / | rhizome | HR-ESI-MS; IR; UV; [α]D; 1D/2D NMR (¹H; ¹³C; COSY; HSQC; HMBC; NOESY) | Terpenoids | (Jin et al., 2019) |
| 114. | 17-epialisolide A |  | / | / | / | / | / | rhizome | HRESIMS; UV; [α]D; 1D/2D NMR (¹H; ¹³C; HSQC; HMBC) | Terpenoids | (Zhao et al., 2017) |
| 115. | alismaketone A 23-acetate |  | / | / | / | / | / | rhizome | [α]D; CD; IR; FAB-MS; ^1^ H-NMR; ^13^C-NMR; 2D NMR (H-H COSY; HMBC) | Terpenoids | (Yoshikawa et al., 1997) |
| 116. | alismaketone B 23-acetate |  | / | / | / | / | / | rhizome | FAB-MS; IR; [α]D; 1D/2D NMR (¹H; ¹³C; COSY; HMBC) | Terpenoids | (Matsuda et al., 1999) |
| 117. | alismaketone C 23-acetate |  | / | / | / | / | / | rhizome | FAB-MS; IR; [α]D; 1D/2D NMR (¹H; ¹³C; COSY; HMBC) | Terpenoids | (Matsuda et al., 1999) |
| 118. | alismanol B |  | / | / | / | / | / | rhizome | [α]D; UV; HRESIMS; 1D/2D NMR; IECD | Terpenoids | (Mai et al., 2015) |
| 119. | alismanol E |  | / | / | / | / | / | rhizome | [α]D; UV; HRESIMS; 1D/2D NMR | Terpenoids | (Mai et al., 2015) |
| 120. | alismanol F |  | / | / | / | / | / | rhizome | [α]D; UV; HRESIMS; 1D/2D NMR | Terpenoids | (Mai et al., 2015) |
| 121. | alismanol I |  | / | / | / | / | / | rhizome | HRESIMS;1D&2D NMR (¹H;¹³C; HMBC; NOESY); [α]D; UV | Terpenoids | (Zhang et al., 2017) |
| 122. | alismanol J |  | / | / | / | / | / | rhizome | HRESIMS;1D&2D NMR (¹H;¹³C; HMBC; NOESY); [α]D; UV | Terpenoids | (Zhang et al., 2017) |
| 123. | alismanol G |  | / | / | / | / | / | rhizome | [α]D; UV; HRESIMS; 1D/2D NMR | Terpenoids | (Mai et al., 2015) |
| 124. | alismanol M |  | / | / | / | / | / | rhizome | HRESIMS; UV; [α]D; 1D/2D NMR (¹H; ¹³C; HSQC; HMBC;¹H -¹H COSY; NOESY) | Terpenoids | (Xin et al., 2016) |
| 125. | alismanol Q |  | / | / | / | / | / | rhizome | HRESIMS; UV; [α]D; 1D/2D NMR (¹H; ¹³C; HSQC; HMBC;¹H -¹H COSY; NOESY) | Terpenoids | (Xin et al., 2016) |
| 126. | alismanin A |  |  |  |  |  |  | rhizome | HRESIMS; 1D/2D NMR; ECD; X-ray | Terpenoids | (Chao Wang et al., 2017) |
| 127. | alismanin B |  | / | / | / | / | / | rhizome | HRESIMS; 1D/2D NMR; ;X-ray | Terpenoids | (Chao Wang et al., 2017) |
| 128. | alismanin C |  | / | / | / | / | / | rhizome | HRESIMS; 1D/2D NMR; ;X-ray | Terpenoids | (Chao Wang et al., 2017) |
| 129. | alisolide D |  | / | / | / | / | / | rhizome | HR-ESI-MS; IR; UV; [α]D; Mo₂(OAc)₄-ECD; 1D/2D NMR | Terpenoids | (Jin et al., 2019) |
| 130. | alisolide E |  | / | / | / | / | / | rhizome | HR-ESI-MS; IR; UV; [α]D; Mo₂(OAc)₄-ECD; 1D/2D NMR | Terpenoids | (Jin et al., 2019) |
| 131. | alisolide F |  | / | / | / | / | / | rhizome | HR-ESI-MS; IR; UV; [α]D; Mo₂(OAc)₄-ECD; 1D/2D NMR | Terpenoids | (Jin et al., 2019) |
| 132. | alisolide M |  | / | / | / | / | / | rhizome | HRESIMS; UV; [α]D; 1D/2D NMR | Terpenoids | (Xin et al., 2016) |
| 133. | ursolic acid |  | / | / | / | / | / | rhizome | IR; EI-MS; ¹H-NMR;  ¹³C-NMR;  DEPT;¹H-¹H COSY; HMBC; HSQC | Terpenoids | (Qiu, 2009) |
| 134. | alismoxide | a | *β-*OH | *α-* CH_3_ | *α-* OH | *α-* H | / | rhizome | NMR; MS; IR; [α]D; TLC; CC; HRMS | Sesquiterpenes | (Masayuki Yoshikawa et al., 1992; Nakajima et al., 1994; Oshima et al., 1983; Peng et al., 2003) |
| 135. | 4α, 10α-dihyroxy-5β-H-guaj-6-en | a | *β-* CH_3_ | *α-* OH | *α-* OH | *α-* H | / | rhizome | ESI-MS; ¹H-NMR;  ¹³C-NMR | Sesquiterpenes | (Zhang et al., 2009) |
| 136. | 10-O-methyl-alismoxide | a | *β-* OCH_3_ | *α-* CH_3_ | *α-* OH | *α-* H | / | rhizome | NMR; MS; IR; [α]D; TLC; CC | Sesquiterpenes | (Nakajima et al., 1994) |
| 137. | 10-O-ethyl-alismoxide | a | *β-* O CH_2_CH_3_ | *α-* CH_3_ | *α-* OH | *α-* H | / | rhizome | HR-ESI-MS;¹H-NMR;  ¹³C-NMR;HMBC | Sesquiterpenes | (Li et al., 2017) |
| 138. | 10α-hydroxy-4α-methoxy-guai-6-ene | a | *β-* CH_3_ | *α-* OH | *α-* CH_3_ | *β-*H | / | rhizome | ¹H-NMR;  ¹³C-NMR | Sesquiterpenes | (Zhao et al., 2017) |
| 139. | orientalol A | a | *β-*OH | *α-* CH_2_OH | *α-* OH | *α-* H | / | rhizome | HR-FAB-MS; IR;  ¹H-NMR;  ¹³C-NMR;NOESY | Sesquiterpenes | (Masayuki Yoshikawa et al., 1992) |
| 140. | 10-O-methyl-orientalol A | a | *β-* OCH_3_ | *α-* CH_2_OH | *α-* OH | *α-* H | / | rhizome | HR-ESI-MS;¹H-NMR;  ¹³C-NMR;IR;2D NMR | Sesquiterpenes | (Li et al., 2017) |
| 141. | orientalol B | a | *β-* CH_2_OH | *α-* OH | *α-* OH | *α-* H | / | rhizome | HR-FAB-MS; IR;  ¹H-NMR;  ¹³C-NMR;NOESY | Sesquiterpenes | (Masayuki Yoshikawa et al., 1992) |
| 142. | alismol | b | *α-* H | H | */* | */* | / | rhizome | NMR; MS; IR; [α]D; TLC; CC; NOESY; HRMS | Sesquiterpenes | (Masayuki Yoshikawa et al., 1992; Nakajima et al., 1994; Oshima et al., 1983; Peng et al., 2003) |
| 143. | 4α, 12-dihydroxyguaian-6, 10-diene | b | *α-* H | OH | / | / | / | rhizome | ¹H-NMR; ¹³C-NMR;MS | Sesquiterpenes | (Cang et al., 2017b) |
| 144. | 4β, 1-dihydroxyguaian-6, 10-dien | b | *β-*H | OH | / | / | / | rhizome | EI-MS; IR; ¹H-NMR; ¹³C-NMR; [α]D | Sesquiterpenes | (Jin et al., 2012) |
| 145. | sulfoorientalols a | c | H | *α-*SO_3_H | / | / | / | rhizome | FAB-MS;¹H-NMR | Sesquiterpenes | (Yoshikawa, Fukuda, et al., 1993) |
| 146. | sulfoorientalols b | c | SO_3_H | *α-* OH | / | / | / | rhizome | FAB-MS;¹H-NMR;IR | Sesquiterpenes | (Yoshikawa, Fukuda, et al., 1993) |
| 147. | oplopanone | d | *β-*H | / | / | / | / | rhizome | ESI-MS;¹H-NMR; ¹³C-NMR; [α]D | Sesquiterpenes | (Jingyi et al., 2016) |
| 148. | ent-oplopanone | d | *α-* H | / | / | / | / | rhizome | ¹H-NMR;¹³C-NMR;MS | Sesquiterpenes | (Ma et al., 2016) |
| 149. | 1β, 11-dihydroxy-β-cyperone | e | OH | *β-*OH | / | / | / | rhizome | ¹H-NMR; ¹³C-NMR; UV;HBMC; HRESIMS; NOESY | Sesquiterpenes | (Cang et al., 2017b) |
| 150. | 1β-hydroxy-β-cyperone | e | H | *β-*OH | / | / | / | rhizome | ¹H-NMR; ¹³C-NMR;MS | Sesquiterpenes | (Cang et al., 2017b) |
| 151. | (10S)-11-hydroxy-β-cyperone | e | OH | *β-*H | / | / | / | rhizome | HRESIMS;¹H-NMR;¹³C-NMR;UV;HBMC;[α]D | Sesquiterpenes | (Zhao et al., 2017) |
| 152. | (8R)-alismanoid A | f | *α-* H | / | / | / | / | rhizome | HRESIMS;¹H-NMR;¹³C-NMR;UV;HBMC;[α]D;ECD | Sesquiterpenes | (Yu et al., 2017) |
| 153. | (8S)-alismanoid A | f | *β-*H | / | / | / | / | rhizome | HRESIMS;¹H-NMR;¹³C-NMR;HBMC;[α]D;UV;ECD | Sesquiterpenes | (Yu et al., 2017) |
| 154. | 1αH, 5αH-guaia-6-ene-4β, 10β-diol |  | / | / | / | / | / | rhizome | ¹H-NMR;¹³C-NMR;MS | Sesquiterpenes | (Ma et al., 2016) |
| 155. | 4-epi-alismoxide |  | / | / | / | / | / | rhizome | ¹H-NMR;  ¹³C-NMR | Sesquiterpenes | (Li et al., 2017) |
| 156. | 11-hydroxy-8-ox-alismoxide |  | / | / | / | / | / | rhizome | ¹H-NMR; ¹³C-NMR; UV;HBMC; HRESIMS; NOESY | Sesquiterpenes | (Cang et al., 2017b) |
| 157. | alismorientols A |  | / | / | / | / | / | rhizome | HRESIMS;¹H-NMR;¹³C-NMR;HBMC;[α]D;UV;ECD | Sesquiterpenes | (Jiang et al., 2007; Zhang et al., 2009) |
| 158. | alismorientols B |  | / | / | / | / | / | rhizome | HRESIMS;¹H-NMR;¹³C-NMR;HBMC;[α]D;UV;ECD | Sesquiterpenes | (Jiang et al., 2007; Zhang et al., 2009) |
| 159. | 11-oxo-13-noralismol |  | / | / | / | / | / | rhizome | ¹H-NMR; ¹³C-NMR; HBMC; HRESIM | Sesquiterpenes | (Cang et al., 2017b; Li et al., 2017) |
| 160. | ligucyperonol |  | / | / | / | / | / | rhizome | ¹H-NMR; ¹³C-NMR;MS | Sesquiterpenes | (Cang et al., 2017b) |
| 161. | orientalol C |  | / | / | / | / | / | rhizome | HR-FAB-MS; IR;  ¹H-NMR;  ¹³C-NMR;NOESY | Sesquiterpenes | (Masayuki Yoshikawa et al., 1992) |
| 162. | 3β, 4β-expoxy-chrysothol |  | / | / | / | / | / | rhizome | HR-ESI-MS;¹H-NMR;  ¹³C-NMR;HMBC;NOESY | Sesquiterpenes | (Li et al., 2017) |
| 163. | orientalol E |  | / | / | / | / | / | rhizome | HR-ESI-MS; 2D NMR;IR; ¹H-NMR;  ¹³C-NMR; | Sesquiterpenes | (Peng et al., 2003) |
| 164. | orientalol F |  | / | / | / | / | / | rhizome | HR-ESI-MS; 2D NMR;IR; ¹H-NMR;  ¹³C-NMR; | Sesquiterpenes | (Peng et al., 2003) |
| 165. | orientalol G |  | / | / | / | / | / | rhizome | HR-ESI-MS;¹H-NMR;  ¹³C-NMR;HMBC;NOESY | Sesquiterpenes | (Li et al., 2017) |
| 166. | orientalol L |  | / | / | / | / | / | rhizome | HRESIMS; ¹H-NMR;  ¹³C-NMR；2D NMR | Sesquiterpenes | (Zhang et al., 2018) |
| 167. | 7α, 10α-epoxy-salvialan-10β-ol |  | / | / | / | / | / | rhizome | HRESIMS;¹H-NMR;¹³C-NMR;2D NMR | Sesquiterpenes | (Jingyi et al., 2016) |
| 168. | orientalol N |  | / | / | / | / | / | rhizome | HRESIMS; ¹H-NMR;  ¹³C-NMR；2D NMR;CD | Sesquiterpenes | (Zhang et al., 2018) |
| 169. | alismanoid B |  | / | / | / | / | / | rhizome | HRESIMS;¹H-NMR;¹³C-NMR;UV;HBMC;[α]D;ECD | Sesquiterpenes | (Yu et al., 2017) |
| 170. | alismanoid C |  | / | / | / | / | / | rhizome | HRESIMS;¹H-NMR;¹³C-NMR;UV;HBMC;[α]D;ECD | Sesquiterpenes | (Yu et al., 2017) |
| 171. | sulfoorientalols c |  | / | / | / | / | / | rhizome | FAB-MS;¹H-NMR;¹³C-NMR;IR | Sesquiterpenes | (Yoshikawa, Fukuda, et al., 1993) |
| 172. | sulfoorientalols d |  | / | / | / | / | / | rhizome | FAB-MS;¹H-NMR | Sesquiterpenes | (Yoshikawa, Fukuda, et al., 1993) |
| 173. | orientanone |  | / | / | / | / | / | whole plant e | EI-MS;ESI-MS;HR-ESI-MS;IR;¹H-NMR;  ¹³C-NMR;2D NMR (¹H-¹H COSY; TOCSY; HMQC; HMBC; NOESY);X-ray | Sesquiterpenes | (Peng et al., 2002) |
| 174. | clovandiol |  | / | / | / | / | / | rhizome | HR-TOF-MS;¹H-NMR;  ¹³C-NMR;2D NMR | Sesquiterpenes | (Zhang et al., 2009) |
| 175. | germacrene C |  | / | / | / | / | / | rhizome | ¹H-NMR; ¹³C-NMR | Sesquiterpenes | (Yoshikawa et al., 1994) |
| 176. | germacrene D |  | / | / | / | / | / | rhizome | ¹H-NMR; ¹³C-NMR | Sesquiterpenes | (Yoshikawa et al., 1994) |
| 177. | gibberodione |  | / | / | / | / | / | rhizome | ESI-MS;¹H-NMR; ¹³C-NMR; [α]D | Sesquiterpenes | (Jingyi et al., 2016) |
| 178. | eudesma-4(14)-ene-1β, 6a-diol |  | / | / | / | / | / | rhizome | NMR; MS; IR; [α]D; TLC; CC | Sesquiterpenes | (Nakajima et al., 1994) |
| 179. | orientalol O |  | / | / | / | / | / | rhizome | HRESIMS;¹H-NMR;  ¹³C-NMR；2D NMR;CD | Sesquiterpenes | (Zhang et al., 2018) |
| 180. | orientalol P |  | / | / | / | / | / | rhizome | HRESIMS;¹H-NMR;  ¹³C-NMR；2D NMR;CD | Sesquiterpenes | (Zhang et al., 2018) |
| 181. | zingibertriol |  | / | / | / | / | / | rhizome | ¹H-NMR;¹³C-NMR | Sesquiterpenes | (Zhao et al., 2017) |
| 182. | alisguaiaone |  | / | / | / | / | / | rhizome | ¹H-NMR;¹³C-NMR;MS | Sesquiterpenes | (Ma et al., 2016) |
| 183. | litseachromolaevane B |  | / | / | / | / | / | rhizome | ¹H-NMR;¹³C-NMR | Sesquiterpenes | (Zhao et al., 2017) |
| 184. | orientalol M |  | / | / | / | / | / | rhizome | HRESIMS; ¹H-NMR;  ¹³C-NMR；2D NMR;CD;ECD | Sesquiterpenes | (Zhang et al., 2018) |
| 185. | oriediterpenone | g | H | \| / \| / \| / \| / \| \| --- \| --- \| --- \| --- \| | / | / | / | rhizome | ¹H -NMR;  ¹³C-NMR; DEPT; ¹H-¹H COSY; HSQC; HMB | Diterpene | (Peng & Lou, 2002) |
| 186. | oriediterpenol | g | *α-* OH | / | / | / | / | rhizome | ¹H -NMR;  ¹³C-NMR; DEPT; ¹H-¹H COSY; HSQC; HMB | Diterpene | (Peng & Lou, 2002) |
| 187. | oriediterpenoside |  | / | / | / | / | / | rhizome | ¹H -NMR;  ¹³C-NMR; DEPT; ¹H-¹H COSY; HSQC; HMB | Diterpene | (Peng & Lou, 2002) |
| 188. | 12-deoxyphorbol-13a-pentadecanoate |  | / | / | / | / | / | Rhizome | NMR;MS | Diterpene | (Wang et al., 2017) |
