# Supplementary material for: Botany, traditional uses, phytochemistry, pharmacology, toxicology and processing of Rhizoma alismatis: a review
Source: Front Pharmacol. 2025 Dec 4;16:1722483. doi: 10.3389/fphar.2025.1722483 (PMC12712712; doi:10.3389/fphar.2025.1722483)
Supplement: Supplementary file 5 [file Table3.docx]

Table S3. Other compounds in *Rhizoma alismatis*.

| **Number** | **Name** | **Categories** | **Part of plant** | **Identification methods** | **Refs.** |
| --- | --- | --- | --- | --- | --- |
| 189 | uracil | nitrogen-containing compounds | rhizome | TLC;¹H-NMR; ¹³C- NMR | (Lu, 2014) |
| 190 | adenine | nitrogen-containing compounds | rhizome | TLC;¹H-NMR; ¹³C-NMR | (Lu, 2014) |
| 191 | uridine | nitrogen-containing compounds | rhizome | IR; EI-MS; H-NMR; ¹³C-NMR | (Xian et al., 1999) |
| 192 | thymine | nitrogen-containing compounds | rhizome | ¹H-NMR; ¹³C-NMR | (Zhang, 2015) |
| 193 | N-(3'-maleimidyl)-5-hydroxymethyl-2-pyrrole formaldehyde | nitrogen-containing compounds | rhizome | ¹H-NMR; ¹³C-NMR | (Zhang, 2015) |
| 194 | niacinamide | nitrogen-containing compounds | rhizome | EI-MS; ¹H-NMR; ¹³C-NMR; 2D NMR; IR; UV | (Hong et al., 2008) |
| 195 | 4-pyrazin-2-yl-but-3-ene-1，2-diol | nitrogen-containing compounds | rhizome | CI-MS; ¹H-NMR; ¹³C-NMR; 2D NMR; IR; UV | (Hong et al., 2008) |
| 196 | 1H-indole-3-carboxylic acid | nitrogen-containing compounds | rhizome | ESI-MS; ¹H-NMR; ¹³C-NMR; TLC | (C. Zhang et al., 2009) |
| 197 | 1H-indole-3-carboxadehyde | nitrogen-containing compounds | rhizome | NMR (1D; 2D); HRESIMS | (Zhao et al., 2017) |
| 198 | indazole | nitrogen-containing compounds | rhizome | NMR (1D; 2D); HRESIMS | (Zhao et al., 2017) |
| 199 | neoechinulin A | phenylpropanoids | rhizome | NMR (1D; 2D); HRESIMS | (Zhao et al., 2017) |
| 200 | magnolamide | phenylpropanoids | rhizome | NMR (1D; 2D); HRESIMS | (Zhao et al., 2017) |
| 201 | syringaresinol | phenylpropanoids | rhizome | NMR (1D; 2D); HRESIMS | (Zhao et al., 2017) |
| 202 | pinoresinol | phenylpropanoids | rhizome | NMR (1D; 2D); HRESIMS | (Qiu, 2009) |
| 203 | pinoresinol-4-O-β-D-glucoside | phenylpropanoids | rhizome | NMR (1D; 2D); HRESIMS | (Zhao et al., 2017) |
| 204 | isoeucommin A | phenylpropanoids | rhizome | NMR (1D; 2D); HRESIMS | (Zhao et al., 2017) |
| 205 | (7,8-cis-8,8-trans)-2,4-dihydroxy-3,5-dimethoxy-lariciresinol | phenylpropanoids | rhizome | NMR (1D; 2D); HRESIMS | (Zhao et al., 2017) |
| 206 | 1-(4-hydroxy-3-methoxy phenyl)-propane-1,2,3-triol | phenylpropanoids | rhizome | NMR (1D; 2D); HRESIMS | (Zhao et al., 2017) |
| 207 | 7-hydroxycoumarin | phenylpropanoids | rhizome | UV; ¹H NMR | (Qiu, 2009) |
| 208 | N-trans-feruloyl-N'-cis-feruloyl-cadaverine | phenylpropanoids | rhizome | HRESIMS; ¹H-NMR; ¹³C-NMR ;COSY; HMBC; NOESY | (Yu et al., 2017) |
| 209 | N-trans-feruloyl-N'-cis-feruloyl-3-hydroxy-cadaverine | phenylpropanoids | rhizome | HRESIMS; ¹H-NMR; ¹³C-NMR ; HMBC | (Yu et al., 2017) |
| 210 | N，N'-trans-diferuloyl-3-oxo-cadaverine | phenylpropanoids | rhizome | HRESIMS; ¹H-NMR; ¹³C-NMR ; HMBC | (Yu et al., 2017) |
| 211 | N-trans-p-coumaroyl-N'-trans-feruloyl-3-hydroxy-cadaverine | phenylpropanoids | rhizome | HRESIMS; ¹H-NMR; ¹³C-NMR ; HMBC | (Yu et al., 2017) |
| 212 | diferuloyl-cadaverine | phenylpropanoids | rhizome | ¹H-NMR; ¹³C-NMR | (Yu et al., 2017) |
| 213 | N，N'-cis-diferuloyl-3-hydroxy-cadaverine | phenylpropanoids | rhizome | HRESIMS; ¹H-NMR; ¹³C-NMR ; HMBC | (Yu et al., 2017) |
| 214 | N，N'-diferuloyl-putrescine | phenylpropanoids | rhizome | ¹H-NMR; ¹³C-NMR | (Yu et al., 2017) |
| 215 | (E，Z)-terrestribisamide | phenylpropanoids | rhizome | ¹H-NMR; ¹³C-NMR | (Yu et al., 2017) |
| 216 | (Z，Z)-terrestribisamide | phenylpropanoids | rhizome | ¹H-NMR; ¹³C-NMR | (Yu et al., 2017) |
| 217 | β-sitosterol | steroid | rhizome | TLC;IR | (Peng et al., 1999) |
| 218 | β-sitosterol-3-O-6-stearoylglucoside | steroid | rhizome | IR; ¹H NMR; ¹³C NMR | (Wu et al., 1988) |
| 219 | β-sitosterol-3-O-stearic acid ester | steroid | rhizome | m.p.;IR; ¹H-NMR; ¹³C-NMR;  TLC | (Cai et al., 1996) |
| 220 | ergosta-6,22-diene-3β, 5α, 8α-triol | steroid | rhizome | ¹H-NMR; ¹³C-NMR;DEPT | (Qiu, 2009) |
| 221 | daucosterol-6 ' -O-stearic acid ester | steroid | rhizome | TLC;IR | (Peng et al., 1999) |
| 222 | claucosterol | steroid | rhizome | IR; FAB-MS;¹H-NMR; ¹³C-NMR | (Peng et al., 1999) |
| 223 | amentoflavone | flavonoids | rhizome | NMR;MS | (Hu et al., 2008) |
| 224 | robustaflavone | flavonoids | rhizome | NMR;MS | (Hu et al., 2008) |
| 225 | 2,2 ', 4-trihydroxy chalcone | flavonoids | rhizome | NMR;MS | (Hu et al., 2008) |
| 226 | 7,4 ' -dihydroxyisoflavone | flavonoids | rhizome | ¹H-NMR; ¹³C-NMR | (Qiu, 2009) |
| 227 | calycosin | flavonoids | rhizome | ¹H-NMR; ¹³C-NMR;UV | (Qiu, 2009) |
| 228 | luteolin | flavonoids | rhizome | ¹H-NMR; ¹³C-NMR;UV | (Qiu, 2009) |
| 229 | apigenin | flavonoids | rhizome | ¹H-NMR; ¹³C-NMR;UV | (Qiu, 2009) |
| 230 | plantain A | phenolic acids | whole plante | HR-ESIMS; ¹H NMR; ¹³C NMR; ¹H-¹H COSY; HMBC; ROESY; IR;HPLC | (Huang et al., 2017) |
| 231 | ferulic acid | phenolic acids | whole plante | ¹H NMR; ¹³C NMR | (Huang et al., 2017) |
| 232 | rynchopeterine A | phenolic acids | whole plante | ¹H NMR; ¹³C NMR | (Huang et al., 2017) |
| 233 | rynchopeterine B | phenolic acids | whole plante | ¹H NMR; ¹³C NMR | (Huang et al., 2017) |
| 234 | rosmarinic acid | phenolic acids | whole plante | ¹H NMR; ¹³C NMR | (Huang et al., 2017) |
| 235 | p-hydroxybenzaldehyde | phenolic acids | rhizome | ¹H-NMR | (Qiu, 2009) |
| 236 | (Z)-8，11,12-trihydroxyoctadec-9-enoic acid | Aliphatic hydrocarbons and derivatives | rhizome | ¹H-NMR; ¹³C-NMR | (Zhang, 2015) |
| 237 | 2'，3'-dihydroxypropylentade | Aliphatic hydrocarbons and derivatives | rhizome | ¹H-NMR; ¹³C-NMR | (Zhang, 2015) |
| 238 | succinic acid | Aliphatic hydrocarbons and derivatives | rhizome | TLC;¹H-NMR; ¹³C-NMR | (Lu, 2014) |
| 239 | palmitin | Aliphatic hydrocarbons and derivatives | rhizome | EI-MS; ¹H-NMR; ¹³C-NMR | (Hong et al., 2008) |
| 240 | 1-monolinolein | Aliphatic hydrocarbons and derivatives | rhizome | EI-MS; ¹H-NMR; ¹³C-NMR | (Hong et al., 2008) |
| 241 | n-tricosane | Aliphatic hydrocarbons and derivatives | rhizome | EI-MS | (Cai et al., 1996) |
| 242 | ethyl n-octadecanoate | Aliphatic hydrocarbons and derivatives | rhizome | ¹H-NMR; ¹³C-NMR;EI-MS | (Qiu, 2009) |
| 243 | methylpalmitate | Aliphatic hydrocarbons and derivatives | rhizome | ¹H-NMR;EI-MS | (Qiu, 2009) |
| 244 | 1-dicosanol | Aliphatic hydrocarbons and derivatives | rhizome | IR; EI-MS; H-NMR; ¹³C-NMR | (Xian et al., 1999) |
| 245 | dulcitol | Aliphatic hydrocarbons and derivatives | rhizome | IR; EI-MS; H-NMR; ¹³C-NMR | (Xian et al., 1999) |
| 246 | stearic acid | Aliphatic hydrocarbons and derivatives | rhizome | m.p.;IR;EIMS;  TLC | (Cai et al., 1996) |
| 247 | 1-glycerol stearate | Aliphatic hydrocarbons and derivatives | rhizome | m.p.;IR; ¹H-NMR; ¹³C-NMR; EIMS | (Cai et al., 1996) |
| 248 | emodin | Aliphatic hydrocarbons and derivatives | rhizome | m.p.;IR; ¹H-NMR; ¹³C-NMR; EIMS;UV | (Cai et al., 1996) |
| 249 | alisman SI | Carbohydrate | Tuber | GC-MS; ¹³C NMR; TLC;HPLC | (Shimizu et al., 1994) |
| 250 | alisman PⅡ | Carbohydrate | Tuber | TLC; GC-MS;¹³C NMR | (Tomoda et al., 1994) |
| 251 | alisman P ⅢF | Carbohydrate | Tuber | GC-MS; ¹³C NMR; TLC;HPLC | (Shimizu et al., 1994; Tomoda et al., 1994) |
| 252 | manninotriose | Carbohydrate | rhizome | NMR | (Zhang et al., 2009) |
| 253 | verbascotetraose | Carbohydrate | rhizome | NMR | (Zhang et al., 2009) |
| 254 | verbascose | Carbohydrate | rhizome | NMR | (Zhang et al., 2009) |
| 255 | raffinose | Carbohydrate | rhizome | NMR | (Zhang et al., 2009) |
| 256 | stachyose | Carbohydrate | rhizome | NMR | (Zhang et al., 2009) |
| 257 | sucrose | Carbohydrate | rhizome | NMR | (Zhang et al., 2009) |
| 258 | β-D-fructofuranose | Carbohydrate | rhizome | NMR | (Zhang et al., 2009) |
| 259 | 5-hydroxymethylfuraldehyde | Carbohydrate | rhizome | NMR | (Zhang et al., 2009) |
| 260 | α-D-fructofuranose | Carbohydrate | rhizome | NMR | (Zhang et al., 2009) |
| 261 | ethylα-D-fructofuranoside | Carbohydrate | rhizome | NMR | (Zhang et al., 2009) |
| 262 | ethylβ-D-fructofuranoside | Carbohydrate | rhizome | NMR | (Zhang et al., 2009) |
