# Supplementary material for: Botany, traditional uses, phytochemistry, pharmacology, toxicology and processing of Rhizoma alismatis: a review
Source: Front Pharmacol. 2025 Dec 4;16:1722483. doi: 10.3389/fphar.2025.1722483 (PMC12712712; doi:10.3389/fphar.2025.1722483)
Supplement: Supplementary file 6 [file DataSheet1.docx]

# Supplementary Material Reference:

Cai, L., Wang, H., Cao, H., & Zhang, R. (1996). Studies on chemical constituents of Rhizoma alismatis. *Natural Product Research and Development*(01), 5-9. https://doi.org/10.16333/j.1001-6880.1996.01.002

Cang, J., Wang, C., Huo, X.-K., Tian, X.-G., Sun, C.-P., Deng, S.,…Ma, X.-C. (2017a). Sesquiterpenes and triterpenoids from the rhizomes of Alisma orientalis and their pancreatic lipase inhibitory activities. *Phytochemistry Letters*, *19*, 83-88. https://doi.org/10.1016/j.phytol.2016.12.017

Cang, J., Wang, C., Huo, X., Tian, X., Sun, C., Deng, S.,…Ma, X. (2017b). Sesquiterpenes and triterpenoids from the rhizomes of Alisma orientalis and their pancreatic lipase inhibitory activities. *Phytochemistry Letters*, *19*, 83-88. https://doi.org/10.1016/j.phytol.2016.12.017

Fukuyama, Y., Pei-Wu, G., Rei, W., Yamada, T., & Nakagawa, K. (2007). 11-Deoxyalisol C and Alisol D: New Protostane-Type Triterpenoids fromAlisma plantago-aquatica. *Planta Medica*, *54*(05), 445-447. https://doi.org/10.1055/s-2006-962495

Han, C. W., Kwun, M. J., Kim, K. H., Choi, J.-Y., Oh, S.-R., Ahn, K.-S.,…Joo, M. (2013). Ethanol extract of Alismatis Rhizoma reduces acute lung inflammation by suppressing NF-κB and activating Nrf2. *Journal of Ethnopharmacology*, *146*(1), 402-410. https://doi.org/10.1016/j.jep.2013.01.010

Hong, C., Pu, X., & Lou, C. (2008). Isolation and ldentification of Chemical Constituents from Alisma orientale. *Journal of Chongqing University of Technology(Natural Science)*(04), 78-81.

Hu, X., Guo, Y., Gao, W., Chen, H., & Zhang, T. (2008). A new triterpenoid from Alisma orientalis. *Chinese Chemical Letters*, *19*(4), 438-440. https://doi.org/10.1016/j.cclet.2008.01.019

Hu, X., Guo, Y., Gao, W., Zhang, T., & Chen, H. (2008a). A new triterpenoid from Alisma orientalis. *Chinese Chemical Letters*, *19*(4), 438-440. https://doi.org/10.1016/j.cclet.2008.01.019

Hu, X., Guo, Y., Gao, W., Zhang, T., & Chen, H. (2008b). Two new triterpenes from the rhizomes ofAlisma orientalis. *Journal of Asian Natural Products Research*, *10*(5), 481-484. https://doi.org/10.1080/10286020801948441

Huang, Y., Yu, Q., Chen, Y., Cheng, M., & Xie, L. (2017). Phenolic constituents from Alisma plantago-aquatica Linnaeus and their anti-chronic prostatitis activity. *Chemistry Central Journal*, *11*(1). https://doi.org/10.1186/s13065-017-0350-9

Jiang, Z., Zhang, X., Zhang, F., Liu, N., Zhao, F., Zhou, J., & Chen, J.-J. (2006). A New Triterpene and Anti-Hepatitis B Virus Active Compounds from Alisma orientalis. *Planta Medica*, *72*(10), 951-954. https://doi.org/10.1055/s-2006-947178

Jiang, Z., Zhang, X., Zhou, J., Zhang, F., Chen, J., Lü, Y.,…Zheng, Q. (2007). Two New Sesquiterpenes from Alisma orientalis. *Chemical and Pharmaceutical Bulletin*, *55*(6), 905-907. https://doi.org/10.1248/cpb.55.905

Jin, H.-G., Jin, Q., Ryun Kim, A., Choi, H., Lee, J. H., Kim, Y. S.,…Woo, E.-R. (2012). A new triterpenoid from Alisma orientale and their antibacterial effect. *Archives of Pharmacal Research*, *35*(11), 1919-1926. https://doi.org/10.1007/s12272-012-1108-5

Jin, Q., Zhang, J., Hou, J., Lei, M., Liu, C., Wang, X.,…Guo, D. (2019). Novel C-17 spirost protostane-type triterpenoids from Alisma plantago-aquatica with anti-inflammatory activity in Caco-2 cells. *Acta Pharmaceutica Sinica B*, *9*(4), 809-818. https://doi.org/10.1016/j.apsb.2019.04.002

Jingyi, Y., Jun, W., Hong, L., Qingying, Z., Shizhong, C., & Pengfei, T. (2016). Sesquiterpenes from Alisma plantago-aquatica. *Journal of Chinese Pharmaceutical Sciences*, *25*(8), 614-620. https://doi.org/10.5246/jcps.2016.08.069

Li, H., Chen, X., Luo, D., Fan, M., Zhang, Z., Peng, L.,…Zhao, Q. (2017). Protostane‐Type Triterpenoids from Alisma orientale. *Chemistry & Biodiversity*, *14*(12). https://doi.org/10.1002/cbdv.201700452

Li, H., Fan, M., Xue, Y., Peng, L., Wu, X., Liu, D.,…Zhao, Q. (2017). Guaiane-Type Sesquiterpenoids from Alismatis Rhizoma and Their Anti-inflammatory Activity. *Chemical & Pharmaceutical Bulletin*, *65*(4), 403-407. https://doi.org/10.1248/cpb.c16-00798

Li, H., Liu, D., Dai, W., Chen, X., & Li, R. (2018). A new protostane-type triterpenoid from Alisma plantago-aquatica subsp. orientale (Sam.) Sam. *Natural Product Research*, *33*(21), 3083-3088. https://doi.org/10.1080/14786419.2018.1519710

Li, Q. (2013). *Studies on Active Components in Nauclea officinalis and Alisma orientale* [D, Zhejiang University]. https://next.cnki.net/middle/abstract?v=pTDtInUJxYxAk4xgLV-KZba9bn-IBd5vsmCEG7AFSNrD1wUq2H1XMWRBijpoMFSjvLmSFgTFlueGecqnfxcV3Ll07PZGw6YPYuyXn3uzAXa-h9olpMlg8D6FIKrRyIzkM86a9hl1Y5PVrj46rZMYsmBnh9o_fVwG4WBRkEOhl8Tu_Y9IR6R2OPrrKmZiZQ081dXRfg5oROU=&uniplatform=NZKPT&language=CHS&scence=null

Li, S., Jin, S., Song, C., Jia, S., Zhang, Y., Feng, Y.,…Jiang, H. (2017). The strategy for establishment of the multiple reaction monitoring based characteristic chemical profile of triterpenes in Alismatis rhizoma using two combined tandem mass spectrometers. *Journal of Chromatography A*, *1524*, 121-134. https://doi.org/10.1016/j.chroma.2017.09.057

Liu, S., Sheng, W., Li, Y., Zhang, S., Zhu, J., Gao, H.,…Zhang, M. (2019). Chemical constituents from Alismatis Rhizoma and their anti-inflammatory activities in vitro and in vivo. *Bioorganic Chemistry*, *92*. https://doi.org/10.1016/j.bioorg.2019.103226

Lu, Y. (2014). *Study on the hyperglycemic components of Alisma Orentalis by Chromatography* [硕士, Nanchang University]. https://next.cnki.net/middle/abstract?v=D99zBTMMfgxCehZOEXGbdvdjm9oI0sHuRf4Mna9QyUxwf4HFfPT9MMk5Z3jYU3OKiXaWwT1RevpKg0eNbEt9yWHmVrZ-wlrzfRdrk-qDzPaVXhVW0o965HlKj3qqxn8cSdYEjA-ZJ7Y6InQWVv6-Q8YAHDsraAF6fNE9C22pWaeyVRB0oMqkMGyomvJc2jtKS9IE741Z1r8=&uniplatform=NZKPT&language=CHS&scence=null

Ma, Q., Han, L., Bi, X., Wang, X., Mu, Y., Guan, P.,…Huang, X. (2016). Structures and biological activities of the triterpenoids and sesquiterpenoids from Alisma orientale. *Phytochemistry*, *131*, 150-157. https://doi.org/10.1016/j.phytochem.2016.08.015

Mai, Z., Zhou, K., Ge, G., Wang, C., Huo, X., Dong, P.,…Ma, X. (2015). Protostane Triterpenoids from the Rhizome of Alisma orientale Exhibit Inhibitory Effects on Human Carboxylesterase 2. *Journal of Natural Products*, *78*(10), 2372-2380. https://doi.org/10.1021/acs.jnatprod.5b00321

Masayuki Yoshikawa, Shoko Hatakeyama, Nobumitsu Tanaka, Youichi Fukuda, Nobutoshi Murakami, & Yamahara, J. (1992). Orientalols A, B, and C, sesquiterpene constituents from Chinese Alismatis Rhizoma, and revised structures of alismol and alismoxide. *Chemical and Pharmaceutical Bulletin*, *40*(9), 2582-2584. https://doi.org/10.1248/cpb.40.2582

Matsuda, H., Kageura, T., Toguchida, I., Murakami, T., Kishi, A., & Yoshikawa, M. (1999). Effects of sesquiterpenes and triterpenes from the rhizome of Alisma orientale on nitric oxide production in lipopolysaccharide-activated macrophages: Absolute stereostructures of alismaketones-B 23-acetate and -C 23-acetate. *Bioorganic & Medicinal Chemistry Letters*, *9*(21), 3081-3086. https://doi.org/10.1016/s0960-894x(99)00536-3

Murata, T., Imai, Y., Hirata, T., & Miyamoto, M. (1970). Biological-Active Triterpenes of Alismatis Rhizoma. I. Isolation of the Alisols. *Chemical and Pharmaceutical Bulletin*, *18*(7), 1347-1353. https://doi.org/10.1248/cpb.18.1347

Nakajima, Y., Satoh, Y., Ohtsuka, N., Tsujiyama, K., Mikoshiba, N., Ida, Y., & Shoji, J. (1994). Terpenoids of Alisma-Orientale Rhizome and the Crude Drug Alismatis Rhizoma. *Phytochemistry*, *36*(1), 119-127. https://doi.org/Doi 10.1016/S0031-9422(00)97024-9

Oshima, Y., Iwakawa, T., & Hikino, H. (1983). Alismol and alismoxide, sesquiterpenoids of Alisma rhizomes. *Phytochemistry*, *22*(1), 183-185. https://doi.org/10.1016/s0031-9422(00)80084-9

Pei-Wu, G., Fukuyama, Y., Rei, W., Jinxian, B., & Nakagawa, K. (1988). An acylated sitosterol glucoside from Alisma plantago-aquatica. *Phytochemistry*, *27*(6), 1895-1896. https://doi.org/10.1016/0031-9422(88)80475-8

Pei-Wu, G., Fukuyama, Y., Yamada, T., Rei, W., Jinxian, B., & Nakagawa, K. (1988). Triterpenoids from the rhizome of Alisma plantago-aquatica. *Phytochemistry*, *27*(4), 1161-1164. https://doi.org/10.1016/0031-9422(88)80294-2

Peng, G.-P., Lou, F.-C., Huang, X.-F., & Tian, G. (2002). Structure of orientanone from Alisma orientalis, a novel sesquiterpene originating from guaiane-type carbon skeleton by isopropyl shift. *Tetrahedron*, *58*(44), 9045-9048. https://doi.org/10.1016/s0040-4020(02)01128-6

Peng, G., & Lou, F. (2001). Terpenoids of Alisma Orientalis Juzep. *Natural Product Research and Development*(04), 1-4. https://doi.org/10.16333/j.1001-6880.2001.04.001

Peng, G., & Lou, F. (2002). Isolation and Identification of Diterpenes from Alisma Orientalis Juzep. *Acta Pharmaceutica Sinica*, *37*(12), 950-954.

Peng, G., Tian, G., Huang, X., & Lou, F. (2003). Guaiane-type sesquiterpenoids from Alisma orientalis. *Phytochemistry*, *63*(8), 877-881. https://doi.org/10.1016/s0031-9422(03)00222-x

Peng, G., Zhu, G., & Lou, F. (2002a). Terpenoids from Alisma Orientalis Juzep. *Natural Product Research and Development*(06), 7-10. https://doi.org/10.16333/j.1001-6880.2002.06.003

Peng, G., Zhu, G., & Lou, F. (2002b). Two Novel Terpenoids from Alisma orientalis Juzep. *Natural Product Research and Development*(04), 5-8. https://doi.org/10.16333/j.1001-6880.2002.04.002

Peng, X., Tan, L., Yao, B., & Zhang, R. (1999). Studies on the Constituents from the Rhizoma of Alisma orientalis. *Journal of Chinese Pharmaceutical Sciences*(03), 173-174.

Qiu, D. (2009). *Studies on the Chemical Constituentsof Alisma orientalis (sam.). Juzep* [master, Lanzhou University]. https://next.cnki.net/middle/abstract?v=4mdsUcMtJE2dtoHcINexXVXQagQEAEFbpeLZCKcIKDeFF4vTPX9oWlMbHaGwveGynHSAQnwhEpiRissvcrpfLO0Ilh49Rf9hZLtIOoLbhZlEVxvz2qH80bLix4aSkxQocrUs7p6YPmml7kO_zSbDoT1Gd8_OuFFsK-CfWRTU57KmWSGpAdvYvXNZtD1n2nTXPmwyJJ_u8bc=&uniplatform=NZKPT&language=CHS&scence=null

Shimizu, N., Ohtsu, S., Tomoda, M., Gonda, R., & Ohara, N. (1994). A Glucan with Immunological Activities from the Tuber of Alisma orientale. *Biological and Pharmaceutical Bulletin*, *17*(12), 1666-1668. https://doi.org/10.1248/bpb.17.1666

Song, C., Huang, L., Huang, X., Huang, R., Peng, M., Le, Z.,…Fang, N. (2013). Characterization of Protostane Triterpenoids in Dried Tuber of Alisma orientalis by Q-TOF Mass Spectrometry in Both Positive and Negative Modes. *Asian Journal of Chemistry*, *25*(18), 10296-10304. https://doi.org/10.14233/ajchem.2013.15286

Tian, T., Chen, H., & Zhao, Y. Y. (2014). Traditional uses, phytochemistry, pharmacology, toxicology and quality control of (Sam.) Juzep: A review. *Journal of Ethnopharmacology*, *158*, 373-387. https://doi.org/10.1016/j.jep.2014.10.061

Tomoda, M., Gonda, R., Shimizu, N., & Ohara, N. (1994). Characterization of an Acidic Polysaccharide Having Immunological Activities from the Tuber of Alisma orientale. *Biological and Pharmaceutical Bulletin*, *17*(5), 572-576. https://doi.org/10.1248/bpb.17.572

Wang, C., Huo, X., Luan, Z., Cao, F., Tian, X., Zhao, X.,…Ma, X. (2017). Alismanin A, a Triterpenoid with a C34 Skeleton from Alisma orientale as a Natural Agonist of Human Pregnane X Receptor. *Organic Letters*, *19*(20), 5645-5648. https://doi.org/10.1021/acs.orglett.7b02738

Wang, Y., Zhao, J., Liang, J., Tian, X., Huo, X., Feng, L.,…Sun, C. (2017). A bioactive new protostane-type triterpenoid from Alisma plantago-aquatica subsp. orientale (Sam.) Sam. *Natural Product Research*, *33*(6), 776-781. https://doi.org/10.1080/14786419.2017.1408106

Xian, P., Li, T., Bing, Y., & Ruyi, Z. (1999). Studies on the Constituents from the Rhizoma of Alisma orientalis. *8*(3), 173-174. https://doi.org/%J Journal of Chinese Pharmaceutical Sciences

Xin, X., Mai, Z., Wang, X., Chen, L., Deng, S., & Zhang, B. (2016). Protostane alisol derivatives from the rhizome of Alisma orientale. *Phytochemistry Letters*, *16*, 8-11. https://doi.org/10.1016/j.phytol.2016.02.008

Xin, X., Yu, Z., Tian, X., Wei, J., Wang, C., Huo, X.,…Fan, G. (2017). Phenylpropanoid amides from Alisma orientalis and their protective effects against H2O2 -induced damage in SH-SY5Y cells. *Phytochemistry Letters*, *21*, 46-50. https://doi.org/10.1016/j.phytol.2017.05.027

Xin, X., Zhao, X., Huo, X., Tian, X., Sun, C., Zhang, H.,…Wang, X. (2017). Two new protostane-type triterpenoids from Alisma orientalis. *Natural Product Research*, *32*(2), 189-194. https://doi.org/10.1080/14786419.2017.1344660

Xu, Z., Zhang, H., & Xie, X. (2012). A new triterpene in rhizome of Alisma orientale. *Chinese Traditional and Herbal Drugs*, *43*(05), 841-843.

Yi, J., Bai, R., An, Y., Liu, T., Liang, J., Tian, X.,…Zhang, H. (2019). A natural inhibitor from Alisma orientale against human carboxylesterase 2: Kinetics, circular dichroism spectroscopic analysis, and docking simulation. *International Journal of Biological Macromolecules*, *133*, 184-189. https://doi.org/10.1016/j.ijbiomac.2019.04.099

Yoshikawa, M., Fukuda, Y., Hatakeyama, S., Tanaka, N., Matsuda, H., Yamahara, J., & Murakami, N. (1993). Sulfoorientalols a, b, c, and d, four new biologically active sesquiterpenes, from alismatis rhizoma. *Chemical and Pharmaceutical Bulletin*, *41*(6), 1194-1196. https://doi.org/10.1248/cpb.41.1194

Yoshikawa, M., Hatakeyama, S., Tanaka, N., Fukuda, Y., Yamahara, J., & Murakami, N. (1993). Crude Drugs from Aquatic Plants. I. On the Constituents of Alismatis Rhizoma. (1). Absolute Stereostructures of Alisols E 23-Acetate, F, and G, Three New Protostane-Type Triterpenes from Chinese Alismatis Rhizoma. *Chemical and Pharmaceutical Bulletin*, *41*(11), 1948-1954. https://doi.org/10.1248/cpb.41.1948

Yoshikawa, M., Murakami, T., Ikebata, A., Ishikado, A., Murakami, N., Yamahara, J., & Matsuda, H. (1997). Absolute Stereostructures of Alismalactone 23-Acetate and Alismaketone-A 23-Acetate, New seco-Protostane and Protostane-Type Triterpenes with Vasorelaxant Effects from Chinese Alismatis Rhizoma. *Chemical and Pharmaceutical Bulletin*, *45*(4), 756-758. https://doi.org/10.1248/cpb.45.756

Yoshikawa, M., Tomohiro, N., Murakami, T., Ikebata, A., Matsuda, H., Matsuda, H., & Kubo, M. (1999). Studies on Alismatis Rhizoma. III. Stereostructures of New Protostane-Type Triterpenes, Alisols H, I, J-23-Acetate, K-23-Acetate, L-23-Acetate, M-23-Acetate, and N-23-Acetate, from the Dried Rhizome of Alisma orientale. *Chemical and Pharmaceutical Bulletin*, *47*(4), 524-528. https://doi.org/10.1248/cpb.47.524

Yoshikawa, M., Yamaguchi, S., Matsuda, H., Tanaka, N., Yamahara, J., & Murakami, N. (1994). Crude Drugs from Aquatic Plants. V. On the Constituents of Alismatis Rhizoma. (3). Stereostructures of Water-Soluble Bioactive Sesquiterpenes, Sulfoorientalols a,b,c, and d, from Chinese Alismatis Rhizoma. *Chemical and Pharmaceutical Bulletin*, *42*(12), 2430-2435. https://doi.org/10.1248/cpb.42.2430

Yu, Z., Peng, Y., Wang, C., Cao, F., Huo, X., Tian, X.,…Ma, X. (2017). Alismanoid A, an unprecedented 1,2-seco bisabolene from Alisma orientale, and its protective activity against H2O2-induced damage in SH-SY5Y cells. *New J. Chem.*, *41*(21), 12664-12670. https://doi.org/10.1039/c7nj01806a

Zhang, C., Zhou, A., & Zhang, m. (2009). Chemical constituents of Alisma orientalis and their immunosuppressive function. *China Journal of Chinese Materia Medica*, *34*(08), 994-998. https://doi.org/10.3321/j.issn:1001-5302.2009.08.016

Zhang, J., Jin, Q., Li, S., Wu, J., Wang, Z., Hou, J.,…Guo, D. (2018). Orientalol L–P, novel sesquiterpenes from the rhizome of Alisma orientale (Sam.) Juzep and their nephrotoxicity on HK2 cells. *New Journal of Chemistry*, *42*(16), 13414-13420. https://doi.org/10.1039/c8nj02027b

Zhang, Y. (2015). *Chemical and Bioactive Studies on Two Species of Traditonal Chinese Medicine* [硕士, Shandong University]. https://next.cnki.net/middle/abstract?v=D99zBTMMfgzOFkmIsnAY9v1FQklcn5zJhyhRrwdtyFSsg33krsuRUw7O1X5wtkhttCCxrjEw1m7H4j0SNPTJIQZ8IJnP05EhJd9K251Vp1cryZ4_gQ8Ow8c5ltP0ktfidDKSKb--nY4EH9m77q-DnWE5nLY8KJzLd6hWqH1mO0CjlSlYftlv5navDfRqBQC7DL17daXhjEs=&uniplatform=NZKPT&language=CHS&scence=null

Zhang, Z., Huo, X., Tian, X., Feng, L., Ning, J., Zhao, X.,…Liu, Y. (2017). Novel protostane-type triterpenoids with inhibitory human carboxylesterase 2 activities. *RSC Advances*, *7*(46), 28702-28710. https://doi.org/10.1039/c7ra04841f

Zhang, Z., Wang, D., Zhao, Y., Gao, H., Hu, Y.-H., & Hu, J.-F. (2009). Fructose-derived carbohydrates fromAlisma orientalis. *Natural Product Research*, *23*(11), 1013-1020. https://doi.org/10.1080/14786410802391120

Zhao, M., Xu, L., & Che, C. (2008). Alisolide, alisols O and P from the rhizome of Alisma orientale. *Phytochemistry*, *69*(2), 527-532. https://doi.org/10.1016/j.phytochem.2007.06.014

Zhao, W., Huang, X., Li, X., Zhang, F., Chen, S., Ye, M.,…Wu, S. (2015). Qualitative and Quantitative Analysis of Major Triterpenoids in Alismatis Rhizoma by High Performance Liquid Chromatography/Diode-Array Detector/Quadrupole-Time-of-Flight Mass Spectrometry and Ultra-Performance Liquid Chromatography/Triple Quadrupole Mass Spectrometry. *Molecules*, *20*(8), 13958-13981. https://doi.org/10.3390/molecules200813958

Zhao, X., Wang, G., Wang, Y., Tian, X., Zhao, J., Huo, X.,…Wang, X. (2017). Chemical constituents from Alisma plantago-aquatica subsp. orientale (Sam.) Sam and their anti-inflammatory and antioxidant activities. *Natural Product Research*, *32*(23), 2749-2755. https://doi.org/10.1080/14786419.2017.1380024

Zhou, A., Zhang, C., & Zhang, M. (2008). A New Protostane Triterpenoid from the Rhizome of Alisma orientale. *Chinese Journal of Natural Medicines*, *6*(2), 109-111. https://doi.org/10.1016/s1875-5364(09)60011-x
